# Organocatalytic Asymmetric Synthesis of Azabicyclo[2.1.1]hexanes

**DOI:** 10.1021/acscatal.5c02225

**Published:** 2025-05-02

**Authors:** Layth Alama, Nils Frank, Lennart Brücher, Johanna Nienhaus, Benjamin List

**Affiliations:** Max-Planck-Institut für Kohlenforschung, Kaiser-Wilhelm-Platz 1, 45470 Mülheim an der Ruhr, Germany

**Keywords:** BCB, azabicyclohexane, chiral, bioisosteric, organocatalysis

## Abstract

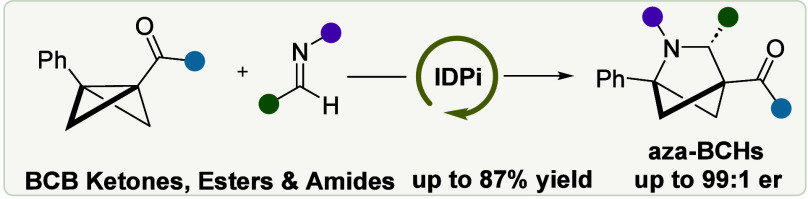

The bioisosteric replacement of 2D aromatic scaffolds with sp^3^-rich surrogates has become an important design element in modern medicinal chemistry. Within the sp^3^-rich world, the azabicyclo[2.1.1]hexane (aza-BCH) scaffold stands out as a pyrrolidine replacement and a useful building block. However, despite recent advancements in the field, a modular enantioselective synthesis of aza-BCHs remains challenging. In this study, we introduce an asymmetric organocatalytic approach relying on a confined imidodiphosphorimidate (IDPi) Brønsted acid that catalyzes the formal cycloaddition reaction of bicyclo[1.1.0]butanes (BCBs) with *N*-aryl imines under mild conditions, generating chiral aza-BCHs with high enantioselectivity (up to 99:1 er). Notably, the reaction proceeds effectively with a range of BCBs featuring ester, ketone, and amide functionalities. Experimental studies suggest that the reaction proceeds via a stepwise mechanism.

## Introduction

**Figure 1:**
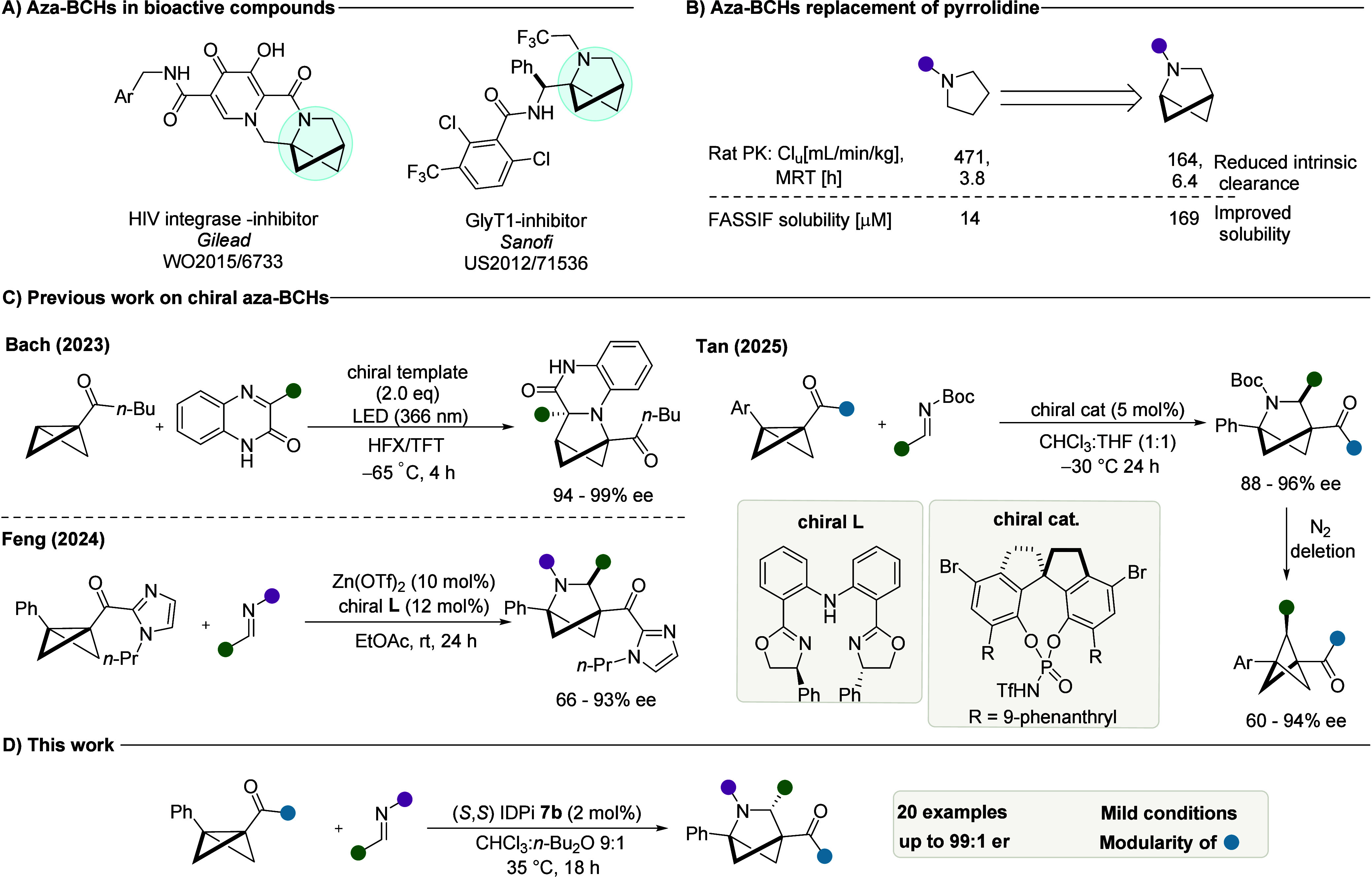
(A) Bioactive compounds with an aza-BCH scaffold. (B) Compression of aza-BCHs and pyrrolidine on drug properties. (C) Previous reports on chiral aza-BCHs. (D) Schematic representation of this work.

Azabicyclo[2.1.1]hexanes (aza-BCHs) have received considerable recent attention from both organic and medicinal chemists.^1^ The rigid, sp^3^-rich scaffold has found recent applications in drug discovery, and leading pharmaceutical companies have developed bioactive derivatives (Figure 1A). Their unique ring system makes aza-BCHs of special importance in the design of pharmaceuticals within the framework of the “escape from flatland” concept, which states that increasing the saturation of a drug candidate by incorporating complex sp^3^-rich scaffolds can significantly improve its physiochemical and pharmacokinetic properties.^2,3^ In 2018, Mykhailiuk reported that aza-BCHs could be employed as bioisosteres of pyrrolidines, which exist in 37 FDA-approved drugs, and provide enhanced metabolic stability, water solubility, and reduced lipophilicity.^4,5^ Moreover, a recent optimization study by Merck for the LRRK2 kinase inhibitor for the treatment of Parkinson’s disease (Figure 1B) found that consistent improvement in intrinsic clearance and solubility can be achieved by replacing the pyrrolidine unit with an aza-BCH, making these building blocks of high value.^6^

The state-of-the-art synthesis of aza-BCHs was pioneered in 2022 by Leitch et al.,^7^ who reported a Lewis acid-catalyzed [3 + 2] formal cycloaddition of bicyclo[1.1.0]butanes (BCBs) with *N*-aryl imines. Building on this work, several cycloaddition reactions of BCBs with aldehydes, ketenes, and other components were subsequently reported by Glorius,^8−13^ Studer,^14^ Feng,^15^ and others providing access to all-carbon and oxabicyclohexanes,^1,16−26^ However, all of these methods gave only racemic products. Additionally, imines were also investigated in [3 + 2] formal cycloadditions with cyclopropanes.^27,28^

Bach (Figure 1C) reported the first asymmetric example of a photocycloaddition of monosubstituted BCB ketones with quinoxalinones.^29^ Two equivalents of a chiral template were required to achieve high enantioselectivity in two reported examples of aza-BCHs. Broadly applicable catalytic asymmetric approaches toward BCHs are currently emerging.^30−32^ During the preparation of this Letter, two reports were published. First, Feng reported a formal cycloaddition reaction with zinc triflate as a Lewis acid catalyst equipped with a chiral ligand affording the products with high er (96.5:3.5) under mild conditions.^33^ Despite this work, the use of a directing group strategy severely limits the modularity to a single type of BCB ketones. Second, Tan reported a different approach employing a chiral Brønsted acid catalyst for the formal cycloaddition of BCBs with *N*-Boc imines to yield chiral aza-BCHs followed by nitrogen deletion relying on the memory of chirality to generate valuable chiral bicyclo[1.1.1]pentanes (BCPs) with high er (98:2).^34^

In spite of these advancements, asymmetric formal cycloaddition reactions of BCB amides remain elusive to date. Amides can be considered a cornerstone in modern medicinal chemistry, as reflected for example by amide bond formation being the most frequently used reaction.^35^ A strategy to directly furnish chiral aza-BCH amides from BCB amides without additional steps therefore appeared to be highly desirable to us. The present study addresses this gap and offers a unified asymmetric approach for converting BCB esters, ketones, and amides into the corresponding aza-BCHs (Figure 1D), with a focus on Weinreb amides due to their synthetic versatility.^36^

## Results and Discussion

Our aim has been to develop a single approach toward the formal cycloaddition reaction of BCBs with different electron-withdrawing groups. We investigated *N*-aryl imine as the reaction partner due to two reasons: (a) *N*-aryl imines exhibit high reactivity toward aza-BCHs under Lewis acid catalysis, as reported by Leitch,^7^ and (b) an analysis of bioactive compounds containing pyrrolidine units found that 92% of pyrrolidine-based drugs have substitution on the nitrogen atom, with a high interest in *N*-aryl pyrrolidine.^37,38^ At the onset of our study, we decided to screen a variety of chiral organic Brønsted acids with our model substrates, BCB amide **1a** and *N*-benzylideneaniline (**2a**). We found that the imidodiphosphorimidate (IDPi)-type catalyst enabled superior catalytic performance in terms of reactivity and selectivity, whereas imidodiphosphates (IDPs) showed minimal to no reactivity and *N*-triflylphosphoramides delivered a mixture of products (see the Supporting Information) with low yield and enantioselectivity of the desired product **3a**.

**Figure 2:**
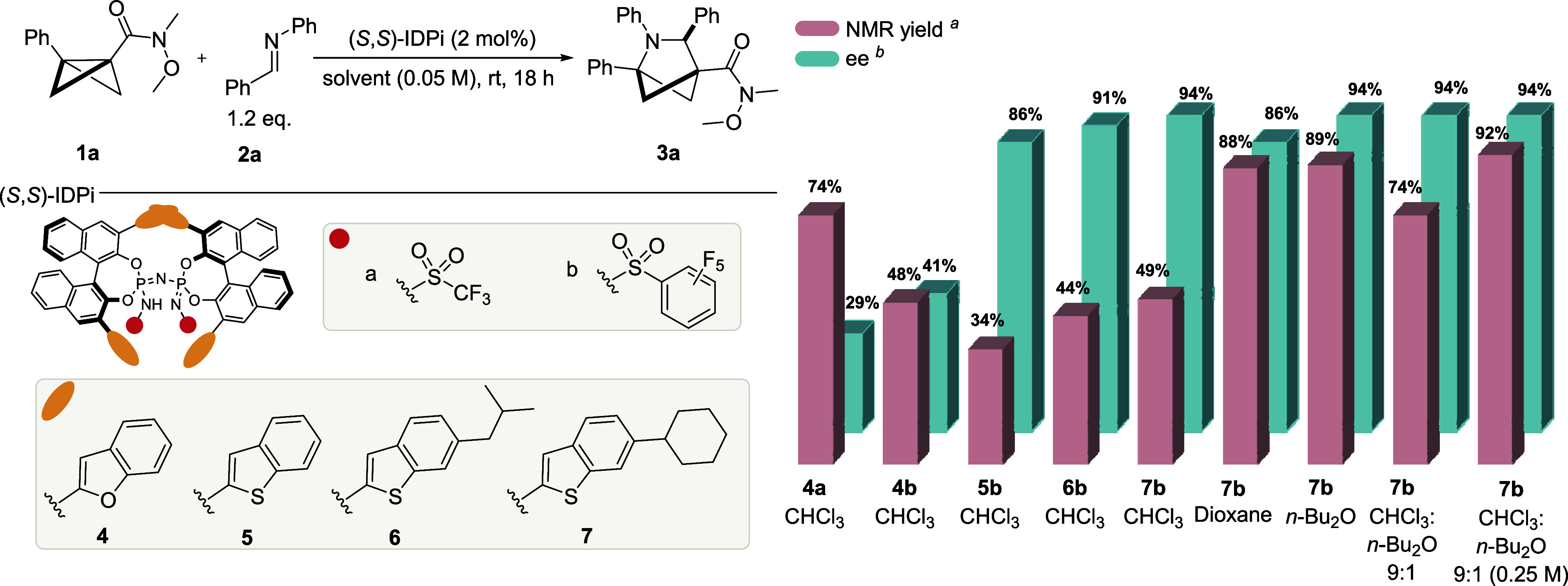
Optimization of the formal cycloaddition reaction of a BCB amide with an *N*-aryl imine. Reactions were performed on a 0.05 mmol scale. ^*a*^Yields were determined by ^1^H NMR analysis of the crude reaction mixtures using mesitylene as an internal standard. ^*b*^ee values were determined by chiral HPLC.

Upon further investigations, it was found that IDPis with heteroaromatic wings, a catalyst class that was recently reported by our laboratory for asymmetric Pictet–Spengler reactions,^39,40^ performed best. Changing the core of benzofuran catalyst **4a** from a −CF_3_ group to an aromatic −C_6_F_5_ group (**4b**) provided a 12% increase in the enantioselectivity. To our delight, combining the aromatic core with a benzothiophene wing in catalyst **5b** resulted in a significant jump in the selectivity (Figure 2).

Encouraged by this result, a further wing modification to install a cyclohexyl group at C6 of the benzothiophene ring (**7b**) afforded the product with a significantly improved er of 97:3. The evaluation of different solvents showed that CHCl_3_ gave a higher selectivity; however, the conversion still remained low in comparison to ether solvents such as 1,4 dioxane and dibutyl ether, where higher product conversion rates were observed with a slightly lower enantioselectivity (89% ee). Fine-tuning of CHCl_3_ and dibutyl ether mixtures and a concentration optimization furnished excellent results of 92% yield with 97:3 er. Furthermore, a slight increase in reaction temperature to 35 °C was necessary to achieve full conversion of the BCB starting material.

**Figure 3:**
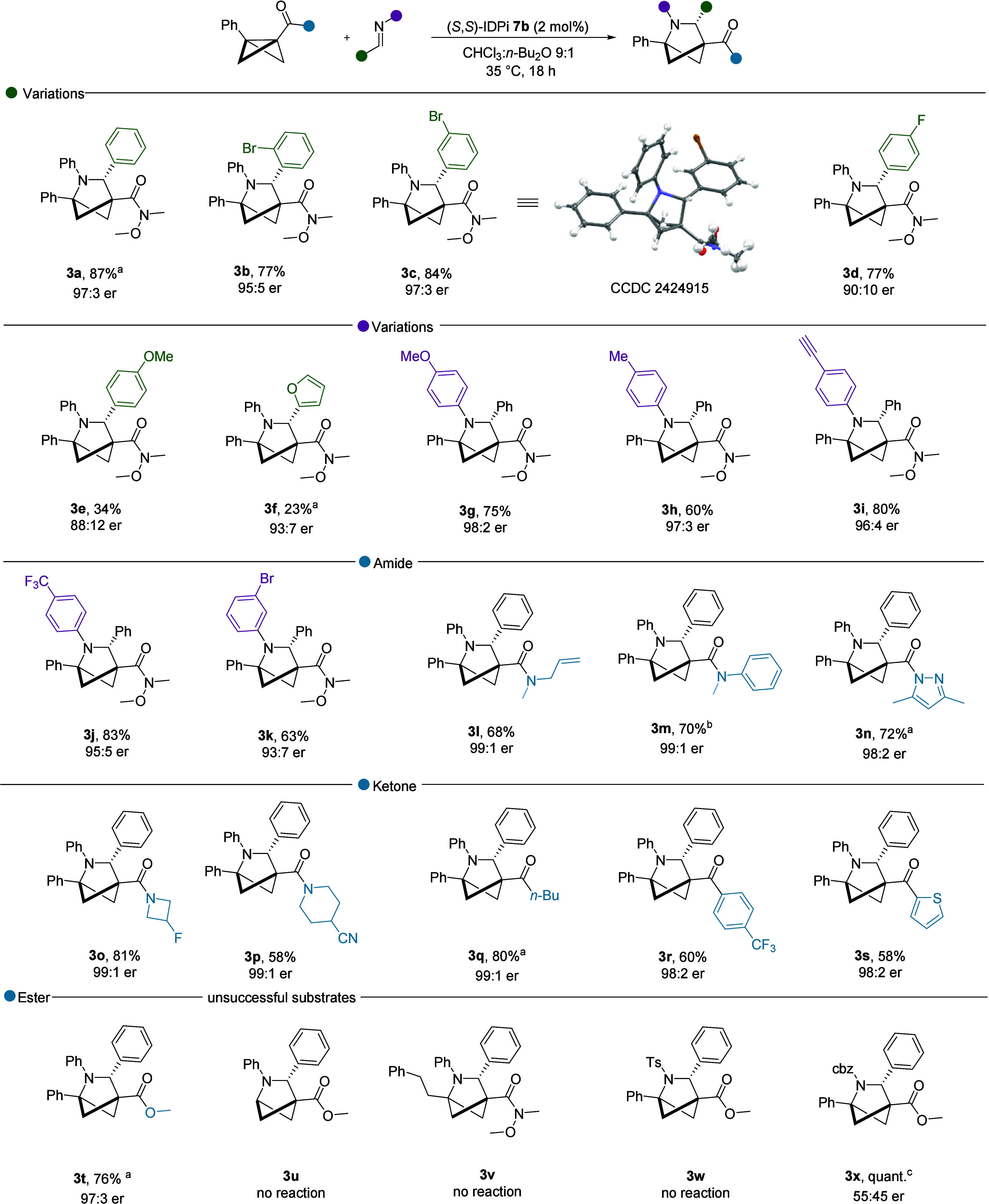
Scope of the formal cycloaddition reaction of BCBs and *N*-aryl imines. All reactions were conducted on a 0.25 mmol scale. Isolated yields after purification are reported. ^a^Reaction at rt. ^b^Reaction on a 0.20 mmol scale. ^*c*^Yield determined by ^1^H NMR analysis of the crude reaction mixture using mesitylene as an internal standard.

With the optimized conditions in hand, we turned our attention to exploring the substrate scope and limitations of the reaction (Figure 3). First, we investigated *N*-aryl imines with different modifications on the aromatic ring. A bromo substituent at either the *ortho* or *meta* position of the phenyl ring was well-tolerated, delivering products **3b** and **3c** in high yields and enantioselectivity. Substituents at the *para* position can also readily be utilized and, depending on their nature, furnish products with varying enantioselectivity. For example, having a *p*-fluoro substituent gave product **3d** with 90:10 er, whereas the electron-withdrawing *p*-CF_3_ group resulted in an er of 60:40. Installing an *p*-methoxy group gave product **3e** with 88:12 er in a lower yield of 34%. Finally, a furan substituent delivered product **3f** with 93:7 er in a low yield of 23% due to furan polymerization.

To our delight, PMP-imine was well-tolerated and delivered the corresponding product **3g** in 75% yield with high enantioselectivity (98:2 er). Similarly, a variety of *N*-aryl groups were tolerated. Both electron-donating and electron-withdrawing groups at the *para* and *meta* positions of this substituent delivered the product with good enantioselectivity (**3h**–**3k**). We next shifted our focus to the electron-withdrawing group of the BCB starting material. Acyclic secondary aliphatic **3l** and aromatic **3m** amides were well-tolerated, and we obtained the desired products with an exceptional er of 99:1. Both aromatic amide **3n** and cyclic aliphatic amides **3o** and **3p** proved to be accessible with our reaction, with excellent enatioselectivity. Remarkably, under the same conditions, ketones can also be used as substrates. Aliphatic ketone **3q**, aromatic ketone **3r**, and heteroaromatic ketone **3s** were all obtained with excellent enantioselectivity. The absolute configuration of product **3c** was determined to be *R* via an X-ray analysis. Finally, BCB ester **3t** was obtained in good yield with 97:3 er.

**Scheme 1:**
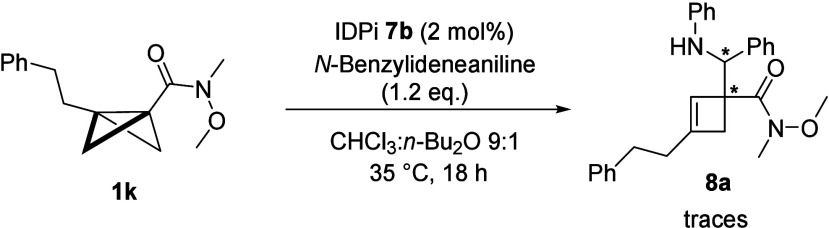
Reaction of an Alkyl-Substituted BCB with *N*-Benzylideneaniline

Current limitations of our reaction were also identified. In the case of monosubstituted BCB, no reaction to give the product **3u** was observed. Similarly, an alkyl substituent instead of the phenyl group did not lead to the formation of product **3v**. In this case, a small amount of cyclobutene side product **8a** could be identified (Scheme 1). Additionally, investigating imines with different *N*-protecting groups (see the Supporting Information) showed that a Ts group leads to no reaction to form the desired cycloaddition product **3w**, while carbamate-derived groups such as *N*-Boc and *N*-Cbz gave the corresponding products in quantitative yields with low enantioselectivity (60:40 and 55:45 er, respectively). Next, compound **3a** was subjected to 1,2-addition using *n*-butyllithium to assess the stability of the stereochemical information. The transformation proceeded smoothly to afford the corresponding ketone **3q** with 97:3 er, indicating no loss of chiral information.

### Mechanistic Proposal

Based on our observations, previous reports,^7,34^ and the result shown in Scheme 1, we conclude that the reaction proceeds via a stepwise rather than a concerted mechanism. In the absence of the benzylic stabilization of the initially generated carbocation, the formation of the cyclobutenyl amine **8a** is observed with no formation of the desired product **3v**. In the case of the monosubstituted BCBs, we speculate that they are insufficiently nucleophilic to engage with the protonated imine.

**Figure 4:**
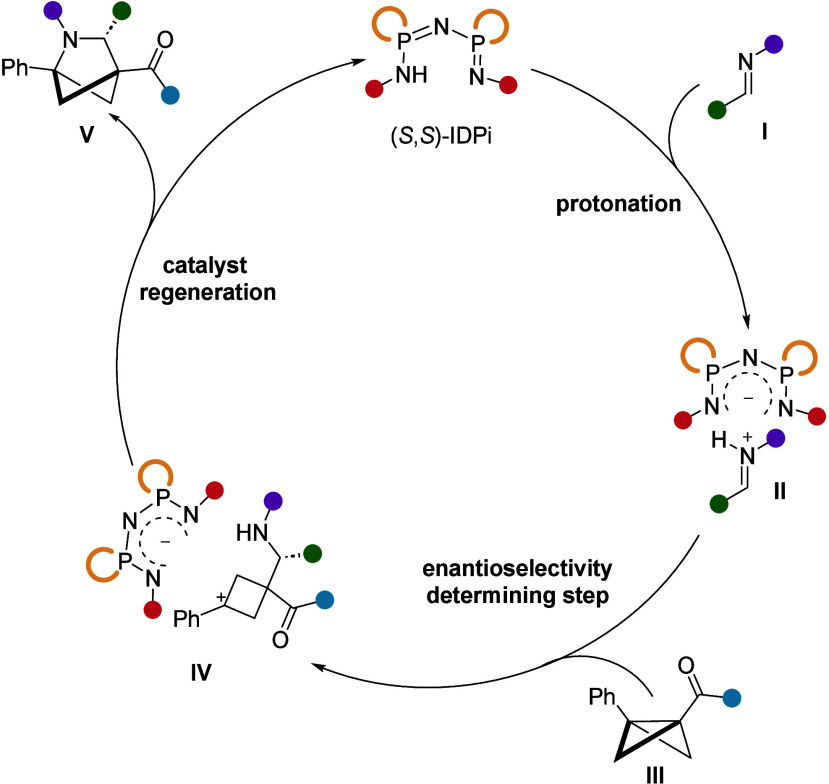
Proposed reaction mechanism.

A plausible reaction mechanism is shown in Figure 4. The IPDi catalyst acts as a Brønsted acid, activating imine **I** by protonation. This results in the formation of ion pair intermediate **II** with the IDPi anion, creating a chiral confined environment around the iminium cation. Next, an enantioselective nucleophilic attack from BCB **III** onto iminium intermediate **II** sets the stereochemistry and provides the stabilized benzylic cation intermediate **IV**, which undergoes a nucleophilic attack by the nitrogen, thereby closing the ring and delivering the product **V** while regenerating the active catalyst. If the carbocation in **IV** is not stable enough, it undergoes deprotonation, yielding the corresponding cyclobutenyl amine, as in the case of compound **8a**.

## Conclusion

In summary, we have developed an asymmetric organocatalytic Brønsted acid-catalyzed formal cycloaddition approach for the synthesis of aza-BCHs with modularity of the electron-withdrawing group of the BCB, achieving high levels of enantioselectivity (up to 99:1 er) across a range of substrates with our privileged electron-rich IDPi catalyst **7b**.

## Supporting Information Available

Crystallographic data for **3c** (CIF)
Experimental details, analytical data for all new compounds, NMR spectra, and HPLC chromatograms (PDF)

## Supplementary Material

cs5c02225_si_001.cif

## Abbreviations

*n*-Bu_2_O: dibutyl ether
CHCl_3_: chloroform
PMP: *p*-methoxyphenyl
FDA: U.S. Food and Drug Administration
ee: enantiomeric excess
er: enantiomeric ratio
