# Supplementary material for: Organocatalytic Asymmetric Synthesis of Azabicyclo[2.1.1]hexanes
Source: ACS Catal. 2025 May 2;15(10):8297–302. doi: 10.1021/acscatal.5c02225 (PMC12090186; doi:10.1021/acscatal.5c02225)
Supplement: Supplementary file 2 — cs5c02225_si_002.pdf [file cs5c02225_si_002.pdf]

Supplementary Material

Organocatalytic Asymmetric Synthesis of Aza Bicyclo[2.1.1]hexanes

Layth Alama, Nils Frank, Lennart Brücher, Johanna Nienhaus & Benjamin List*

Max-Planck-Institut für Kohlenforschung, Kaiser-Wilhelm-Platz 1, 45470 Mülheim an der Ruhr, Germany

*e-mail: list@kofo.mpg.de

Contents

1 Materials and Methods 2

2 Synthesis of Substrates 3

2.1 BCB synthesis 3

2.2 Imine synthesis. 4

3 Reaction Development 5

4 Failed and underdeveloped Substrates 6

5 IDPi Catalyst Synthesis : 7

6 General procedure for the synthesis of aza-BCHs (GP) 12

7 Crystallographic Data of 3c 23

8 NMR Spectra 27

9 HPLC Chromatograms 64

10 References 85

1 Materials and Methods

General Information

Unless otherwise stated, all reagents were purchased from commercial suppliers and used without further purification. All solvents used in the reactions were distilled from appropriate drying agents prior to use. Reactions were monitored by thin layer chromatography (TLC) on silica gel pre-coated plastic sheets (0.2 mm, Macherey-Nagel). Visualization was accomplished by irradiation with UV light at 254 nm. Column chromatography was performed on Merck silica gel (60, particle size 0.040–0.063 mm). ^{1}H and ^{13}C NMR spectra were recorded on a Bruker AV-500 spectrometer in deuterated solvents. Proton chemical shifts are reported in ppm (δ) relative to the solvent resonance employed as the internal standard ($CDCl_3$ and CD_2Cl_2 δ 7.26 and 5.32 respectively). Data are reported as follows: chemical shift, multiplicity (s = singlet, d = doublet, t = triplet, q = quartet, p = pentet, s = sextet, h = heptet, m = multiplet, br = broad), coupling constants (Hz) and integration. ^{13}C chemical shifts are reported in ppm with the solvent resonance as the internal standard ($CDCl_3$ and CD_2Cl_2 δ 77.16 and 54.00 respectively). High resolution mass spectra were determined on a Bruker APEX III FTMS (7 T magnet). Optical rotations were determined with an Autopol IV polarimeter (Rudolph Research Analytical) at 589 nm and 25 °C. Data are reported as follows: $[\alpha]_\lambda^{temp}$, concentration (*c* in mg/1 mL), and solvent. Enantiomeric ratios (er) were determined by HPLC analysis employing a chiral stationary phase column specified in the individual experiment, by comparing the samples with the appropriate racemic mixtures.

Solvents

Solvents were dried by distillation from appropriate drying agents1 in the technical department of the Max-Planck-Institut für Kohlenforschung and received in Schlenk flasks under an atmosphere of argon. Dry solvents were stored over dry 4 Å molecular sieves.

Nomenclature

Nomenclature follows the suggestions proposed by the computer program ChemBioDraw (12.0.3.1216) of CBD/Cambridgesoft.

2 Synthesis of Substrates

2.1 BCB synthesis

BCBs reported in the manuscript

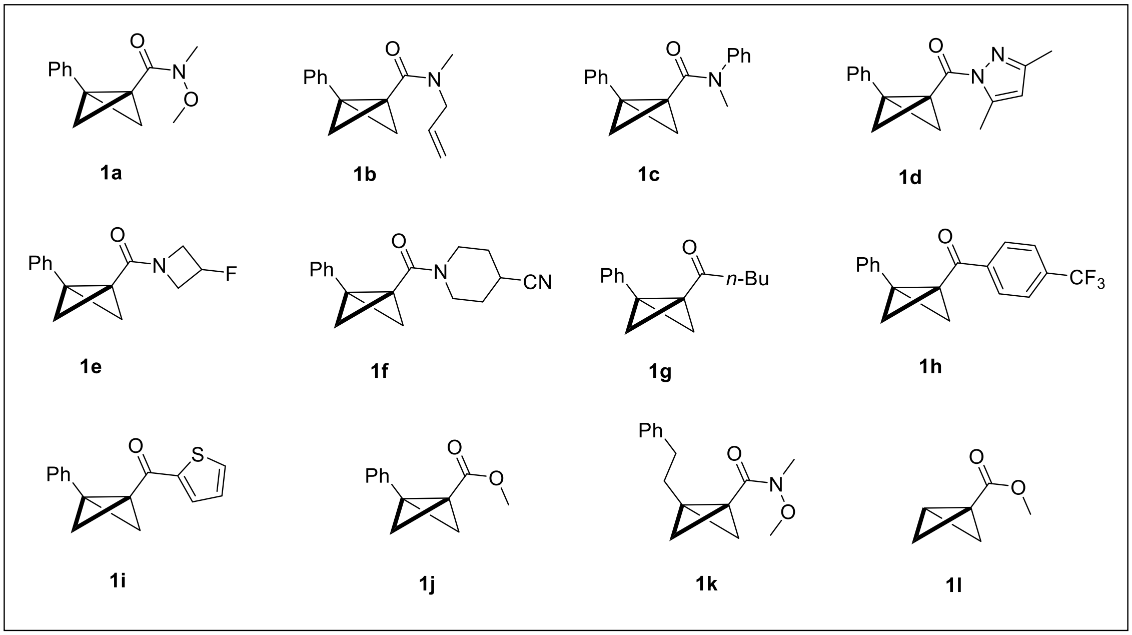

Figure SI- 1: BCBs reported in this study.

BCBs **1a**,**1j**, **1c**, **1d**, **1g-1j**, and **1l** were synthesized according to known literature procedures. [1-3]

BCBs **1b**, **1e**, **1f** and **1k** were synthesized analog to a reported procedures.[1, 4]

***N*-allyl-*N*-methyl-3-phenylbicyclo[1.1.0]butane-1-carboxamide (1b)**:

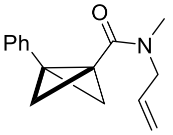

^{1}H NMR (501 MHz, CD_2Cl_2) δ 7.35–7.17 (m, 5H), 5.98–5.52 (m, 1H), 5.26–4.98 (m, 2H), 4.33–4.05 (m, 1H), 3.98–3.69 (m, 1H), 3.19–3.04 (m, 1H), 2.80 (s, 2H), 2.85–2.65 (m, 2H), 1.64–1.45 (m, 2H). **^{13}C NMR** (126 MHz, CD_2Cl_2) δ 135.1, 134.8, 128.7, 127.0, 126.8, 116.7, 53.3, 37.0, 36.4, 33.7, 22.5. **HRMS m/z (ESI):** calculated for $C_{15}H_{17}NONa$ $[M+Na]^+$: 250.1202, found 250.1204.

(3-fluoroazetidin-1-yl)(3-phenylbicyclo[1.1.0]butan-1-yl)methanone (1e):

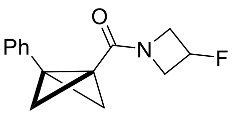

^{1}H NMR (501 MHz, CD_2Cl_2) δ 7.36–7.20 (m, 5H), 5.35–5.18 (m, 1H), 4.67–3.78 (m, 4H), 2.76 (t, *J* = 0.9 Hz, 2H), 1.56 (s, 2H). **^{13}C NMR** (126 MHz, CD_2Cl_2) δ 170.6, 134.7, 128.9, 127.2, 126.7, 83.4 (d, *J* = 203.6 Hz), 35.9, 31.6, 22.4. **^{19}F NMR** (471 MHz, CD_2Cl_2) δ -180.73. **HRMS m/z (EI):** calculated for $C_{14}H_{14}NOF$ $[M]^+$: 231.1054, found 231.1054.

1-(3-phenylbicyclo[1.1.0]butane-1-carbonyl)piperidine-4-carbonitrile (1f):

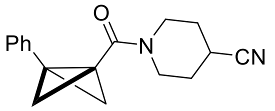

^{1}H NMR (501 MHz, CD_2Cl_2) δ 7.30 (d, *J* = 4.3 Hz, 4H), 7.27–7.18 (m, 1H), 4.05–3.19 (m, 4H), 2.86 (tt, *J* = 8.1, 4.1 Hz, 1H), 2.73 (s, 2H), 1.98–1.65 (m, 4H), 1.60 (s, 2H). **^{13}C NMR** (126 MHz, CD_2Cl_2) δ 168.0, 134.8, 128.7, 127.0, 126.9, 121.6, 36.9, 31.3, 27.1, 21.9. **HRMS m/z (EI):** calculated for $C_{17}H_{18}N_2O_1$ $[M]^+$: 266.1415, found 266.1414.

***N*-methoxy-*N*-methyl-3-phenethylbicyclo[1.1.0]butane-1-carboxamide (1k)**:

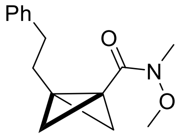

^{1}H NMR (501 MHz, CD_2Cl_2) δ 7.30 – 7.24 (m, 2H), 7.24–7.14 (m, 3H), 3.68 (s, 3H), 3.19 (s, 3H), 2.78–2.71 (m, 2H), 2.21 (s, 2H), 2.19–2.13 (m, 2H), 1.16 (s, 2H). **^{13}C NMR** (126 MHz, CD_2Cl_2) δ 171.8, 142.3, 129.0, 128.8, 126.4, 61.6, 38.3, 35.0, 34.3, 29.7, 29.6, 12.7.**HRMS m/z (ESI):** calculated for $C_{15}H_{19}NO_2Na$ $[M+Na]^+$: 268.1308, found 268.1306.

2.2 Imine synthesis.

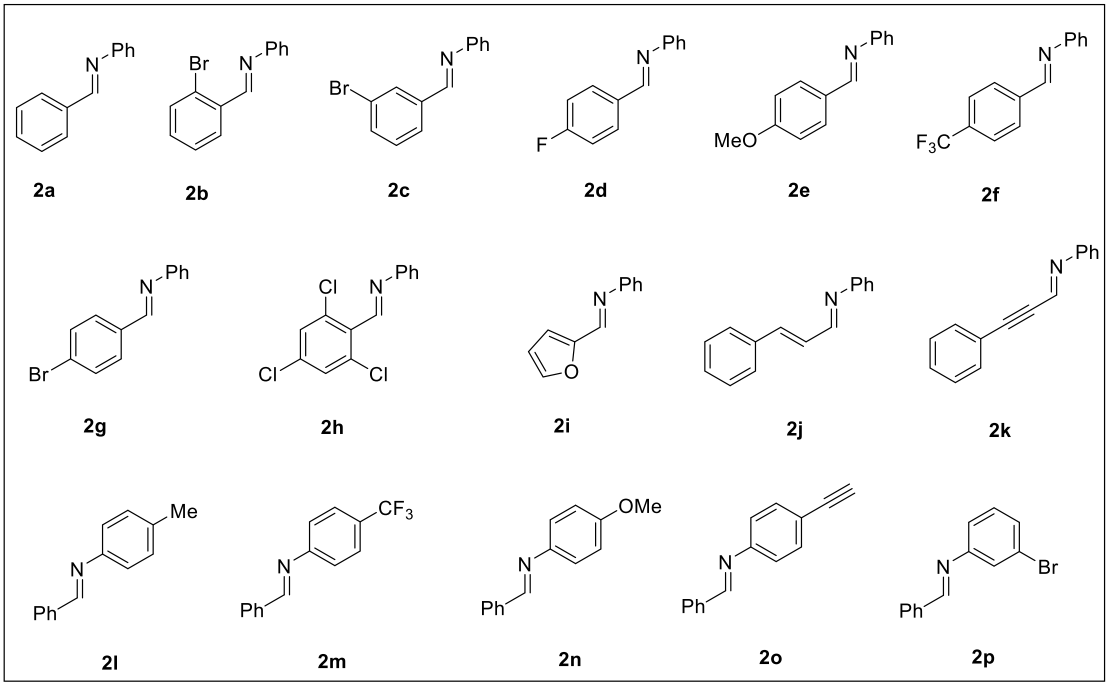

Figure SI- 2: imine reported in this study.

Imine **2a** and **2e** are commercially available and were used as received. Imine **2b**-**2d** and **2f**-**2p** are literature known and were synthesized analog to a known literature procedure.[5]

3 Reaction Development

Reaction optimization procedure.

N-methoxy-*N*-methyl-3-phenylbicyclo[1.1.0]butane-1-carboxamide **1a** (0.05 mmol, 1.0 eq.) and *N*-Benzylidenanilin **2a** (0.6 mmol, 1.2 eq.) and the appreciate catalyst (0.001 mmol, 2 mol%) were weighed in a GC vial and placed under Ar. Reaction mixture was dissolved in 1 mL dry $CHCl_3$ (0.05 M) and allowed to stir at RT for 18 h. the Reaction was quenched with 20 µL Et_3N. Mesitylen (0.05 mmol, 1 eq.) was added as an internal standard. The yield was determined by 1H NMR analysis of the crude mixture. The remaining sample purified by a preparative TLC, and e.r. value was determined by chiral HPLC.

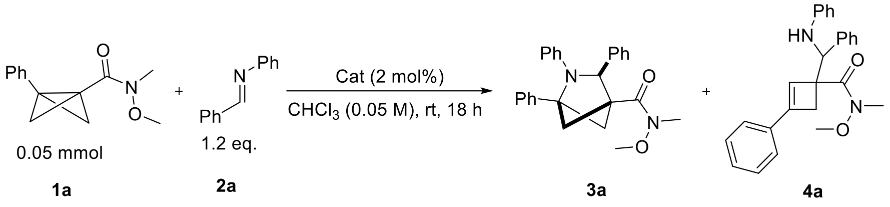

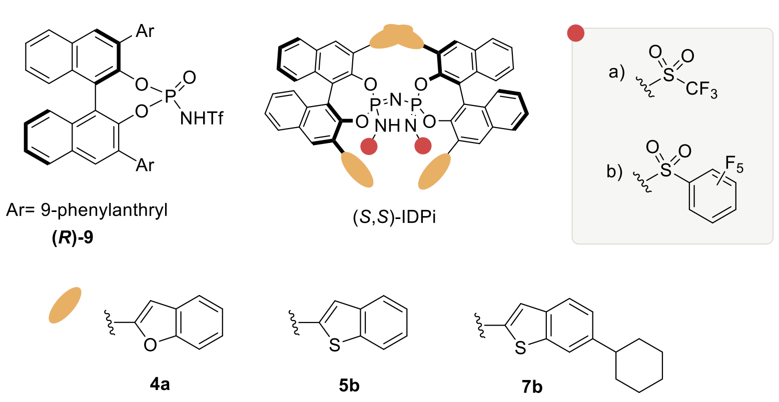

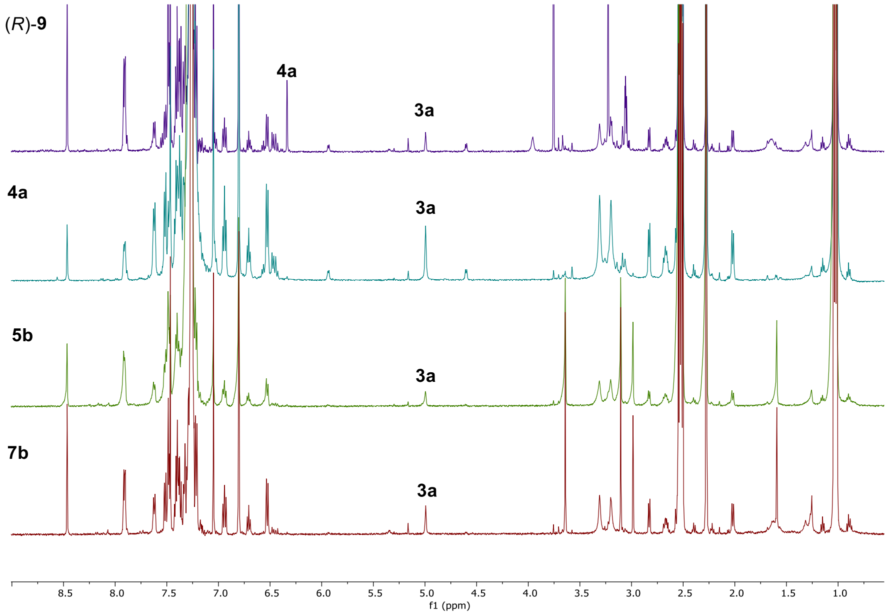

Figure SI- 3: 1H-NMR of crude reactions mixtures with different catalysts.

Table SI-1: solvent screening with catalyst **7b**.

ENTRY	BCB	SOLVENT [0.25 M]	YIELD (3A)	ER (3A)
**1**	1j	CPME	72%	94:6
**2**	1j	Dioxane	90%	95:5
**3**	1j	Dibutylether	68%	97.5:2.5
**4**	1j	$CHCl_3$:Dioxane 9:1	79%	96:4
**5**	1j	$CHCl_3$:Dibuytlether 9:1	86%	97:3
**6**	1a	$CHCl_3$:Dibuytlether 9:1	83%	97:3
**7**	1d	$CHCl_3$:Dibuytlether 9:1	90%	96:4
**8**	1g	$CHCl_3$:Dibuytlether 9:1	70%	99:1

4 Failed and underdeveloped Substrates

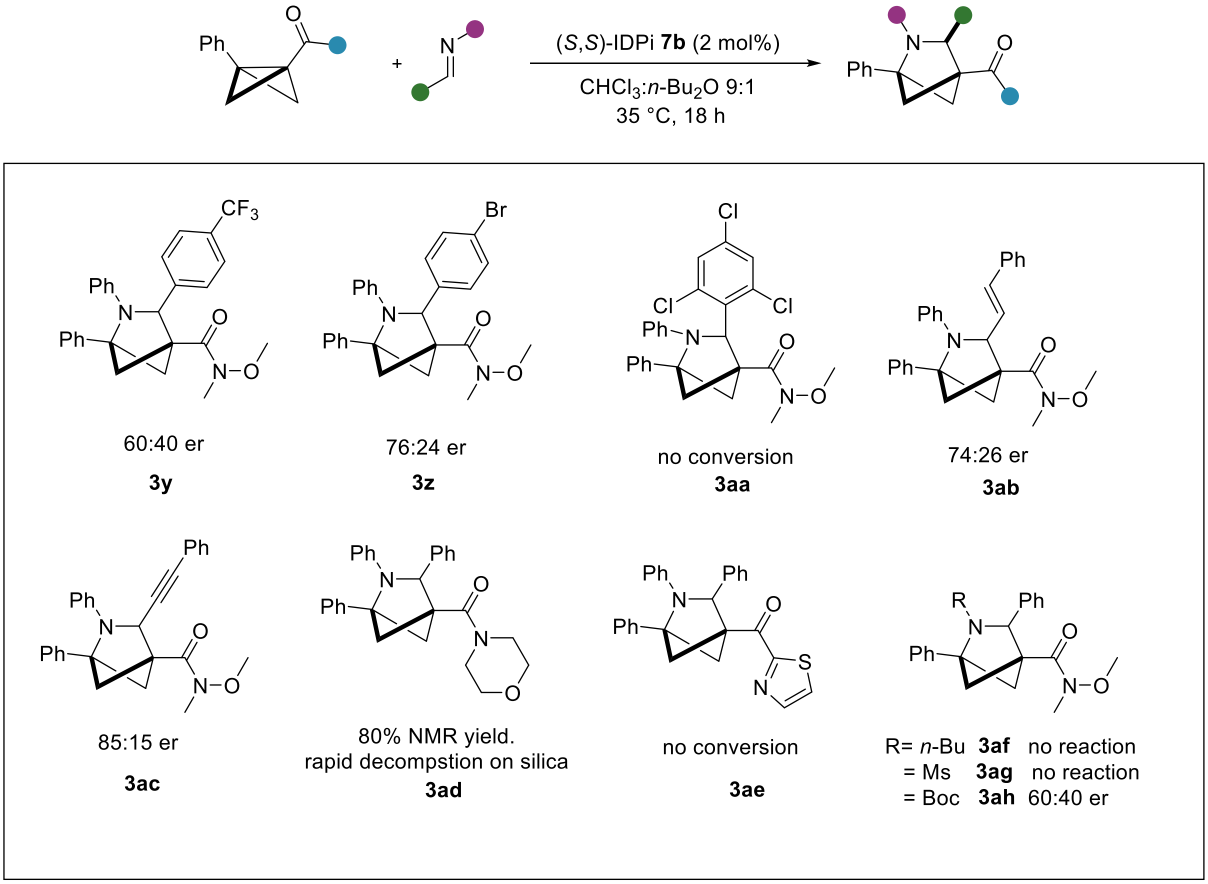

Figure SI- 4: Failed and underdeveloped substrates. Yields determined by ^{1}H NMR analysis of the crude reaction mixture using mesitylene as an internal standard.er determined by chiral HPLC.

5 IDPi Catalyst Synthesis :

Catalysts **4a** & **4b** were synthesized according to a previously reported procedure.[6] Catalyst **6b** was synthesized analog to the following representative procedure.

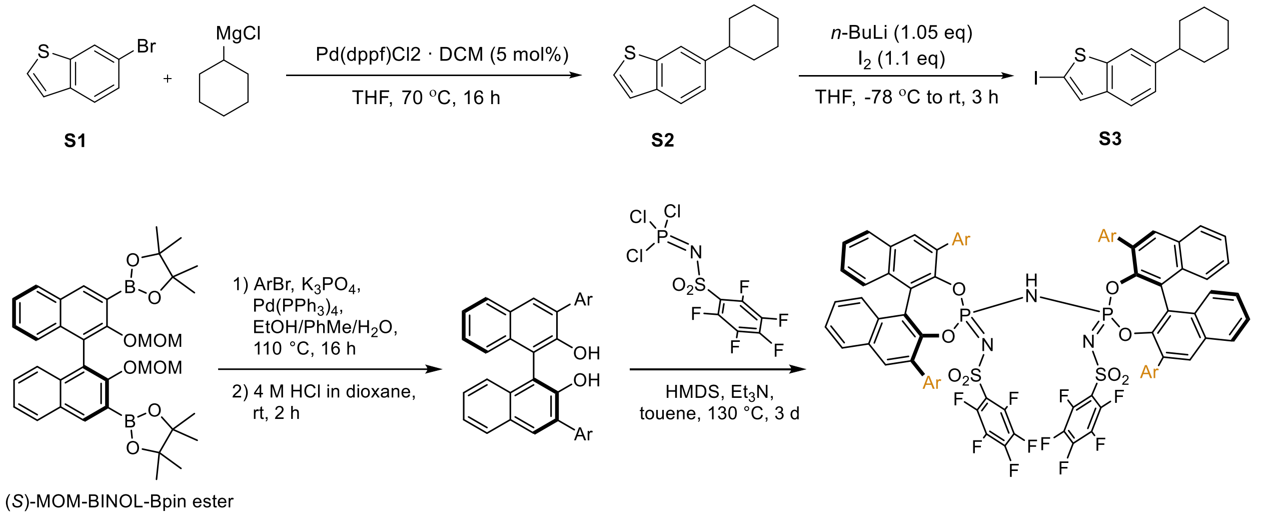

Scheme SI-1: representative reaction scheme to afford IDPi **7b**.

6-cyclohexylbenzo[b]thiophene (S2): a flame dried Schlenck tube under argon atmosphere was charged with 6-bromobenzo[b]thiophene S1 (5 g, 23.5 mmol, 1 eq.) and Pd(dppf)Cl_2 · CH_2Cl_2 (958 mg, 1.2 mmol, 5 mol%). Solids were dissolved in 44 mL anhydrous THF (0.5 M). A solution of cyclohexylmagnesiumchlorid (1M in THF, 47 mL, 47 mmol, 2 eq.) was added slowly to the reaction mixture at rt. Tube was sealed and allowed to stir at 70 °C for 12 h. after full consumption of starting material (TLC control) reaction was cooled to 0 °C and quenched with saturated aqueous solution of NH_4Cl and the aqueous layer was extracted with EtOAc (3x). The combined organic phases were dried over anhydrous Na_2SO_4, filtrated and concentrated under reduced pressure. The crude material was passed through a plug of silica eluting with 100% n-Pentane to afford the product as a colorless solid **S2** (4 g, 18.7 mmol, 80%).

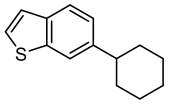

^{1}H NMR (501 MHz, $CDCl_3$) δ 7.77–7.71 (m, 2H), 7.36 (dd, J = 5.3, 1.2 Hz, 1H), 7.31–7.27 (m, 1H), 7.24 (dd, J = 8.2, 1.6 Hz, 1H), 2.63 (tt, J = 12.0, 3.5 Hz, 1H), 1.99–1.91 (m, 2H), 1.91–1.84 (m, 2H), 1.82–1.75 (m, 1H), 1.54–1.38 (m, 4H), 1.34–1.26 (m, 1H).

^{13}C NMR (126 MHz, $CDCl_3$) δ 144.7, 140.2, 137.9, 125.5, 124.2, 123.7, 123.4, 120.1, 44.8, 34.9, 27.1, 26.3.

HRMS m/z (EI): calculated for $C_{14}H_{16}S$ $[M]^+$: 216.0967, found 216.0972.

6-cyclohexyl-2-iodobenzo[b]thiophene (S3): a flame dried Schlenck flask under argon atmosphere was charged with

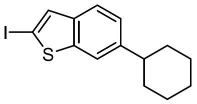

6-cyclohexyl-4,5-dihydrobenzo[b]thiophene S2 (4 g, 18.7 mmol, 1 eq.) and 55 mL anhydrous THF (0.33 M). The reaction mixture was cooled to -78 °C, *n*-BuLi (2.5 M in n-Hexane, 7.8 mL, 19.6 mmol, 1.05 eq.) was added dropwise and stirred for 45 min at -78 °C. Iodine (5.2 g, 20 mmol, 1.1 eq.) was added in one go as a 1 M solution in THF. Cooling bath was removed and the reaction was allowed to stir at RT for 2 h. Reaction was quenched with a saturated aqueous solution of NH_4Cl and the aqueous layer was extracted with EtOAc (3x). The combined organic phases were washed with a saturated aqueous solution of $Na_2S_2O_3$ (3x), dried over anhydrous Na_2SO_4, filtrated and concentrated under reduced pressure to afford the product **S3** as a colorless solid (6 g, 17.6 mmol, 94%).

^{1}H NMR (501 MHz, CD_2Cl_2) δ 7.63 (d, *J* = 8.2 Hz, 1H), 7.61 (d, *J* = 0.8 Hz, 1H), 7.49 (d, *J* = 0.7 Hz, 1H), 7.19 (dd, *J* = 8.2, 1.6 Hz, 1H), 2.61 (tt, *J* = 11.6, 3.4 Hz, 1H), 1.94–1.81 (m, 4H), 1.80–1.72 (m, 1H), 1.51–1.37 (m, 4H), 1.33–1.26 (m, 1H).

^{13}C NMR (126 MHz, CD_2Cl_2) δ 145.6, 145.2, 139.6, 134.2, 124.9, 122.5, 119.4, 77.3, 45.2, 35.2, 27.5, 26.7.

HRMS m/z (EI): calculated for $C_{14}H_{15}SI$ $[M]^+$: 341.9934, found 341.9940.

(*S*)-3,3'-bis(6-cyclohexylbenzo[b]thiophen-2-yl)-[1,1'-binaphthalene]-2,2'-diol (S4): A flame dried Schlenk flask

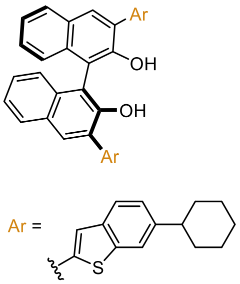

with a reflux condenser under argon atmosphere was charged with (S)-MOM-BINOL Bpin ester (4 g, 6.4 mmol, 1 eq.), 6-cyclohexyl-2-iodobenzo[b]thiophene **S3** (5.4 g, 16 mmol, 2.5 eq.), $Pd(PPh_3)_4$ (369 mg, 0.3 mmol, 5 mol%) and K_2CO_3 (4.4 g, 32 mmol, 5 eq.). Flask was vacuum flashed with argon (3x) and degassed 1,4 dioxane (30 mL) and H_2O (15 mL) were added. Reaction was stirred at 100 °C for 16 h. After cooling to room temperature, the organic layer was separated and the aqueous phase was extracted with ethyl acetate (3x). The organic phase was combined, filtered through a thin layer of silica gel, and the silica gel layer was washed with additional ethyl acetate. The solvent was removed under reduced pressure and the crude MOM-protected diol was obtained. Subsequently, the crude product was dissolved in DCM (20 mL). A solution of HCl (4 M in 1,4-dioxane, 25 mL) was added at room temperature and the mixture was stirred for 16 h. The solvent was evaporated under reduced pressure and the crude product was purified by column chromatography (5% to 10% EtOAc in pentane v/v) to afford the corresponding diol **S4** (3.8 g, 5.4 mmol, 84%).

^{1}H NMR (501 MHz, CD_2Cl_2) δ 8.40 (s, 2H), 7.99 (d, *J* = 8.1 Hz, 2H), 7.95 (s, 2H), 7.79–7.66 (m, 4H), 7.47–7.39 (m, 2H), 7.36–7.29 (m, 2H), 7.25 (dd, *J* = 8.2, 1.5 Hz, 2H), 7.16 (d, *J* = 8.4 Hz, 2H), 5.78 (s, 2H), 2.65 (tt, *J* = 11.6, 3.4 Hz, 2H), 1.99–1.83 (m, 8H), 1.82–1.74 (m, 2H), 1.57–1.41 (m, 8H), 1.36–1.26 (m, 2H).

^{13}C NMR (126 MHz, CD_2Cl_2) δ 150.7, 145.9, 140.6, 139.3, 138.8, 133.5, 131.2, 130.0, 129.2, 128.4, 125.3, 124.9, 124.7, 124.6, 124.2, 124.2, 120.1, 112.9, 45.4, 35.3, 27.5, 26.8.

HRMS m/z (ESI): calculated for $C_{48}H_{41}O_2S_2$ $[M-H]^-$: 713.2554, found 713.2564.

***N*,*N*'-((4*S*,4'*S*)-azanediylbis(2,6-bis(6-cyclohexylbenzo[b]thiophen-2-yl)-4l5-dinaphtho[2,1-d:1',2'-f][1,3,2]dioxaphosphepine-4-yl-4-ylidene))bis(2,3,4,5,6-pentafluorobenzenesulfonamide)—methane (1/2)** (**7b**): In a flame dried Schlenk flask under argon, Phosphorimidoyl trichloride (501 mg, 1.3 mmol, 1 eq.) and Diol **S4** (938 mg, 1.3 mmol, 1 eq.) was added. The solids were dissolved in anhydrous toluene (13 mL) and then trimethylamine (1.5 mL, 10.5 mmol, 8 equiv.) was added at room temperature. The mixture was stirred at room temperature for 2 h and then hexamethyldisilazane (134 µL, 0.64 mmol, 0.49 eq.) was added. After being stirred at room temperature for additional 10 min, the mixture was heated to 130 °C for 3 d. The reaction mixture was cooled to room temperature, diluted with DCM and HCl (10% aq.). Two phases were separated and the aqueous layer was washed with DCM (2x50 mL). The combined organic layer was dried with Na_2SO_4, filtered and concentrated in vacuum. Purification by silica gel column chromatography (2% EtOAc in toluene v/v). The product was acidified by dissolving in 10 mL CH_2Cl_2 and stirring with 30 mL 6 M HCl solution for 1 h. organic phase was separated and solvent removed under reduced pressure to afford the product **7b** as light gray solid (730 mg, 0.36 mmol, 56%).

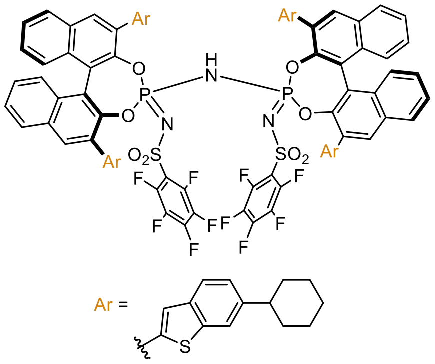

^{1}H NMR (501 MHz, CD_2Cl_2) δ 8.24 (s, 2H), 8.03 (dt, *J* = 8.4, 1.6 Hz, 4H), 7.86–7.79 (m, 2H), 7.76 (d, *J* = 8.2 Hz, 2H), 7.67–7.53 (m, 6H), 7.50 (d, *J* = 8.6 Hz, 2H), 7.36–7.27 (m, 4H), 7.26–7.19 (m, 4H), 7.14 (d, *J* = 2.0 Hz, 4H), 7.06 (d, *J* = 8.2 Hz, 2H), 6.64 (dd, *J* = 8.2, 1.6 Hz, 2H), 6.32 (s, 2H), 2.65 (tt, *J* = 11.8, 3.3 Hz, 2H), 2.02–1.85 (m, 10H), 1.80–1.73 (m, 2H), 1.59–1.41 (m, 14H), 1.36–1.23 (m, 6H), 1.18–1.01 (m, 6H), 0.94–0.81 (m, 4H), 0.61–0.38 (m, 2H).

^{13}C NMR (126 MHz, CD_2Cl_2) δ 146.4, 145.7, 141.4, 139.5, 139.2, 137.6, 136.1, 135.8, 132.5, 132.4, 132.2, 131.9, 129.7, 129.6, 129.1, 128.1, 127.9, 127.7, 127.3, 127.2, 127.0, 126.3, 125.3, 124.9, 124.6, 124.4, 124.0, 123.5, 122.5, 119.6, 119.4, 45.4, 44.4, 35.1, 34.7, 33.7, 27.6, 27.4, 27.3, 26.8, 26.5 (other signals not detected or observed).

^{31}P NMR (203 MHz, CD_2Cl_2) δ –18.25.

^{19}F NMR (471 MHz, CD_2Cl_2) δ –136.37 to –137.20 (m, 2F), –145.98 to –147.21 (m, 1F), –159.61 (t, *J* = 21.1 Hz, 2F).

HRMS m/z (ESI): calculated for $C_{108}H_{80}O_8N_3F_{10}S_6P_2$ $[M-H]^-$: 1990.3591, found 1990.3611.

6-isobutylbenzo[b]thiophene (S5):

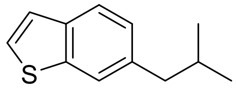

^{1}H NMR (501 MHz, $CDCl_3$) δ 7.73 (d, J = 8.1 Hz, 1H), 7.66 (dt, J = 1.6, 0.7 Hz, 1H), 7.36 (d, J = 5.5 Hz, 1H), 7.30 (dd, J = 5.3, 0.8 Hz, 1H), 7.17 (dd, J = 8.2, 1.5 Hz, 1H), 2.61 (d, J = 7.3 Hz, 2H), 1.94 (dp, J = 13.6, 6.7 Hz, 1H), 0.94 (d, J = 6.6 Hz, 6H).

^{13}C NMR (126 MHz, $CDCl_3$) δ 140.0, 138.2, 137.8, 126.1, 125.4, 123.7, 123.2, 122.6, 45.6, 30.6, 22.5.

HRMS m/z (EI): calculated for C12H14S [M]+: 190.0811, found 190.0814.

2-iodo-6-isobutylbenzo[b]thiophene (S6):

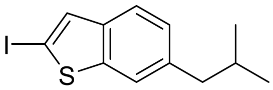

^{1}H NMR (501 MHz, $CDCl_3$) δ 7.60 (d, J = 8.1 Hz, 1H), 7.53 (dt, J = 1.5, 0.7 Hz, 1H), 7.48 (d, J = 0.8 Hz, 1H), 7.10 (dd, J = 8.2, 1.5 Hz, 1H), 2.56 (d, J = 7.2 Hz, 2H), 1.91 (dp, J = 13.6, 6.8 Hz, 1H), 0.92 (d, J = 6.6 Hz, 6H).

^{13}C NMR (126 MHz, $CDCl_3$) δ 144.7, 139.0, 138.5, 133.7, 126.3, 121.9, 121.3, 45.5, 30.6, 22.5.

HRMS m/z (EI): calculated for C12H13SI [M]+: 315.9777, found 315.9782.

(*S*)-3,3'-bis(6-isobutylbenzo[b]thiophen-2-yl)-[1,1'-binaphthalene]-2,2'-diol (S7):

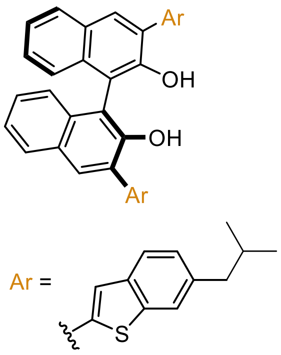

^{1}H NMR (501 MHz, CD_2Cl_2) δ 8.40 (s, 2H), 8.00 (d, J = 8.2 Hz, 2H), 7.96 (s, 2H), 7.73 (d, J = 8.2 Hz, 2H), 7.67 (d, J = 1.3 Hz, 2H), 7.43 (ddd, J = 8.1, 6.8, 1.2 Hz, 2H), 7.33 (ddd, J = 8.2, 6.8, 1.3 Hz, 2H), 7.18 (ddd, J = 12.6, 8.3, 1.3 Hz, 4H), 5.79 (br s, 2H), 2.62 (d, J = 7.2 Hz, 4H), 1.96 (dp, J = 13.6, 6.8 Hz, 2H), 0.95 (d, J = 6.6 Hz, 12H).

^{13}C NMR (126 MHz, CD_2Cl_2) δ 150.7, 140.4, 139.5, 139.2, 138.7, 133.5, 131.2, 130.0, 129.2, 128.4, 126.9, 125.3, 124.8, 124.6, 124.2, 124.0, 122.5, 112.9, 46.1, 31.1, 22.7.

HRMS m/z (ESI): calculated for C44H37O2S2 [M–H]–: 661.2241, found 661.2247.

_N_,_N_'-((4S,4'S)-azanediylbis(2,6-bis(6-isobutylbenzo[b]thiophen-2-yl)-4l5-dinaphtho[2,1-d:1',2'-f][1,3,2]dioxaphosphepine-4-yl-4-ylidene))bis(2,3,4,5,6-pentafluorobenzenesulfonamide)--methane (1/2) (6b):

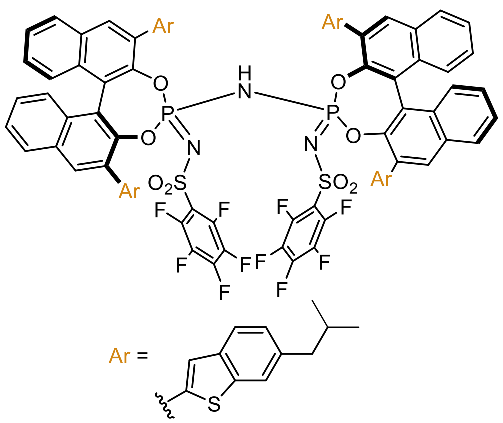

^{1}H NMR (501 MHz, CD_2Cl_2) δ 8.23 (s, 2H), 8.03 (t, J = 8.8 Hz, 4H), 7.85 (t, J = 7.6 Hz, 2H), 7.74 (d, J = 8.2 Hz, 2H), 7.64 (t, J = 7.7 Hz, 2H), 7.61–7.54 (m, 4H), 7.46 (d, J = 8.7 Hz, 2H), 7.38–7.28 (m, 4H), 7.24 (d, J = 8.5 Hz, 2H), 7.20–7.06 (m, 6H), 7.00 (s, 2H), 6.63 (s, 2H), 6.57 (d, J = 8.0 Hz, 2H), 2.62 (d, J = 7.2 Hz, 4H), 2.07 (dd, J = 13.4, 6.6 Hz, 2H), 1.96 (hept, J = 6.7 Hz, 2H), 1.80 (dd, J = 13.5, 7.8 Hz, 2H), 1.35–1.26 (m, 4H), 0.96 (dd, J = 6.6, 3.5 Hz, 12H), 0.55 (d, J = 6.6 Hz, 6H), 0.49 (d, J = 6.6 Hz, 6H).

^{13}C NMR (126 MHz, CD_2Cl_2) δ 139.9, 139.5, 132.5, 129.9, 129.6, 129.1, 127.9, 127.7, 127.4, 127.2, 126.9, 126.5, 126.3, 125.3, 124.7, 123.4, 122.0, 121.8, 46.1, 45.6, 31.0, 30.5, 22.7, 22.6, 22.2. (other signals not detected or observed).

^{19}F NMR (471 MHz, CD_2Cl_2) δ -136.64 (d, J = 20.9 Hz, 2F), -146.50 (s, 1F), -159.81 (t, J = 20.7 Hz, 2F).

^{31}P NMR (203 MHz, CD_2Cl_2) δ -17.69.

HRMS m/z (ESI): calculated for C100H72O8N3F10S6P2 [M–H]–: 1886.2965, found 1886.2962.

6 General procedure for the synthesis of aza-BCHs (GP)

An oven-dried glass vial equipped with a magnetic stir bar was charged with the appropriate BCB (0.25 mmol, 1 eq.), imine (0.30 mmol, 1.2 eq.) and IDPi **7b** (0.001 mmol, 2 mol%). Vial was placed under Ar gas and sealed. A mixture of $CHCl_3$:*n*-Bu_2O 9:1 (0.25 M) was added. The resulting reaction mixture was stirred for 18 h at 35 °C. Subsequently, the reaction was quenched by the addition of NEt_3 at rt. The crude reaction mixture was diluted with 5 mL CH_2Cl_2 and directly purified *via* flash column chromatography.

Racemic traces were obtained by using racemic IDPi in THF or 1,4-dioxane (0.25 M).

***N*-methoxy-*N*-methyl-1,2,3-triphenyl-2-azabicyclo[2.1.1]hexane-4-carboxamide (3a):** Prepared according to GP, purified *via* flash column chromatography (20% EtOAc in pentane) to afford **3a** in 87% yield as white solid.

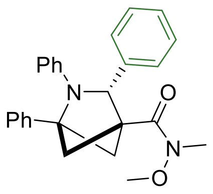

^{1}H NMR (501 MHz, CD_2Cl_2) δ 7.61 (d, *J* = 7.5 Hz, 2H), 7.57–7.50 (m, 2H), 7.41 (dt, *J* = 19.2, 7.5 Hz, 4H), 7.32 (dt, *J* = 19.8, 7.4 Hz, 2H), 6.97–6.88 (m, 2H), 6.69 (t, *J* = 7.3 Hz, 1H), 6.53 (d, *J* = 8.1 Hz, 2H), 4.96 (s, 1H), 3.47 (s, 3H), 3.18 (s, 3H), 2.87 (d, *J* = 6.7 Hz, 1H), 2.65 (dd, *J* = 9.9, 6.7 Hz, 1H), 2.50 (dd, *J* = 9.8, 7.8 Hz, 1H), 1.94 (d, *J* = 7.8 Hz, 1H).

^{13}C NMR (151 MHz, CD_2Cl_2) δ 171.1, 151.9, 141.7, 140.7, 129.1, 128.7, 128.0, 127.8, 127.6, 126.3, 74.8, 70.3, 61.9, 55.6, 47.1, 44.9, 32.8.

HRMS m/z (ESI): calculated for $C_{26}H_{27}N_2O_2$ $[M+H]^+$: 399.2067, found 399.2068.

The enantiomeric ratio was measured by HPLC analysis: 150 mm Chiralpak IK-3R, 4.6 mm i.D., 3µm, MeOH/water = 85:15, flow rate 1 mL/min, 298K, λ = 220 nm, t_R = 5.8 min (major) and t_R = 7.2 min (minor). er = 96.9:3.1.

$[\alpha]_D^{25}$ = –234 (*c* 2.0, $CHCl_3$).

3-(2-bromophenyl)-*N*-methoxy-*N*-methyl-1,2-diphenyl-2-azabicyclo[2.1.1]hexane-4-carboxamide (3b):

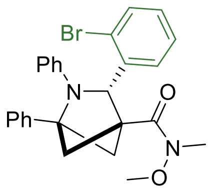

Prepared according to GP, purified *via* flash column chromatography (30% EtOAc in pentane) to afford **3b** in 77% yield as white solid.

^{1}H NMR (501 MHz, CD_2Cl_2) δ 8.21 (dd, *J* = 7.8, 1.8 Hz, 1H), 7.60 (dd, *J* = 7.8, 1.3 Hz, 1H), 7.57–7.52 (m, 2H), 7.48 (td, *J* = 7.6, 1.3 Hz, 1H), 7.44–7.36 (m, 2H), 7.34–7.28 (m, 1H), 7.25 (td, *J* = 7.6, 1.8 Hz, 1H), 6.99–6.88 (m, 2H), 6.78–6.66 (m, 1H), 6.50–6.40 (m, 2H), 5.13 (s, 1H), 3.56 (s, 3H), 3.12 (s, 3H), 2.93 (d, *J* = 6.5 Hz, 1H), 2.72 (qd, *J* = 10.0, 6.9 Hz, 2H), 1.88 (d, *J* = 7.2 Hz, 1H).

^{13}C NMR (126 MHz, CD_2Cl_2) δ 171.6, 151.5, 140.6, 140.5, 133.3, 132.3, 129.7, 129.4, 128.9, 127.9, 127.8, 126.6, 123.5, 120.6, 119.2, 74.2, 70.0, 61.4, 54.9, 47.8, 45.1, 33.3.

HRMS m/z (ESI): calculated for $C_{26}H_{26}N_2O_2Br$ $[M+H]^+$: 477.1172, found 477.1175.

The enantiomeric ratio was measured by HPLC analysis: 150 mm Chiralpak IC-3R, 4.6 mm i.D., 3µm, MeCN/water = 65/35, 1 ml/min, 298K, λ = 220nm, t_R = 7.2 min (minor) and t_R = 9.4 min (major). er = 95.6:4.4**.**

$[\boldsymbol{\alpha}]_{\boldsymbol{D}}^{\mathbf{25}}$ = –133 (*c* 2.0, $CHCl_3$).

(*R*)-3-(3-bromophenyl)-*N*-methoxy-*N*-methyl-1,2-diphenyl-2-azabicyclo[2.1.1]hexane-4-carboxamide (3c):

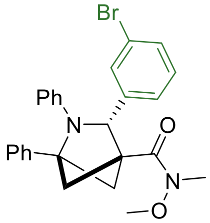

Prepared according to GP, purified *via* flash column chromatography (25% to 30% EtOAc in pentane) to afford **3c** in 84% yield as white solid.

^{1}H NMR (501 MHz, CD_2Cl_2) δ 7.79 (t, *J* = 1.9 Hz, 1H), 7.57–7.45 (m, 4H), 7.43–7.36 (m, 2H), 7.36–7.28 (m, 2H), 6.99–6.91 (m, 2H), 6.76–6.68 (m, 1H), 6.57–6.49 (m, 2H), 4.93 (s, 1H), 3.51 (s, 3H), 3.20 (s, 3H), 2.88 (d, *J* = 6.9 Hz, 1H), 2.65 (dd, *J* = 9.9, 6.9 Hz, 1H), 2.45 (dd, *J* = 9.9, 7.9 Hz, 1H), 1.97 (dd, *J* = 7.9, 1.2 Hz, 1H).

^{13}C NMR (126 MHz, CD_2Cl_2) δ 151.7, 144.4, 140.5, 131.3, 131.1, 130.5, 129.3, 128.9, 127.8, 126.9, 126.5, 123.1, 120.7, 119.5, 74.4, 70.6, 62.2, 55.8, 47.2, 45.1.

HRMS m/z (ESI): calculated for $C_{26}H_{26}N_2O_2Br$ $[M+H]^+$: 477.1172, found 477.1175.

The enantiomeric ratio was measured by HPLC analysis: 150 mm Chiralcel OJ-3R, 4.6 mm i.D., 3µm, MeCN/water = 55/45, 1 ml/min, 298K, λ = 220nm, t_R = 9.4 min (minor) and t_R = 10.6 min (major). er = 97.1:2.9.

$[\boldsymbol{\alpha}]_{\boldsymbol{D}}^{\mathbf{25}}$ = –208 (*c* 2.0, $CHCl_3$).

3-(4-fluorophenyl)-*N*-methoxy-*N*-methyl-1,2-diphenyl-2-azabicyclo[2.1.1]hexane-4-carboxamide (3d): Prepared according to GP, purified *via* flash column chromatography (25% to 30% EtOAc in pentane) to afford **3d** in 77% yield as white solid.

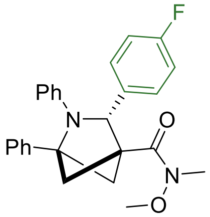

^{1}H NMR (501 MHz, CD_2Cl_2) δ 7.65–7.55 (m, 2H), 7.55–7.49 (m, 2H), 7.42–7.35 (m, 2H), 7.35–7.27 (m, 1H), 7.19–7.08 (m, 2H), 6.98–6.89 (m, 2H), 6.77–6.65 (m, 1H), 6.57–6.49 (m, 2H), 4.93 (s, 1H), 3.48 (s, 3H), 3.18 (s, 3H), 2.87 (d, *J* = 6.8 Hz, 1H), 2.65 (dd, *J* = 9.8, 6.9 Hz, 1H), 2.46 (dd, *J* = 9.9, 7.8 Hz, 1H), 1.96 (dd, *J* = 7.9, 1.3 Hz, 1H).

^{13}C NMR (126 MHz, CD_2Cl_2) δ 162.8 (d, *J* = 244.9 Hz), 151.9, 140.7, 137.7, 129.8 (d, *J* = 8.0 Hz), 129.3, 128.9, 127.8, 126.5, 120.6, 119.4, 115.6 (d, *J* = 21.3 Hz), 74.4, 70.5, 62.2, 55.7, 47.2, 45.0, 33.0.

^{19}F NMR (471 MHz, CD_2Cl_2) δ –116.28.

HRMS m/z (ESI): calculated for $C_{26}H_{25}N_2O_2FNa$ $[M+Na]^+$: 439.1792, found 439.1791.

The enantiomeric ratio was measured by HPLC analysis: 150 mm Chiralcel OJ-3R, 4.6 mm i.D., 3µm, MeCN/water = 70/30 1 ml/min, 298K, λ = 220, t_R = 4.7 min (minor) and t_R = 5.3 min (major). er = 90.2:9.8.

$[\boldsymbol{\alpha}]_{\boldsymbol{D}}^{\mathbf{25}}$ = –177 (c 2.0, $CHCl_3$).

***N*-methoxy-3-(4-methoxyphenyl)-*N*-methyl-1,2-diphenyl-2-azabicyclo[2.1.1]hexane-4-carboxamide (3e):** Prepared according to GP, purified *via* flash column chromatography (25% to 30% EtOAc in pentane) to afford **3e** in 34% yield as white solid.

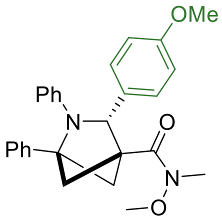

^{1}H NMR (501 MHz, CD_2Cl_2) δ 7.56–7.47 (m, 4H), 7.42–7.35 (m, 2H), 7.32–7.27 (m, 1H), 7.00–6.94 (m, 2H), 6.94–6.88 (m, 2H), 6.72–6.65 (m, 1H), 6.57–6.48 (m, 2H), 4.90 (s, 1H), 3.83 (s, 3H), 3.49 (s, 3H), 3.18 (s, 3H), 2.85 (d, *J* = 6.8 Hz, 1H), 2.62 (dd, *J* = 9.8, 6.8 Hz, 1H), 2.48 (dd, *J* = 9.9, 7.7 Hz, 1H), 1.93 (dd, *J* = 7.7, 1.3 Hz, 1H).

^{13}C NMR (151 MHz, CD_2Cl_2) δ 159.5, 152.0, 140.7, 133.6, 129.1, 129.0, 128.6, 127.5, 126.3, 120.1, 119.1, 114.0, 74.4, 70.2, 62.0, 55.6, 47.1, 44.7.

HRMS m/z (ESI): calculated for $C_{27}H_{29}N_2O_3$ $[M+H]^+$: 429.2173, found 429.2173.

The enantiomeric ratio was measured by HPLC analysis: 150 mm Chiralcel OZ-3R, 4.6 mm i.D., 3µm, MeCN/water = 65/35, 1 ml/min, 298K, λ = 220nm, t_R = 7.9 min (major) and t_R = 8.9 min (minor). er = 87.2:12.8.

$[\boldsymbol{\alpha}]_{\boldsymbol{D}}^{\mathbf{25}}$ = –178 (*c* 2.0, $CHCl_3$).

3-(furan-2-yl)-*N*-methoxy-*N*-methyl-1,2-diphenyl-2-azabicyclo[2.1.1]hexane-4-carboxamide (3f): Prepared according to GP, purified *via* flash column chromatography (25% EtOAc in pentane) to afford **3f** in 23% yield as white solid.

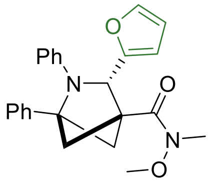

^{1}H NMR (501 MHz, CD_2Cl_2) δ 7.49–7.41 (m, 3H), 7.39–7.33 (m, 2H), 7.31–7.26 (m, 1H), 7.01–6.94 (m, 2H), 6.74 (tt, *J* = 7.4, 1.1 Hz, 1H), 6.69–6.62 (m, 3H), 6.46 (dd, *J* = 3.2, 1.8 Hz, 1H), 4.84 (s, 1H), 3.58 (s, 3H), 3.17 (s, 3H), 2.84 (dd, *J* = 4.6, 1.9 Hz, 1H), 2.57 (dd, *J* = 4.8, 2.0 Hz, 2H), 2.09–2.02 (m, 1H).

^{13}C NMR (151 MHz, CD_2Cl_2) δ 155.9, 151.6, 142.6, 140.2, 129.0, 128.7, 127.5, 126.4, 120.8, 119.8, 110.7, 109.1, 70.2, 69.4, 62.0, 54.4, 48.6, 43.9.

HRMS m/z (ESI): calculated for $C_{24}H_{24}N_2O_3Na$ $[M+Na]^+$: 411.1679, found 411.1679.

The enantiomeric ratio was measured by HPLC analysis: 150 mm Chiralcel OZ-3R, 4.6 mm i.D., 3µm, MeCN/water = 70/30, 1 ml/min, 298K, λ = 220 nm, t_R = 5.0 min (major) and t_R = 6.4 min (minor). er = 92.8:7.2.

$[\boldsymbol{\alpha}]_{\boldsymbol{D}}^{\mathbf{25}}$ = –195 (*c* 2.0, $CHCl_3$).

***N*-methoxy-2-(4-methoxyphenyl)-N-methyl-1,3-diphenyl-2-azabicyclo[2.1.1]hexane-4-carboxamide (3g):** Prepared according to GP, purified *via* flash column chromatography (20 to 25% EtOAc in pentane) to afford **3g** in 75% yield as white solid.

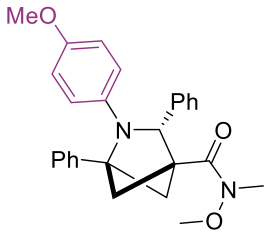

^{1}H NMR (501 MHz, CD_2Cl_2) δ 7.64 – 7.59 (m, 2H), 7.54 – 7.47 (m, 2H), 7.42 (dd, J = 8.3, 6.9 Hz, 2H), 7.39 – 7.25 (m, 5H), 6.57 – 6.48 (m, 4H), 4.88 (s, 1H), 3.62 (s, 3H), 3.46 (s, 3H), 3.17 (s, 3H), 2.84 (d, J = 6.8 Hz, 1H), 2.71 (dd, J = 9.8, 6.8 Hz, 1H), 2.48 (dd, J = 9.8, 7.7 Hz, 1H), 1.93 (d, 1H).

^{13}C NMR (151 MHz, CD_2Cl_2) δ 154.1, 145.2, 141.8, 140.6, 129.0, 128.6, 128.2, 127.7, 127.5, 126.7, 121.0, 114.0, 74.8, 70.4, 61.9, 55.6, 46.9, 44.8, 32.8.

HRMS m/z (ESI): calculated for $C_{27}H_{29}N_2O_3$ $[M+H]^+$: 429.2168, found 429.2172.

The enantiomeric ratio was measured by HPLC analysis: 150 mm Chiralcel OZ-3R, 4.6 mm i.D., 3µm, MeCN/water = 60/40, 1 ml/min, 298K, λ = 220 nm, t_R = 10.0 min (major) and t_R = 11.2 min (minor). er = 97.7:2.3.

$[\boldsymbol{\alpha}]_{\boldsymbol{D}}^{\mathbf{25}}$ = –219 (*c* 2.0, $CHCl_3$).

***N*-methoxy-*N*-methyl-1,3-diphenyl-2-(p-tolyl)-2-azabicyclo[2.1.1]hexane-4-carboxamide (3h):** Prepared according to GP, purified *via* flash column chromatography (25% EtOAc in pentane) to afford **3h** in 60% yield as white solid.

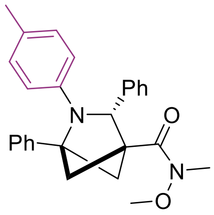

^{1}H NMR (501 MHz, CD_2Cl_2) δ 7.61 (d, *J* = 7.5 Hz, 2H), 7.56–7.48 (m, 2H), 7.43 (t, *J* = 7.6 Hz, 2H), 7.38 (t, *J* = 7.6 Hz, 2H), 7.34 (t, *J* = 7.4 Hz, 1H), 7.32–7.26 (m, 1H), 6.75 (d, *J* = 8.4 Hz, 2H), 6.50–6.39 (m, 2H), 4.91 (s, 1H), 3.47 (s, 3H), 3.18 (s, 3H), 2.86 (d, *J* = 6.8 Hz, 1H), 2.67 (dd, *J* = 9.8, 6.8 Hz, 1H), 2.48 (dd, *J* = 9.8, 7.7 Hz, 1H), 2.13 (s, 3H), 1.93 (dd, *J* = 7.7, 1.3 Hz, 1H).

^{13}C NMR (151 MHz, CD_2Cl_2) δ 171.2, 149.4, 141.8, 140.7, 129.7, 129.2, 129.0, 128.6, 128.1, 127.7, 127.5, 126.4, 74.8, 70.2, 61.9, 55.6, 47.1, 44.8, 32.8, 20.5.

HRMS m/z (ESI): calculated for $C_{27}H_{28}N_2O_2Na$ $[M+Na]^+$: 435.2043, found 435.2041.

The enantiomeric ratio was measured by HPLC analysis: 150 mm Chiralcel OZ-3R, 4.6 mm i.D., 3µm, MeCN/water = 60/40 1 ml/min, 298K, λ = 220 nm, t_R = 12.9 min (major) and t_R = 14.8 min (minor). er = 97.3:2.7.

$[\boldsymbol{\alpha}]_{\boldsymbol{D}}^{\mathbf{25}}$ = –215 (*c* 1.0, $CHCl_3$).

2-(4-ethynylphenyl)-*N*-methoxy-*N*-methyl-1,3-diphenyl-2-azabicyclo[2.1.1]hexane-4-carboxamide (3i): Prepared according to GP, purified *via* flash column chromatography (25% EtOAc in pentane) to afford **3i** in 80% yield as yellow solid.

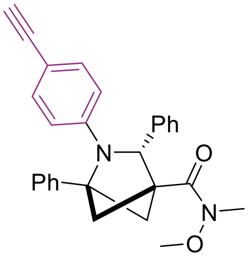

^{1}H NMR (501 MHz, CD_2Cl_2) δ 7.61 – 7.48 (m, 4H), 7.47 – 7.29 (m, 6H), 7.07 – 7.00 (m, 2H), 6.46 – 6.40 (m, 2H), 4.98 (s, 1H), 3.47 (s, 3H), 3.19 (s, 3H), 2.94 (s, 1H), 2.87 (d, J = 6.7 Hz, 1H), 2.58 (dd, J = 9.9, 6.8 Hz, 1H), 2.50 (dd, J = 9.9, 7.8 Hz, 1H), 1.96 (d, 1H).

^{13}C NMR (151 MHz, CD_2Cl_2) δ 170.8, 152.4, 141.0, 140.1, 132.6, 129.3, 128.8, 128.0, 127.9, 127.8, 126.3, 118.5, 112.8, 84.3, 75.7, 74.5, 70.6, 61.9, 55.5, 46.9, 45.1, 32.9.

HRMS m/z (ESI): calculated for $C_{28}H_{26}N_2O_2Na$ $[M+Na]^+$: 445.1886, found 445.1886.

The enantiomeric ratio was measured by HPLC analysis: 150 mm Chiralcel IB-3R, 4.6 mm i.D., 3µm, MeCN/water = 50/50, 1 ml/min, 298K, λ = 220 nm, t_R = 26.9 min (minor) and t_R = 29.3 min (major). er = 95.9:4.1.

$[\boldsymbol{\alpha}]_{\boldsymbol{D}}^{\mathbf{25}}$ = –242 (*c* 2.0, $CHCl_3$).

_N_-methoxy-_N_-methyl-1,3-diphenyl-2-(4-(trifluoromethyl)phenyl)-2-azabicyclo[2.1.1]hexane-4-carboxamide

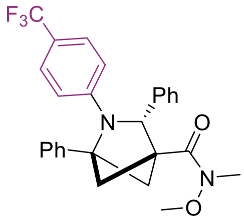

(3h): Prepared according to GP, purified *via* flash column chromatography (25% EtOAc in pentane) to afford **3h** in 83% yield as white solid.

^{1}H NMR (501 MHz, CD_2Cl_2) δ 7.59–7.50 (m, 4H), 7.48–7.39 (m, 4H), 7.39–7.31 (m, 2H), 7.15 (d, J = 8.7 Hz, 2H), 6.54 (d, J = 8.6 Hz, 2H), 5.03 (s, 1H), 3.47 (s, 3H), 3.20 (s, 3H), 2.88 (d, J = 6.0 Hz, 1H), 2.60–2.50 (m, 2H), 1.98 (dd, J = 7.2, 1.3 Hz, 1H).

^{13}C NMR (126 MHz, CD_2Cl_2) δ 154.9, 140.9, 140.1, 129.6, 129.1, 128.3, 128.2, 128.0, 126.5, 126.0 (q, J = 3.8 Hz), 125.3 (q, J = 270.2 Hz), 121.2 (q, J = 32.4 Hz), 118.2, 74.6, 70.9, 62.1, 55.6, 47.1, 45.3, 33.0.

^{19}F NMR (471 MHz, CD_2Cl_2) δ –61.8.

HRMS m/z (EI): calculated for $C_{27}H_{25}F_3N_2O_2Na$ $[M+Na]^+$: 489.1764, found 489.1760.

The enantiomeric ratio was measured by HPLC analysis: 150 mm Chiralcel IBN-3R, 4.6 mm i.D., 3µm, MeCN/water = 55/45 1 ml/min, 298K, λ = 220 nm, t_R = 18.1 min (minor) and t_R = 19.6 min (major). er = 95.4:4.6.

$[\alpha]_D^{25}$ = –180 (c 2.0, $CHCl_3$).

2-(3-bromophenyl)-_N_-methoxy-_N_-methyl-1,3-diphenyl-2-azabicyclo[2.1.1]hexane-4-carboxamide (3k):

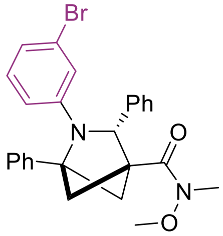

Prepared according to GP, purified *via* flash column chromatography (25% EtOAc in pentane) to afford **3k** in 83% yield as white solid.

^{1}H NMR (501 MHz, CD_2Cl_2) δ 7.60 – 7.49 (m, 4H), 7.47 – 7.31 (m, 6H), 6.84 – 6.75 (m, 2H), 6.63 – 6.59 (m, 1H), 6.47 – 6.41 (m, 1H), 4.97 (s, 1H), 3.48 (s, 3H), 3.19 (s, 3H), 2.86 (d, J = 6.9 Hz, 1H), 2.59 (dd, J = 9.9, 6.9 Hz, 1H), 2.49 (dd, J = 9.9, 7.9 Hz, 1H), 1.99 – 1.92 (m, 1H).

^{13}C NMR (126 MHz, CD_2Cl_2) δ 153.2, 140.9, 140.0, 130.0, 129.3, 128.8, 128.0, 127.9, 127.8, 126.3, 122.9, 122.3, 121.6, 117.6, 74.4, 70.6, 61.9, 55.5, 46.8, 44.9.

HRMS m/z (ESI): calculated for $C_{26}H_{25}N_2O_2BrNa$ $[M+Na]^+$: 499.0991, found 499.0992.

The enantiomeric ratio was measured by HPLC analysis: 150 mm Chiralcel IC-3R, 4.6 mm i.D., 3µm, MeCN/water = 50/50, 1 ml/min, 298K, λ = 220 nm, t_R = 32.7 min (major) and t_R = 35.0 min (minor). er = 93.1:6.9.

$[\alpha]_D^{25}$ = –242 (c 2.0, $CHCl_3$).

***N*-allyl-*N*-methyl-1,2,3-triphenyl-2-azabicyclo[2.1.1]hexane-4-carboxamide (3l):** Prepared according to GP, purified *via* flash column chromatography (30% EtOAc in pentane) to afford **3l** in 68% yield as white solid.

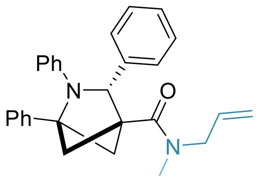

^{1}H NMR (501 MHz, CD_2Cl_2, 1:1 amide rotamers) δ 7.66–7.60 (m, 2H), 7.57–7.50 (m, 2H), 7.46–7.36 (m, 5H), 7.33–7.27 (m, 1H), 6.93 (tt, *J* = 7.3, 1.6 Hz, 2H), 6.70 (td, *J* = 7.3, 1.2 Hz, 1H), 6.54 (ddd, *J* = 7.6, 4.0, 2.2 Hz, 2H), 5.83–5.45 (m, 1H), 5.23–5.01 (m, 2H), 4.80 (d, *J* = 4.5 Hz, 1H), 4.02–3.85 (m, 1H), 2.83 and 2.46 (s, 3H), 2.81 (dd, *J* = 7.1, 4.5 Hz, 1H), 2.67 (ddd, *J* = 23.0, 9.9, 7.1 Hz, 1H), 2.54 (ddd, *J* = 30.1, 9.9, 7.6 Hz, 1H), 1.99 (d, *J* = 7.6 Hz, 1H).

^{13}C NMR (126 MHz, CD_2Cl_2) δ 169.9, 152.0, 141.5, 140.6, 140.5, 134.0, 133.5, 129.3, 128.9, 128.9, 128.2, 128.1, 128.0, 127.8, 126.5, 125.1, 120.5, 119.4, 119.4, 118.1, 117.4, 76.0, 75.7, 69.9, 69.8, 56.3, 56.1, 51.6, 51.2, 48.6, 48.6, 45.7, 45.3, 34.0, 33.9.

HRMS m/z (EI): calculated for $C_{28}H_{29}N_2O_1$ $[M+H]^+$: 409.2276, found 409.2274.

The enantiomeric ratio was measured by HPLC analysis: 150 mm Chiralcel OZ-3R, 4.6 mm i.D., 3µm, MeCN/water = 65/35 1 ml/min, 298K, λ = 220 nm, t_R = 12.0 min (major) and t_R = 14.2 min (minor). er = 98.7:1.3.

$[\boldsymbol{\alpha}]_D^{25}$ = –235 (*c* 2.0, $CHCl_3$).

***N*-methyl-*N*,1,2,3-tetraphenyl-2-azabicyclo[2.1.1]hexane-4-carboxamide (3m):** Prepared according to GP, purified *via* flash column chromatography (30% EtOAc in pentane) to afford **3m** in 70% yield as white solid.

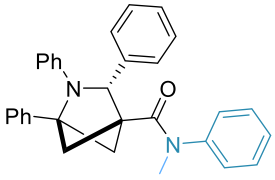

^{1}H NMR (501 MHz, CD_2Cl_2) δ 7.75–7.63 (m, 2H), 7.55–7.49 (m, 2H), 7.49–7.45 (m, 1H), 7.44–6.89 (m, 10H), 6.90–6.84 (m, 2H), 6.65 (t, *J* = 7.3 Hz, 1H), 6.44 (d, *J* = 8.3 Hz, 2H), 4.87 (s, 1H), 3.17 (s, 3H), 2.62 (d, *J* = 7.0 Hz, 1H), 2.41 (dd, *J* = 10.0, 7.0 Hz, 1H), 1.55 (dd, *J* = 10.0, 8.1 Hz, 1H), 1.13 (d, *J* = 8.0 Hz, 1H)..

^{13}C NMR (151 MHz, CD_2Cl_2) δ 169.5, 151.9, 143.1, 141.9, 140.4, 129.3, 128.9, 128.8, 128.6, 128.5, 128.1, 127.4, 126.2, 120.1, 119.2, 76.4, 70.1, 55.9, 48.0, 46.0, 38.7.

HRMS m/z (ESI): calculated for $C_{31}H_{28}N_2ONa$ $[M+Na]^+$: 467.2094, found 467.2098.

The enantiomeric ratio was measured by HPLC analysis: 150 mm Chiralpak IC-3R, 4.6 mm i.D., 3µm, MeCN/water = 80/20, 1 ml/min, 298K, λ = 220 nm, t_R = 6.1 min (major) and t_R = 7.4 min (minor). er = 98.7:1.3.

$[\boldsymbol{\alpha}]_D^{25}$ = –287 (*c* 2.0, $CHCl_3$).

(3,5-dimethyl-1H-pyrazol-1-yl)(1,2,3-triphenyl-2-azabicyclo[2.1.1]hexan-4-yl)methanone (3n): Prepared according to GP, purified *via* flash column chromatography (1 to 2% EtOAc in pentane) to afford **3n** in 72% yield as white solid.

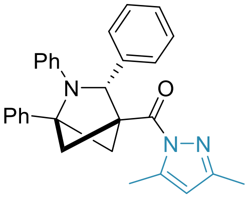

^{1}H NMR (501 MHz, CD_2Cl_2) δ 7.60–7.48 (m, 2H), 7.45–7.26 (m, 8H), 7.01–6.88 (m, 2H), 6.72 (tt, *J* = 7.2, 1.2 Hz, 1H), 6.63–6.49 (m, 2H), 6.04 (d, *J* = 1.2 Hz, 1H), 5.40 (d, *J* = 1.2 Hz, 1H), 3.09–2.93 (m, 2H), 2.60–2.52 (m, 1H), 2.47 (d, *J* = 1.0 Hz, 3H), 2.20 (s, 3H), 1.96 (dd, *J* = 7.7, 1.3 Hz, 1H).

^{13}C NMR (126 MHz, CD_2Cl_2) δ 171.0, 153.5, 152.0, 144.8, 141.8, 140.8, 129.3, 128.9, 128.9, 128.0, 127.8, 127.8, 126.7, 120.5, 119.6, 111.5, 75.7, 70.4, 57.9, 47.1, 46.6, 14.5, 14.3.

HRMS m/z (ESI): calculated for $C_{29}H_{28}N_3O$ $[M+H]^+$: 434.2227, found 434.2229.

The enantiomeric ratio was measured by HPLC analysis: 150 mm Chiralcel OJ-3R, 4.6 mm i.D., 3µm, MeCN/water = 60/40, 1 ml/min, 298K, λ = 220nm, t_R = 15.0 min (minor) and t_R = 17.2 min (major). er = 97.6:2.4.

$[\alpha]_D^{25}$ = –221 (*c* 2.0, $CHCl_3$).

(3-fluoroazetidin-1-yl)(1,2,3-triphenyl-2-azabicyclo[2.1.1]hexan-4-yl)methanone (3o): Prepared according to GP, purified *via* flash column chromatography (40% EtOAc in pentane) to afford **3o** in 81% yield as white solid.

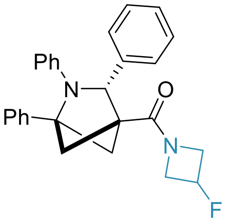

^{1}H NMR (501 MHz, CD_2Cl_2, 1:1 amide rotamers) δ 7.70 (dd, *J* = 7.5, 2.4 Hz, 2H), 7.55–7.36 (m, 7H), 7.34–7.27 (m, 1H), 6.97–6.89 (m, 2H), 6.74–6.67 (m, 1H), 6.57–6.51 (m, 2H), 5.17–4.74 (m, 1H), 4.82–4.75 (m, 1H), 4.26–4.11 (m, 1H), 4.04–3.71 (m, 2H), 2.89–2.62 (m, 3H), 2.46 (ddd, *J* = 12.4, 9.9, 7.2 Hz, 1H), 1.90 (ddd, *J* = 24.7, 7.2, 1.3 Hz, 1H).

^{13}C NMR (126 MHz, CD_2Cl_2) δ 170.8, 170.5, 151.8, 141.7, 141.6, 140.4, 140.3, 129.3, 129.1, 128.9, 128.9, 128.5, 128.4, 128.1, 128.0, 127.9, 126.5, 120.7, 120.6, 119.5, 119.5, 83.6, 83.1, 82.0, 81.4, 75.6, 75.6, 71.1, 71.0, 58.0, 58.0, 57.8, 57.8, 56.7, 56.7, 56.5, 56.5, 55.1, 55.0, 46.9, 46.8, 44.5.

^{19}F NMR (471 MHz, CD_2Cl_2) δ –181.1, –181.2.

HRMS m/z (EI): calculated for $C_{27}H_{25}N_2O_1F_1Na$ $[M+Na]^+$: 435.1846, found 435.1843.

The enantiomeric ratio was measured by HPLC analysis: 150 mm Chiralcel OJ-3R, 4.6 mm i.D., 3µm, MeCN/water = 50/50 1 ml/min, 298K, λ = 220 nm, t_R = 8.0 min (minor) and t_R = 8.9 min (major). er = 98.8:1.2.

$[\alpha]_D^{25}$ = –260 (*c* 2.0, $CHCl_3$).

1-(1,2,3-triphenyl-2-azabicyclo[2.1.1]hexane-4-carbonyl)piperidine-4-carbonitrile (3p): Prepared according to GP, purified *via* flash column chromatography (40% EtOAc in pentane) to afford **3p** in 58% yield as white solid.

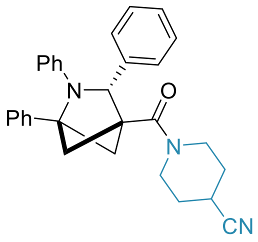

^{1}H NMR (501 MHz, CD_2Cl_2) δ 7.64 (d, J = 7.5 Hz, 2H), 7.56–7.51 (m, 2H), 7.46 (dt, J = 14.3, 7.5 Hz, 2H), 7.43–7.35 (m, 3H), 7.34–7.27 (m, 1H), 6.93 (dd, J = 8.7, 7.2 Hz, 2H), 6.71 (t, J = 7.3 Hz, 1H), 6.53 (d, J = 8.1 Hz, 2H), 4.77 (s, 1H), 4.04–3.93 (m, 0.5H), 3.73–3.55 (m, 1H), 3.27–2.94 (m, 2H), 2.90–2.78 (m, 1.5H), 2.78–2.71 (m, 1H), 2.69 (dd, J = 9.9, 7.1 Hz, 1H), 2.60 (dd, J = 9.9, 7.3 Hz, 1H), 2.03–1.97 (m, 1H), 1.95 – 1.69 (m, 2H), 1.69–1.52 (m, 1H), 1.34–1.02 (m, 1H).

^{13}C NMR (126 MHz, CD_2Cl_2) δ 168.8, 151.8, 141.5, 141.4, 140.3, 129.3, 129.1, 129.1, 128.9, 128.5, 128.4, 128.2, 128.2, 127.9, 126.5, 121.3, 120.7, 119.5, 76.0, 70.0, 55.9, 48.6, 45.6, 43.7, 43.2, 40.8, 40.3, 29.0, 28.9, 28.7, 26.8, 26.7.

HRMS m/z (ESI): calculated for $C_{30}H_{30}N_3O$ $[M+H]^+$: 448.2383, found 448.2387.

The enantiomeric ratio was measured by HPLC analysis: 150 mm Chiralcel IC-3R, 4.6 mm i.D., 3µm, MeCN/water = 60/40 1 ml/min, 298K, λ = 220 nm, t_R = 9.5 min (major) and t_R = 11.5 min (minor). er = 99.3:0.7.

$[\boldsymbol{\alpha}]_{\boldsymbol{D}}^{\mathbf{25}}$ = –253 (c 2.0, $CHCl_3$).

1-(1,2,3-triphenyl-2-azabicyclo[2.1.1]hexan-4-yl)pentan-1-one (3q): Prepared according to GP, purified *via* flash column chromatography (2 to 3% EtOAc in pentane) to afford **3q** in 80% yield as white solid.

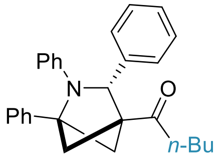

^{1}H NMR (501 MHz, CD_2Cl_2) δ 7.65–7.57 (m, 2H), 7.57–7.49 (m, 2H), 7.46–7.35 (m, 5H), 7.34–7.29 (m, 1H), 6.97–6.88 (m, 2H), 6.74–6.66 (m, 1H), 6.56–6.50 (m, 2H), 4.86 (s, 1H), 2.74 (d, J = 6.9 Hz, 1H), 2.66 (dd, J = 9.8, 6.9 Hz, 1H), 2.56 (dd, J = 9.9, 7.6 Hz, 1H), 2.50–2.41 (m, 1H), 2.21–2.12 (m, 1H), 1.90 (dd, J = 7.5, 1.4 Hz, 1H), 1.51–1.42 (m, 2H), 1.24 (h, J = 7.4 Hz, 2H), 0.85 (t, J = 7.4 Hz, 3H).

^{13}C NMR (151 MHz, CD_2Cl_2) δ 209.3, 151.8, 141.4, 140.4, 129.2, 128.8, 128.2, 128.0, 127.7, 126.4, 120.5, 119.2, 75.4, 70.2, 61.2, 46.1, 44.6, 40.2, 25.4, 22.6, 14.0.

HRMS m/z (ESI): calculated for $C_{28}H_{30}NO$ $[M+H]^+$: 396.2322, found 396.2323.

The enantiomeric ratio was measured by HPLC analysis: 150 mm Chiralcel OJ-3R, 4.6 mm i.D., 3µm, MeCN/water = 60/40, 1 ml/min, 298K, λ =220nm, t_R = 13.4 min (minor) and t_R = 17.3 min (major). er = 98.8:1.2.

$[\boldsymbol{\alpha}]_{\boldsymbol{D}}^{\mathbf{25}}$ = –253 (c 2.0, $CHCl_3$).

(4-(trifluoromethyl)phenyl)(1,2,3-triphenyl-2-azabicyclo[2.1.1]hexan-4-yl)methanone (3r): Prepared according to GP, purified *via* flash column chromatography (2% EtOAc in pentane) to afford **3r** in 60% yield as white solid.

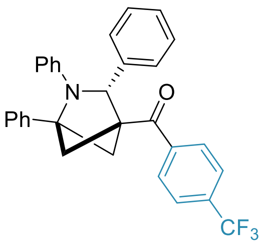

^{1}H NMR (501 MHz, CD_2Cl_2) δ 7.87–7.82 (m, 2H), 7.68 (d, *J* = 8.2 Hz, 2H), 7.58–7.53 (m, 2H), 7.46–7.38 (m, 4H), 7.35–7.30 (m, 4H), 7.00–6.93 (m, 2H), 6.75 (tt, *J* = 7.2, 1.1 Hz, 1H), 6.61–6.56 (m, 2H), 5.11 (s, 1H), 3.05 (d, *J* = 7.0 Hz, 1H), 2.99 (dd, *J* = 9.7, 7.0 Hz, 1H), 2.74 (dd, *J* = 9.7, 7.8 Hz, 1H), 2.05 (dd, *J* = 7.7, 1.4 Hz, 1H).

^{13}C NMR (126 MHz, CD_2Cl_2) δ 198.4, 151.7, 141.0, 140.2, 139.8, 134.6 (q, *J* = 32.5 Hz), 129.4, 129.4, 129.0, 129.0, 128.4, 128.2, 128.0, 126.5, 126.3 (q, *J* = 3.7 Hz), 124.2 (q, *J* = 257.5 Hz), 120.9, 119.5, 76.8, 70.7, 61.2, 47.7, 46.5.

^{19}F NMR (471 MHz, CD_2Cl_2) δ –63.51.

HRMS m/z (ESI): calculated for $C_{31}H_{24}NOF_3Na$ $[M+Na]^+$: 506.1702, found 506.1700.

The enantiomeric ratio was measured by HPLC analysis: 150 mm Chiralcel OJ-3R, 4.6 mm i.D., 3µm, MeCN/water = 70/30 1 ml/min, 298K, λ = 220nm, t_R = 6.7 min (minor) and t_R = 8.7 min (major). er = 98.1:1.9.

$[\alpha]_D^{25}$ = –214 (*c* 2.0, $CHCl_3$).

Thiophen-2-yl(1,2,3-triphenyl-2-azabicyclo[2.1.1]hexan-4-yl)methanone (3s): Prepared according to GP, purified *via* flash column chromatography (5% EtOAc in pentane) followed by prep-HPLC (Zorbax Eclipse Plus C18, 5µm 20mm i.D Acetonitril/Wasser = 80/20) to afford **3s** in 57% yield as white solid.

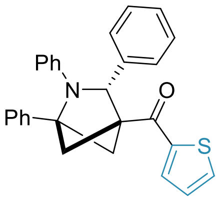

^{1}H NMR (501 MHz, CD_2Cl_2) δ 7.66 (dd, *J* = 5.0, 1.1 Hz, 1H), 7.60–7.48 (m, 5H), 7.42 (t, *J* = 7.7 Hz, 2H), 7.38–7.26 (m, 4H), 7.06 (dd, *J* = 5.0, 3.8 Hz, 1H), 7.01–6.93 (m, 2H), 6.74 (tt, *J* = 7.3, 1.2 Hz, 1H), 6.59 (dd, *J* = 8.8, 1.1 Hz, 2H), 5.08 (s, 1H), 3.02 (d, *J* = 7.1 Hz, 1H), 2.96 (dd, *J* = 9.7, 7.0 Hz, 1H), 2.72 (dd, *J* = 9.7, 7.6 Hz, 1H), 2.08 (dd, *J* = 7.6, 1.4 Hz, 1H).

^{13}C NMR (126 MHz, CD_2Cl_2) δ 191.5, 151.9, 143.7, 141.1, 140.4, 134.5, 133.0, 129.4, 129.0, 128.9, 128.8, 128.2, 127.9, 126.5, 120.7, 119.4, 77.1, 70.7, 60.8, 47.5, 46.1.

HRMS m/z (ESI): calculated for $C_{28}H_{23}NOSNa$ $[M+Na]^+$: 444.1393, found 444.1390.

The enantiomeric ratio was measured by HPLC analysis: 150 mm Chiralcel OJ-3R, 4.6 mm i.D., 3µm, MeCN/water = 70/30 1 ml/min, 298K, λ = 220nm, t_R = 7.1 min (minor) and t_R = 8.4 min (major). er = 98.5:1.5.

$[\alpha]_D^{25}$ = –218 (*c* 2.0, $CHCl_3$).

Methyl 1,2,3-triphenyl-2-azabicyclo[2.1.1]hexane-4-carboxylate (3t): Prepared according to GP, *via* flash column chromatography (5% EtOAc in pentane) followed by prep-HPLC (Zorbax Eclipse Plus C18, 5µm 20mm i.D Acetonitril/Wasser = 80/20) to afford **3t** in 76% yield as white solid.

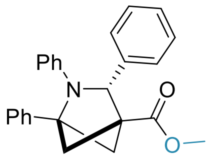

^{1}H NMR (501 MHz, CD_2Cl_2) δ 7.72–7.66 (m, 2H), 7.56–7.50 (m, 2H), 7.48–7.42 (m, 2H), 7.42–7.34 (m, 3H), 7.34–7.28 (m, 1H), 6.97–6.90 (m, 2H), 6.71 (tt, *J* = 7.3, 1.2 Hz, 1H), 6.56–6.49 (m, 2H), 4.88 (s, 1H), 3.68 (s, 3H), 2.84 (d, *J* = 6.9 Hz, 1H), 2.65 (dd, *J* = 9.9, 6.9 Hz, 1H), 2.47 (dd, *J* = 9.9, 7.7 Hz, 1H), 1.97 (dd, *J* = 7.7, 1.5 Hz, 1H).

^{13}C NMR (126 MHz, CD_2Cl_2) δ 171.2, 151.9, 141.6, 140.4, 129.3, 128.9, 128.3, 128.2, 127.9, 126.5, 120.6, 119.4, 75.0, 70.9, 54.9, 52.1, 46.1, 45.0.

HRMS m/z (ESI): calculated for $C_{25}H_{23}NO_2Na$ $[M+Na]^+$: 392.1621, found 392.1625.

The enantiomeric ratio was measured by HPLC analysis: 150 mm Chiralcel OJ-3R, 4.6 mm i.D., 3µm, MeCN/water = 60/40, 1 ml/min, 298K, λ = 220 nm, t_R = 8.6 min (minor) and t_R = 9.7 min (major). er = 96.8:3.2.

$[\boldsymbol{\alpha}]_{\boldsymbol{D}}^{\mathbf{25}}$ = –225 (*c* 2.0, $CHCl_3$).

7 Crystallographic Data of 3c

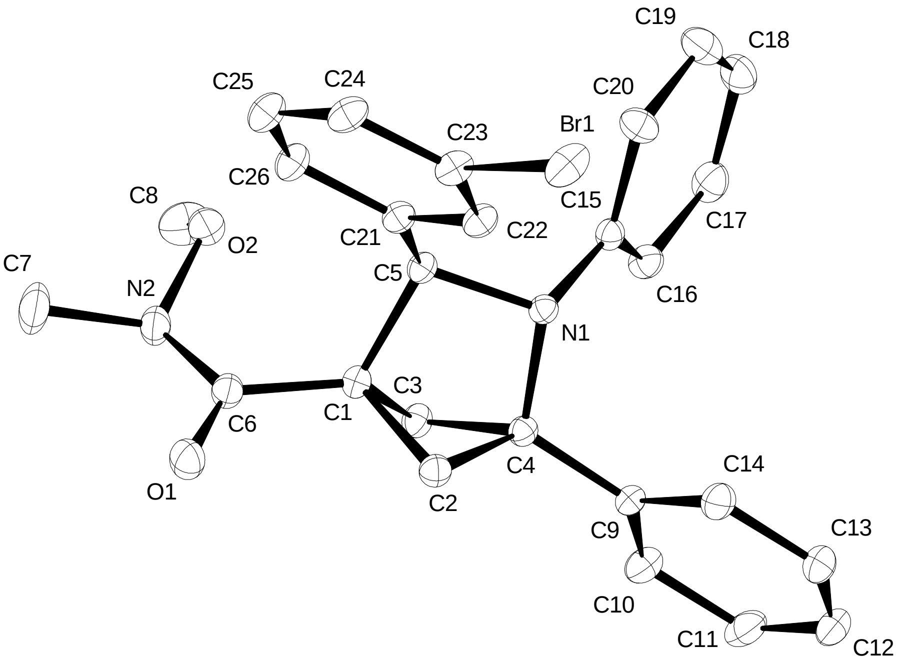

Figure SI- 5: X-Ray structure of compound **3c** (H atoms omitted).

Table SI-2: Crystal data and structure refinement.

Identification code	16146	
Empirical formula	$C_{26} H_{25} Br N_2 O_2$	
Color	colourless	
Formula weight	477.39 g · mol^{-1}	
Temperature	100(2) K	
Wavelength	0.71073 Å	
Crystal system	MONOCLINIC	
Space group	**$P2_1$, (no. 4)**	
Unit cell dimensions	a = 10.0608(13) Å	α= 90°.
	b = 10.3532(13) Å	β= 93.052(4)°.
	c = 10.7405(15) Å	γ = 90°.

Volume	1117.2(3) $Å^3$
Z	2
Density (calculated)	1.419 $Mg \cdot m^{-3}$
Absorption coefficient	1.865 mm^{-1}
F(000)	492 e
Crystal size	0.102 x 0.053 x 0.041 mm^3
θ range for data collection	1.899 to 32.633°.
Index ranges	$-15 \leq h \leq 15$, $-15 \leq k \leq 15$, $-16 \leq l \leq 16$
Reflections collected	377522
Independent reflections	8157 [R_{int} = 0.0703]
Reflections with I>2σ(I)	7841
Completeness to θ = 25.242°	100.0 %
Absorption correction	Gaussian
Max. and min. transmission	0.93 and 0.87
Refinement method	Full-matrix least-squares on F^2
Data / restraints / parameters	8157 / 1 / 287
Goodness-of-fit on F^2	1.059
Final R indices [I>2σ(I)]	R_1 = 0.0188 wR^2 = 0.0504
R indices (all data)	R_1 = 0.0202 wR^2 = 0.0509
Absolute structure parameter	0.027(4)
Largest diff. peak and hole	0.3 and -0.4 e · $Å^{-3}$

Table SI-3: Bond lengths [Å] and angles [°].

Br(1)-C(23)	1.8959(14)	O(1)-C(6)	1.2239(17)
O(2)-N(2)	1.4123(16)	O(2)-C(8)	1.4291(19)
N(1)-C(4)	1.4945(16)	N(1)-C(5)	1.5016(15)
N(1)-C(15)	1.4239(16)	N(2)-C(6)	1.3739(19)
N(2)-C(7)	1.457(2)	C(1)-C(2)	1.5451(18)
C(1)-C(3)	1.5613(18)	C(1)-C(4)	2.0386(18)
C(1)-C(5)	1.5558(17)	C(1)-C(6)	1.5124(18)
C(2)-C(4)	1.5522(17)	C(3)-C(4)	1.5635(18)
C(4)-C(9)	1.4962(18)	C(5)-H(5)	0.949(19)
C(5)-C(21)	1.513(2)	C(9)-C(10)	1.3908(19)
C(9)-C(14)	1.3994(19)	C(10)-C(11)	1.400(2)
C(11)-C(12)	1.383(2)	C(12)-C(13)	1.389(3)
C(13)-C(14)	1.3944(18)	C(15)-C(16)	1.4005(19)
C(15)-C(20)	1.4021(18)	C(16)-C(17)	1.3959(19)
C(17)-C(18)	1.389(2)	C(18)-C(19)	1.394(2)
C(19)-C(20)	1.392(2)	C(21)-C(22)	1.3935(19)
C(21)-C(26)	1.4010(18)	C(22)-C(23)	1.3941(19)
C(23)-C(24)	1.393(2)	C(24)-C(25)	1.391(2)
C(25)-C(26)	1.393(2)		
N(2)-O(2)-C(8)	110.50(12)	C(4)-N(1)-C(5)	101.58(9)
C(15)-N(1)-C(4)	119.03(11)	C(15)-N(1)-C(5)	113.29(10)
O(2)-N(2)-C(7)	112.97(12)	C(6)-N(2)-O(2)	115.56(11)
C(6)-N(2)-C(7)	120.87(14)	C(2)-C(1)-C(3)	86.67(9)
C(2)-C(1)-C(4)	48.99(7)	C(2)-C(1)-C(5)	100.60(10)
C(3)-C(1)-C(4)	49.32(7)	C(5)-C(1)-C(3)	101.34(10)
C(5)-C(1)-C(4)	79.21(7)	C(6)-C(1)-C(2)	117.66(11)
C(6)-C(1)-C(3)	123.21(11)	C(6)-C(1)-C(4)	159.81(10)
C(6)-C(1)-C(5)	120.55(10)	C(1)-C(2)-C(4)	82.32(9)
C(1)-C(3)-C(4)	81.45(9)	N(1)-C(4)-C(1)	80.46(8)
N(1)-C(4)-C(2)	99.89(10)	N(1)-C(4)-C(3)	103.66(10)
N(1)-C(4)-C(9)	115.07(10)	C(2)-C(4)-C(1)	48.69(7)
C(2)-C(4)-C(3)	86.35(9)	C(3)-C(4)-C(1)	49.23(7)
C(9)-C(4)-C(1)	163.77(10)	C(9)-C(4)-C(2)	120.19(11)

C(9)-C(4)-C(3)	125.87(11)	N(1)-C(5)-C(1)	98.66(9)
N(1)-C(5)-H(5)	108.6(11)	N(1)-C(5)-C(21)	115.29(13)
C(1)-C(5)-H(5)	106.6(11)	C(21)-C(5)-C(1)	115.04(11)
C(21)-C(5)-H(5)	111.6(11)	O(1)-C(6)-N(2)	120.67(12)
O(1)-C(6)-C(1)	121.09(12)	N(2)-C(6)-C(1)	118.13(12)
C(10)-C(9)-C(4)	121.44(12)	C(10)-C(9)-C(14)	119.42(12)
C(14)-C(9)-C(4)	119.14(12)	C(9)-C(10)-C(11)	120.13(14)
C(12)-C(11)-C(10)	120.25(15)	C(11)-C(12)-C(13)	119.88(13)
C(12)-C(13)-C(14)	120.28(17)	C(13)-C(14)-C(9)	120.01(15)
C(16)-C(15)-N(1)	124.01(12)	C(16)-C(15)-C(20)	118.65(12)
C(20)-C(15)-N(1)	117.32(12)	C(17)-C(16)-C(15)	120.11(13)
C(18)-C(17)-C(16)	121.06(14)	C(17)-C(18)-C(19)	118.96(13)
C(20)-C(19)-C(18)	120.51(14)	C(19)-C(20)-C(15)	120.69(14)
C(22)-C(21)-C(5)	122.51(11)	C(22)-C(21)-C(26)	119.35(12)
C(26)-C(21)-C(5)	118.10(12)	C(21)-C(22)-C(23)	119.26(12)
C(22)-C(23)-Br(1)	119.57(11)	C(24)-C(23)-Br(1)	118.65(10)
C(24)-C(23)-C(22)	121.76(13)	C(25)-C(24)-C(23)	118.65(13)
C(24)-C(25)-C(26)	120.27(13)	C(25)-C(26)-C(21)	120.68(13)

8 NMR Spectra

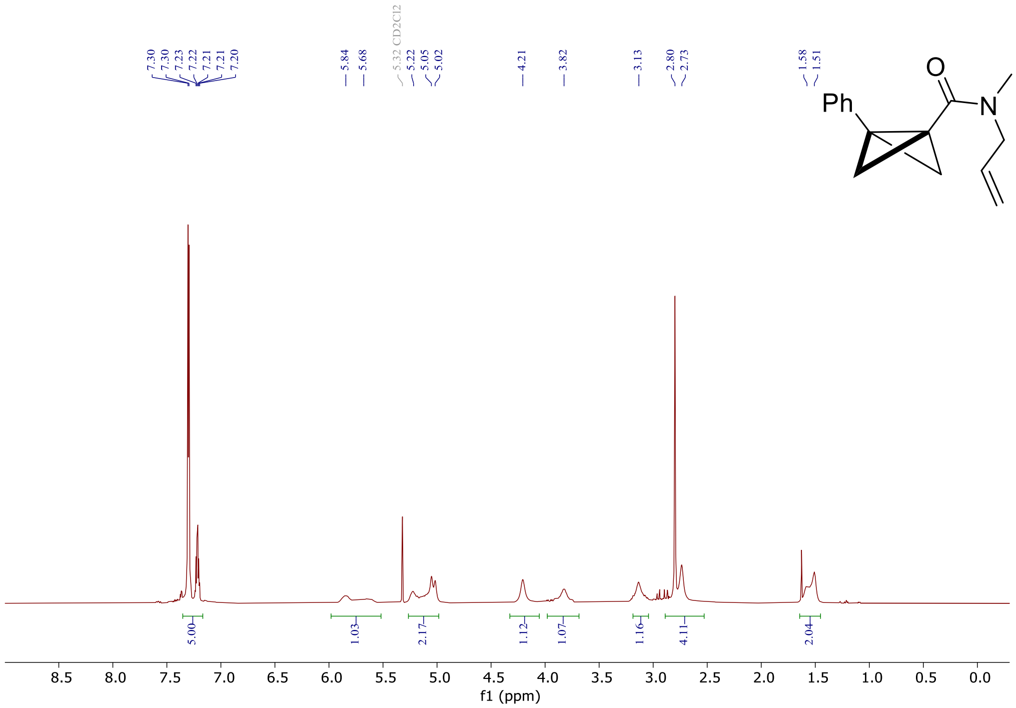

^{1}H-NMR spectrum of compound **1b**.

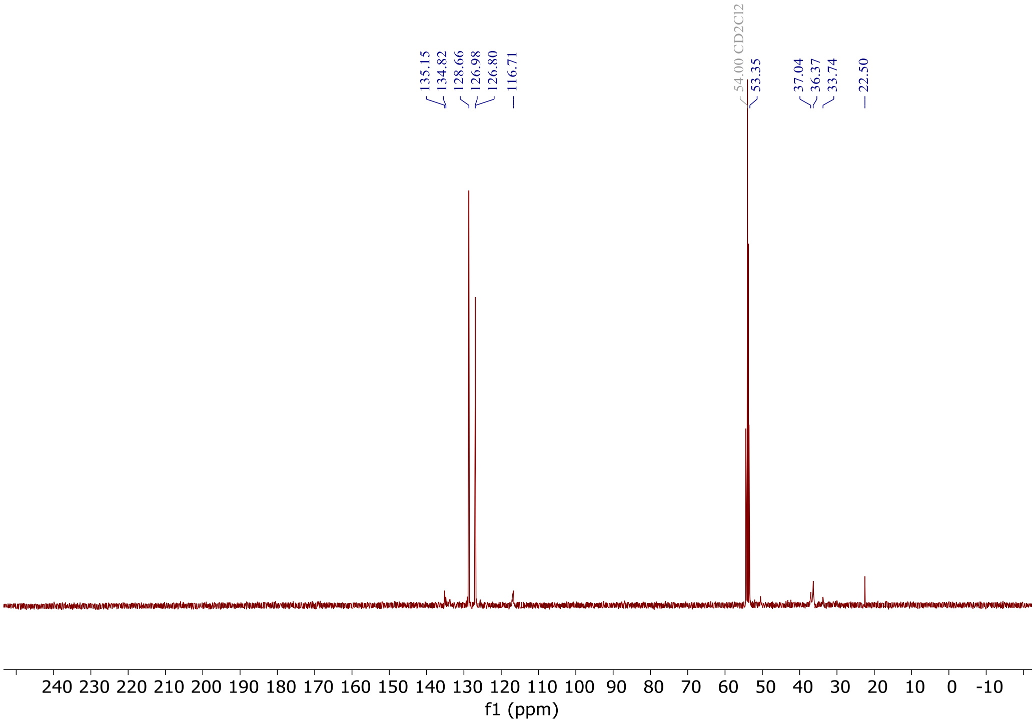

^{13}C-NMR spectrum of compound **1b**.

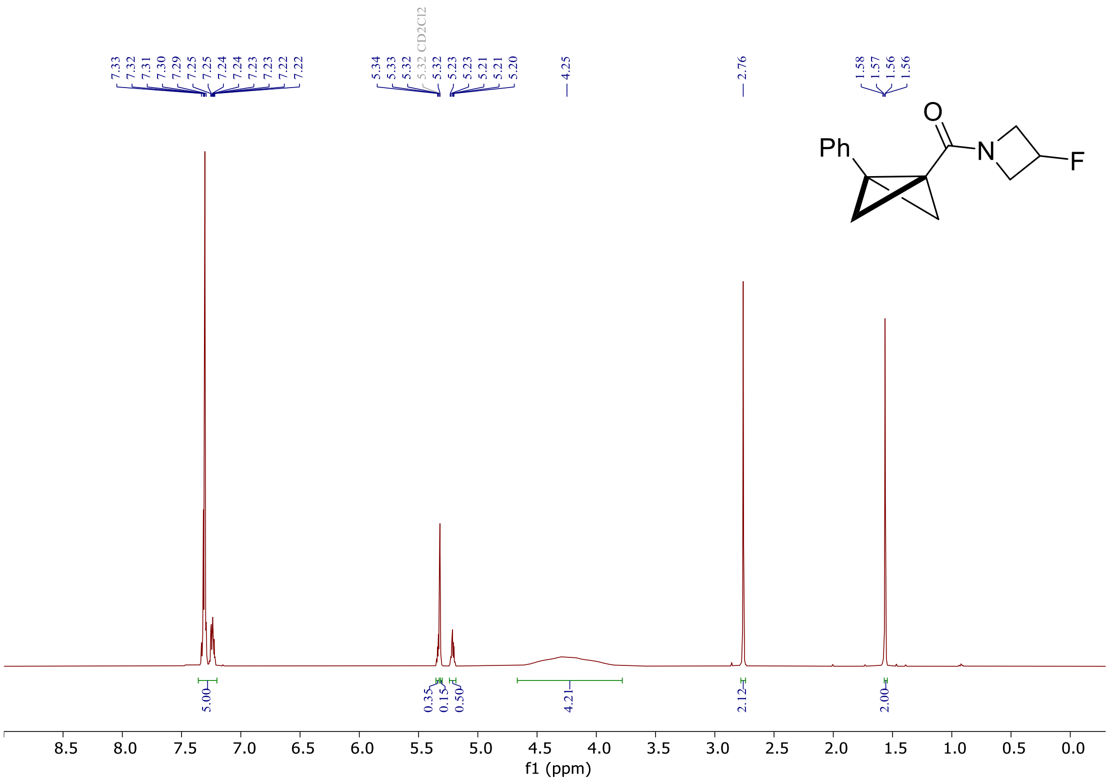

[1]**H-NMR** spectrum of compound **1e**.

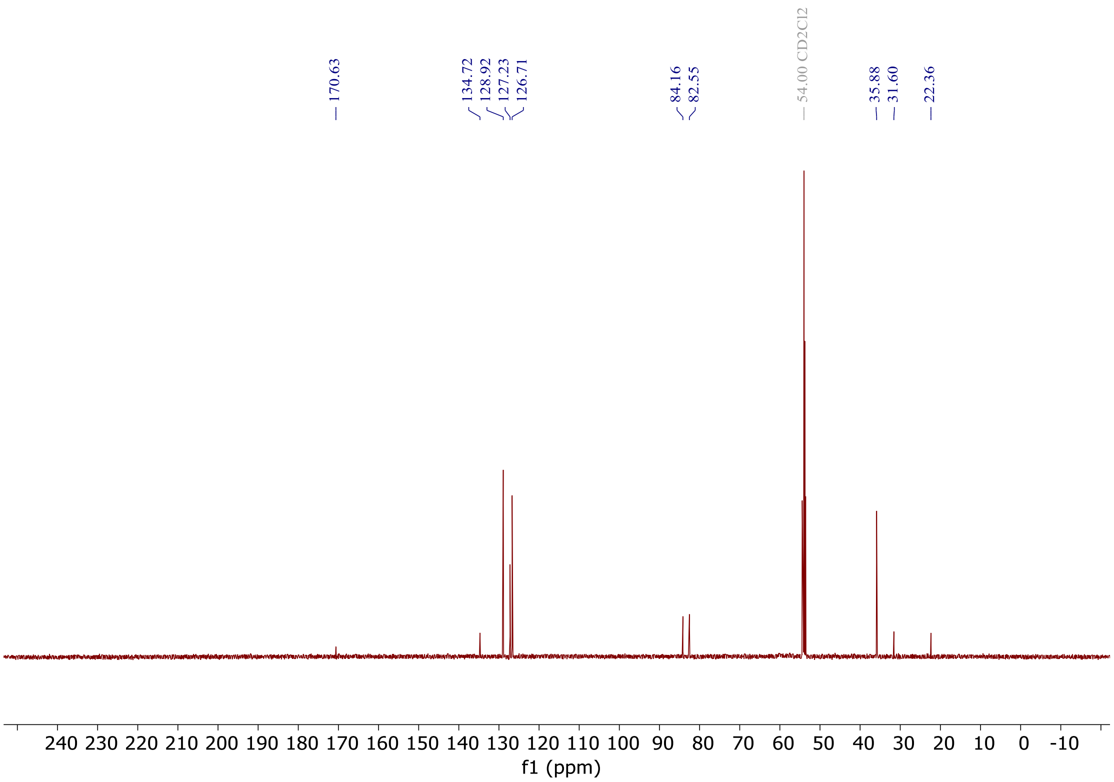

[13]**C-NMR** spectrum of compound **1e**.

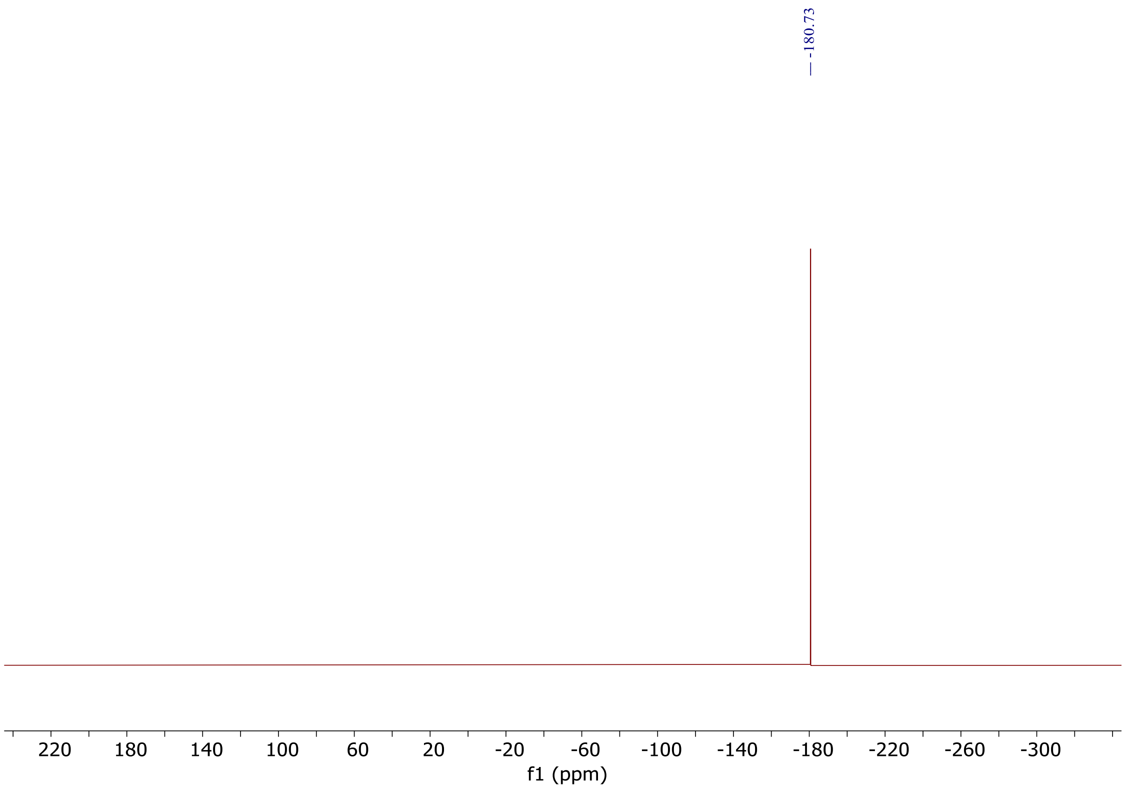

^{19}F-NMR spectrum of compound **1e**.

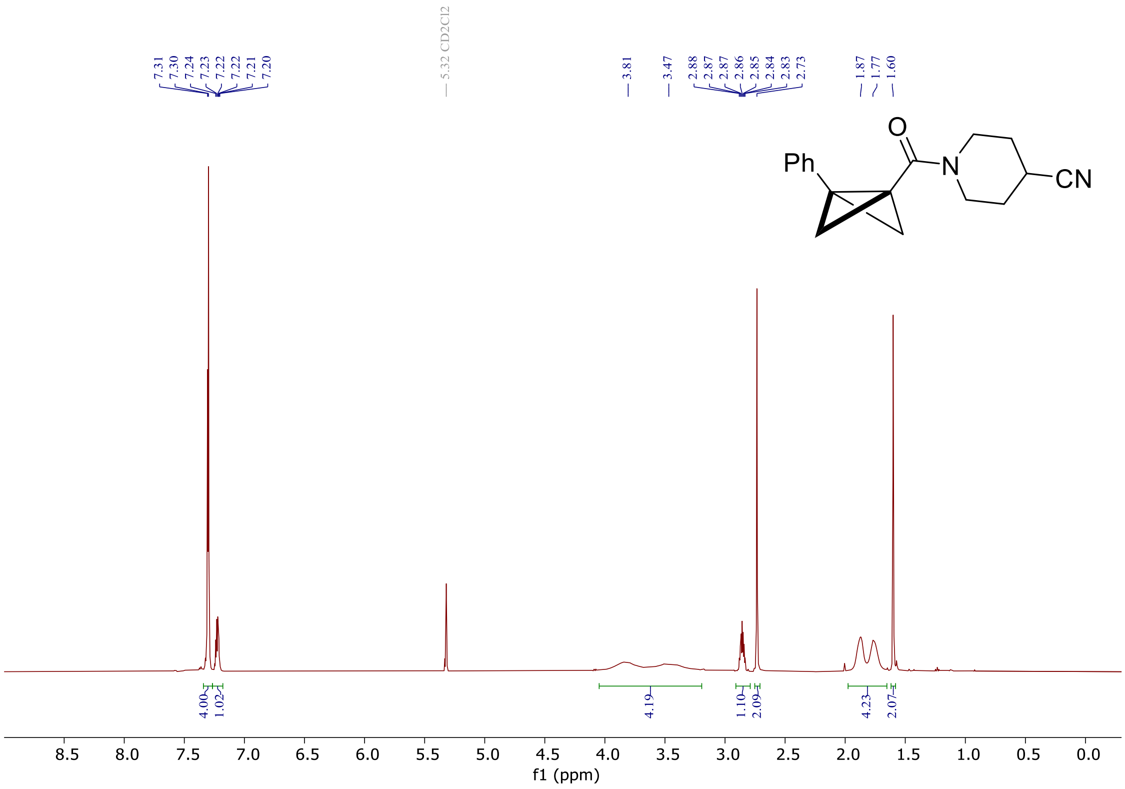

^{1}H-NMR spectrum of compound **1f**.

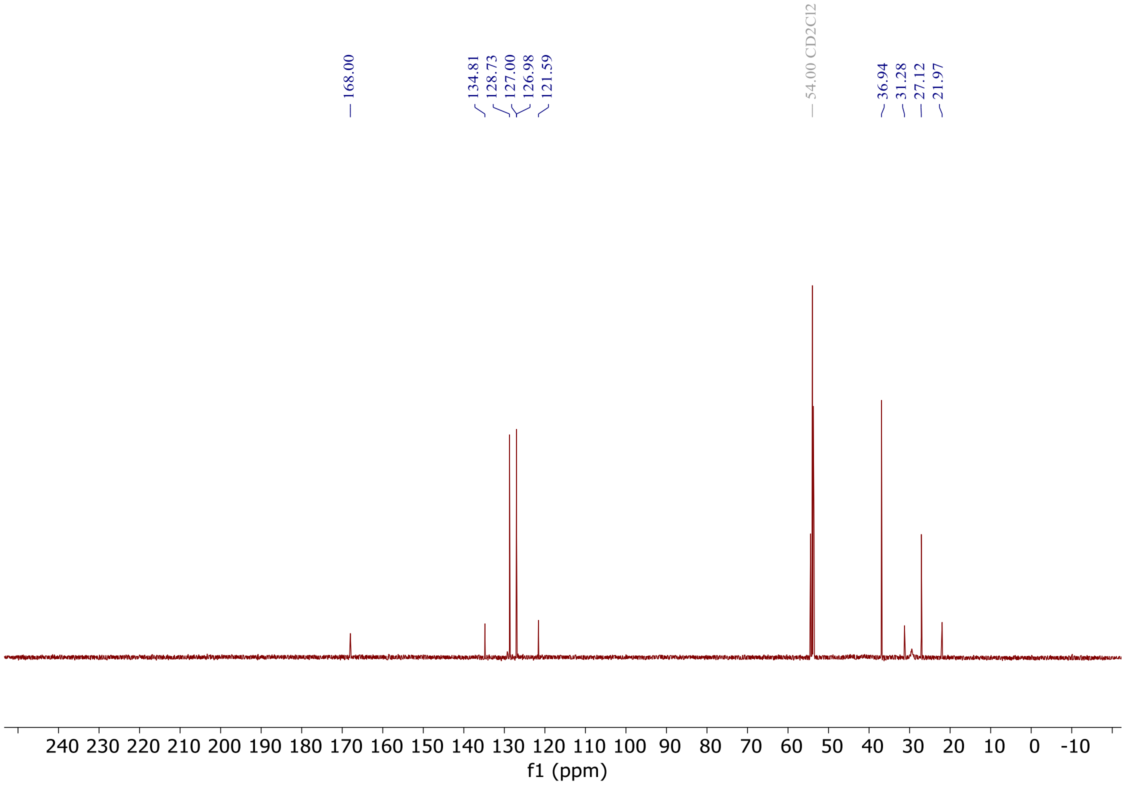

^{13}C-NMR spectrum of compound **1f**.

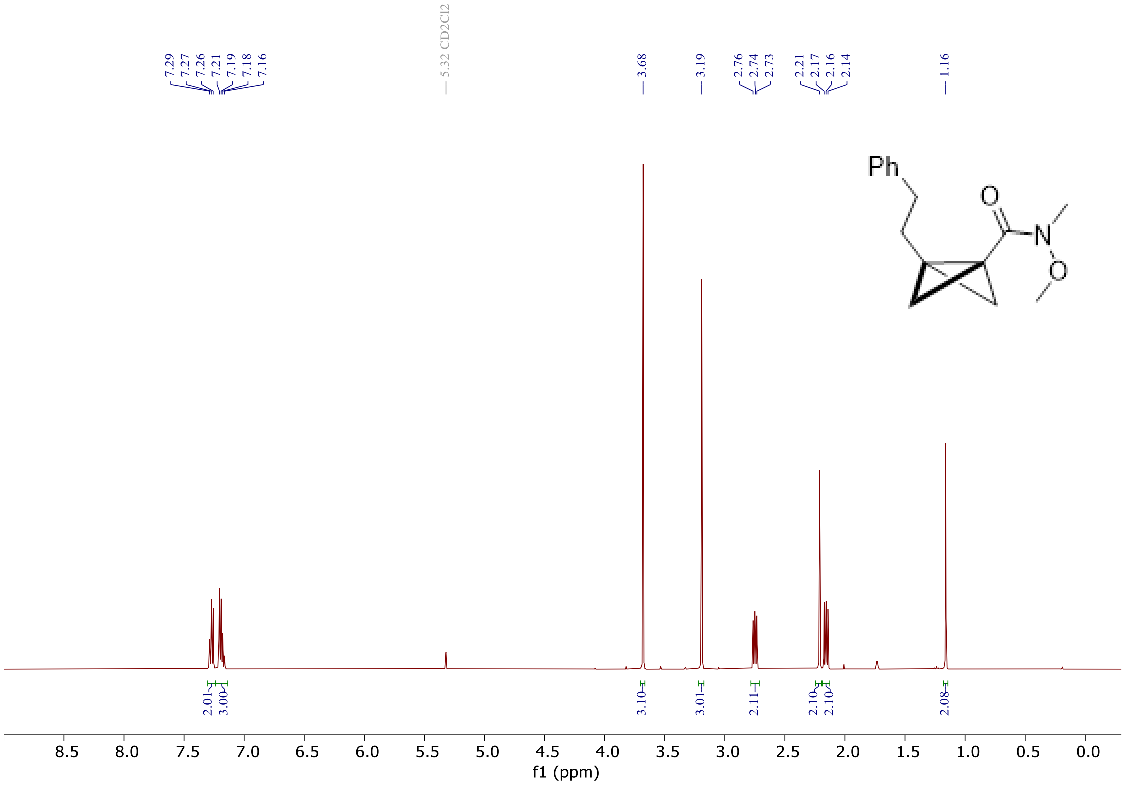

^{1}H-NMR spectrum of compound **1k**.

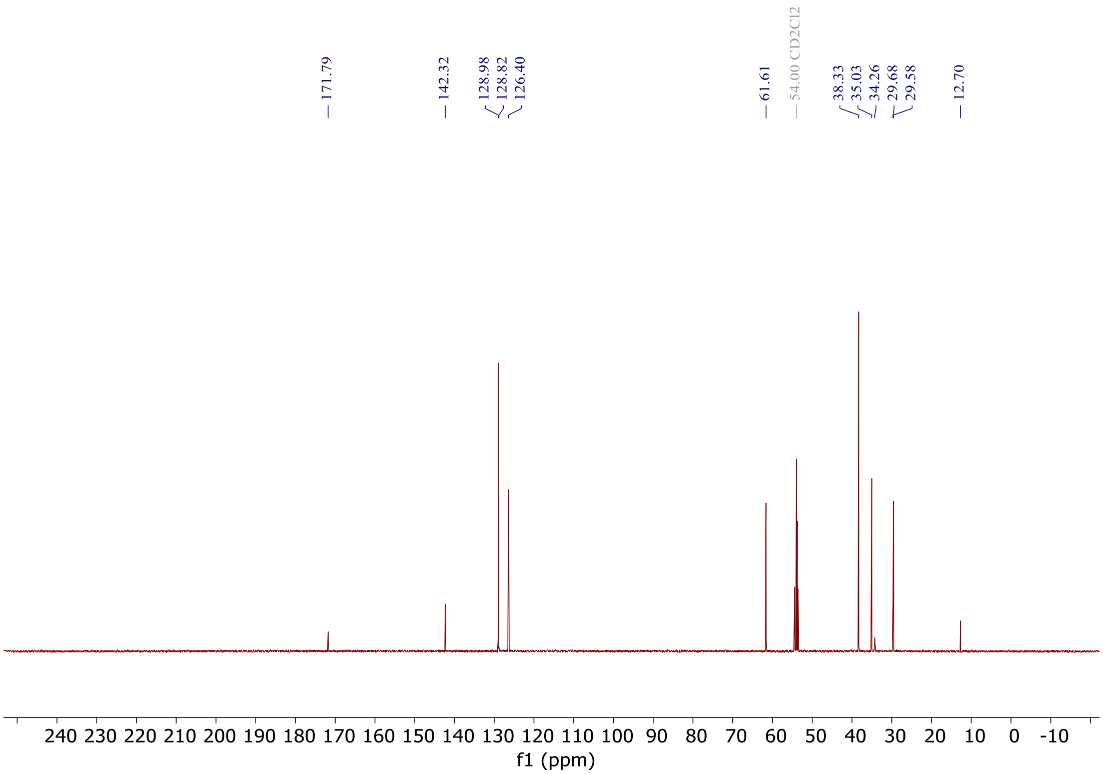

^{13}**C-NMR** spectrum of compound **1k**.

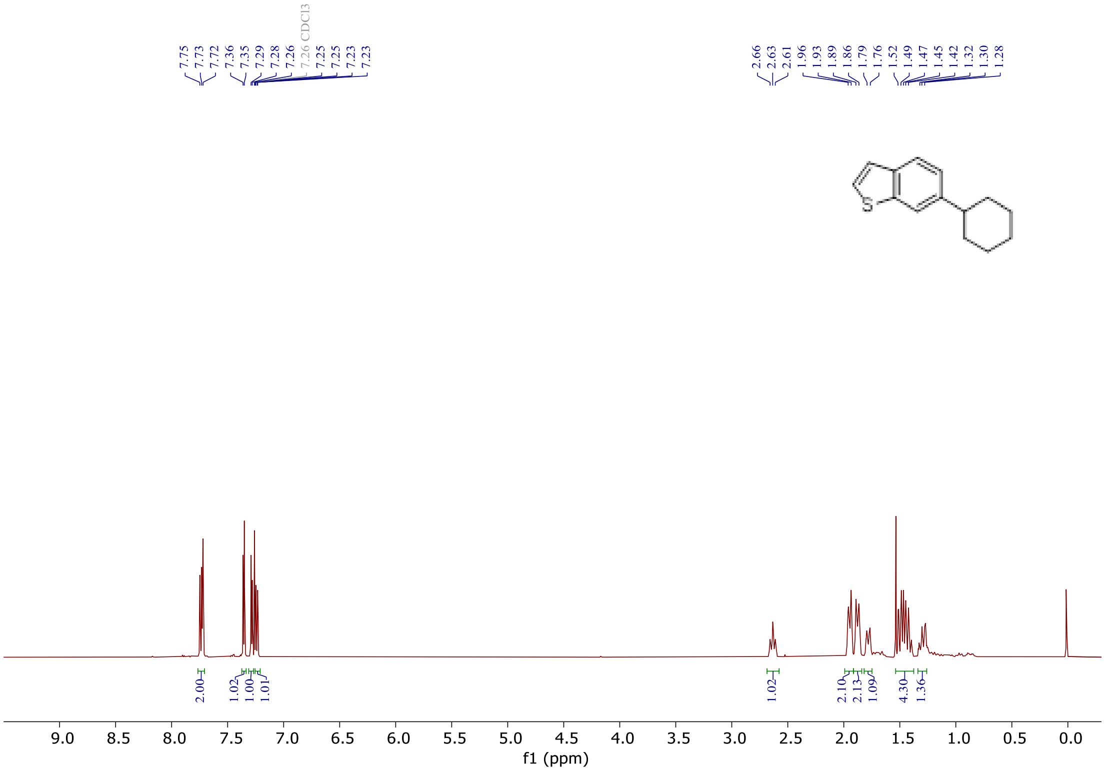

^{1}**H-NMR** spectrum of compound **S2**.

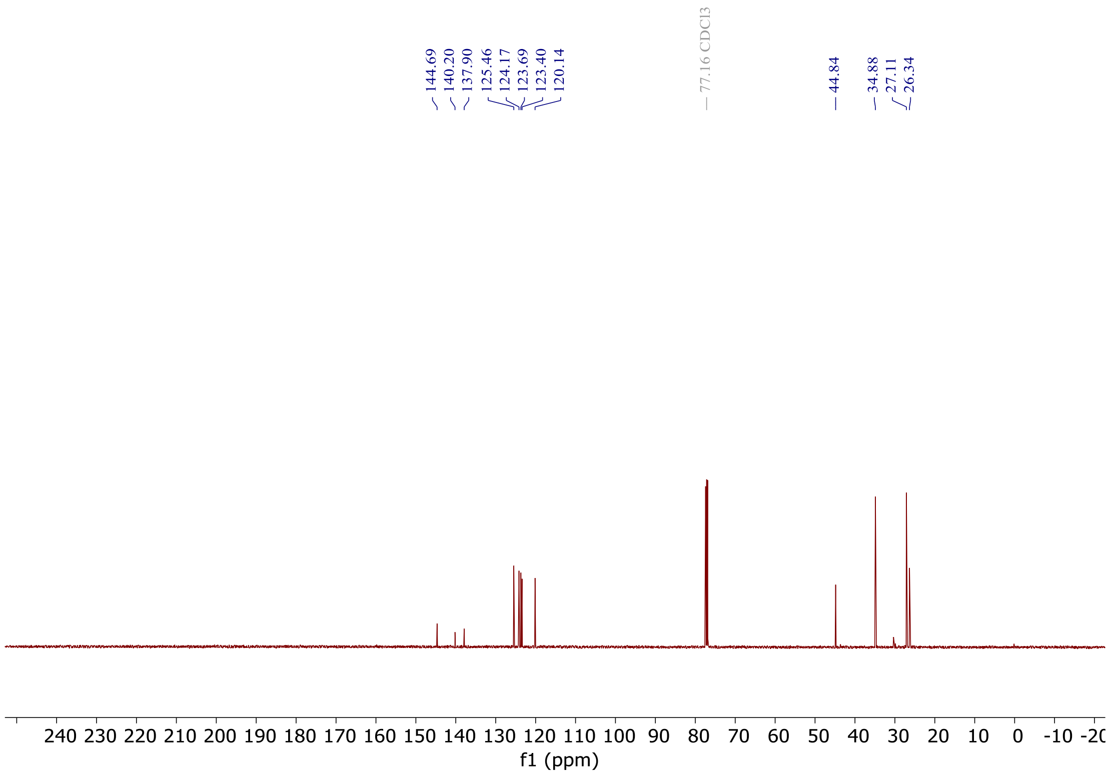

^{13}C-NMR spectrum of compound **S2**.

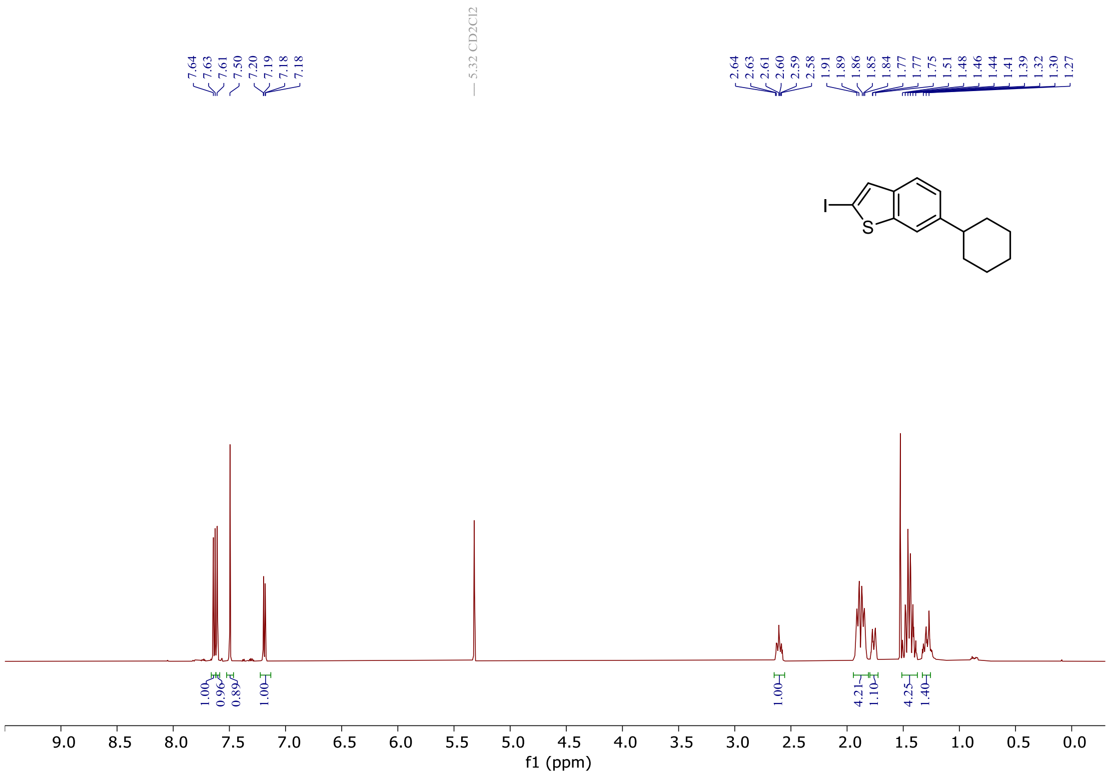

^{1}H-NMR spectrum of compound **S3**.

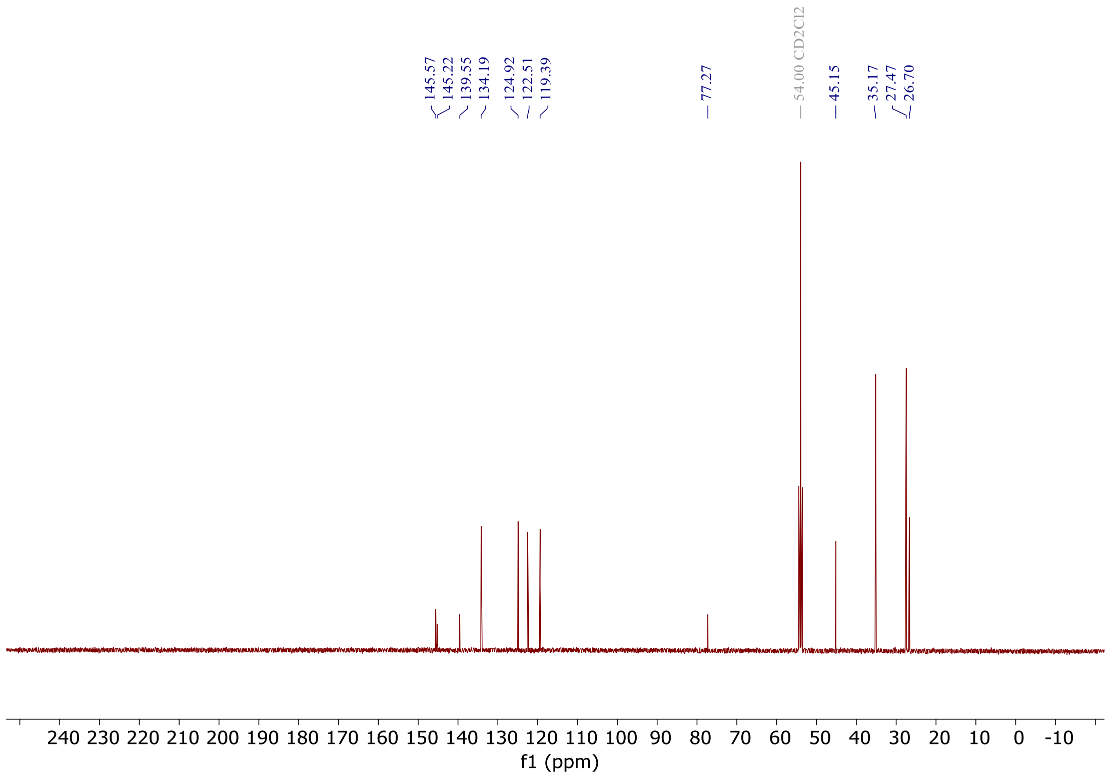

^{13}C-NMR spectrum of compound **S3**.

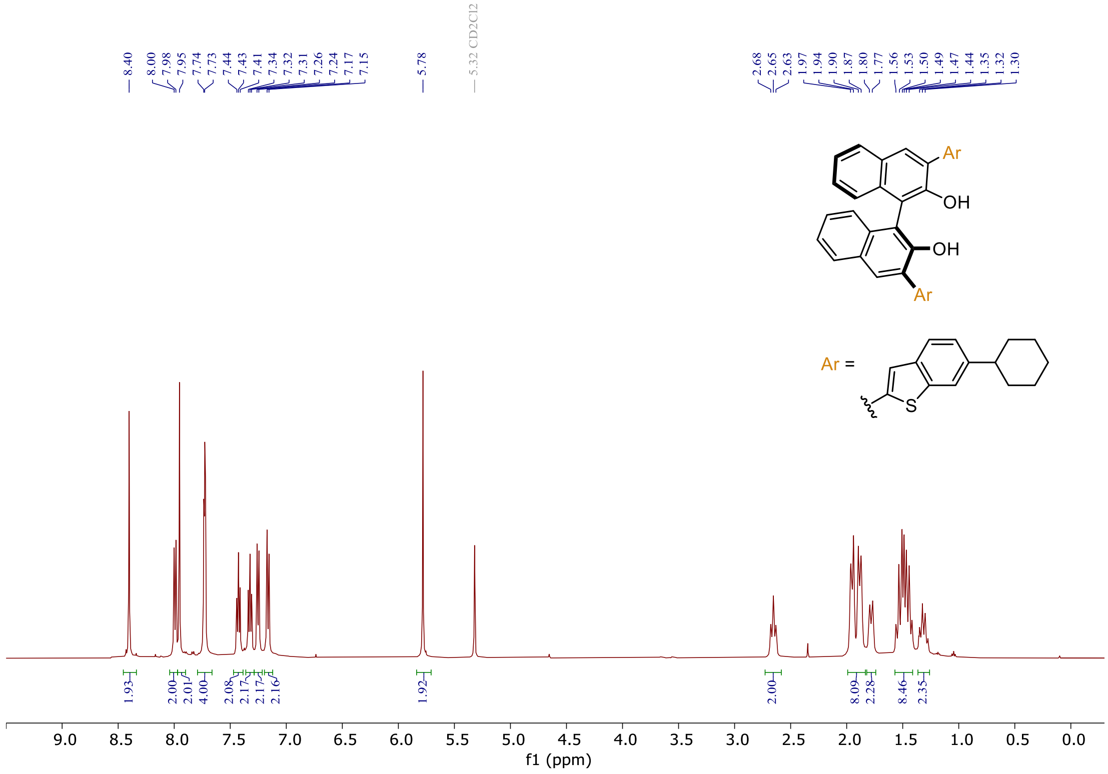

^{1}H-NMR spectrum of compound **S4**.

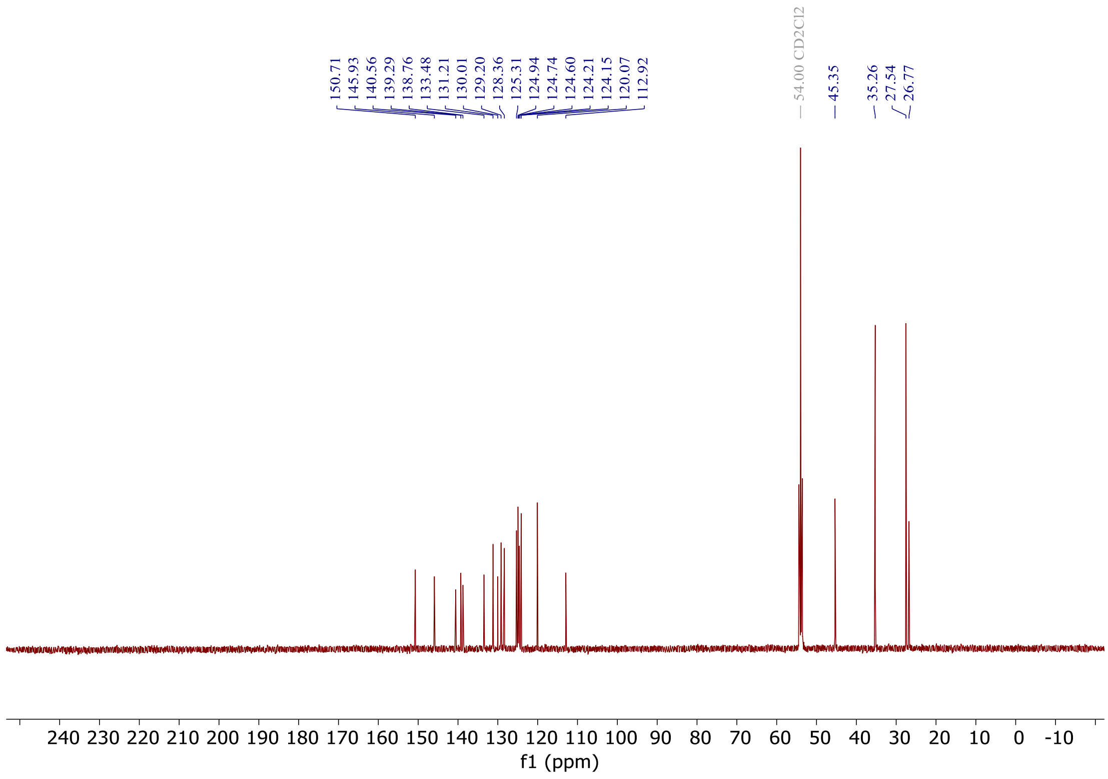

^{13}C-NMR spectrum of compound **S4**.

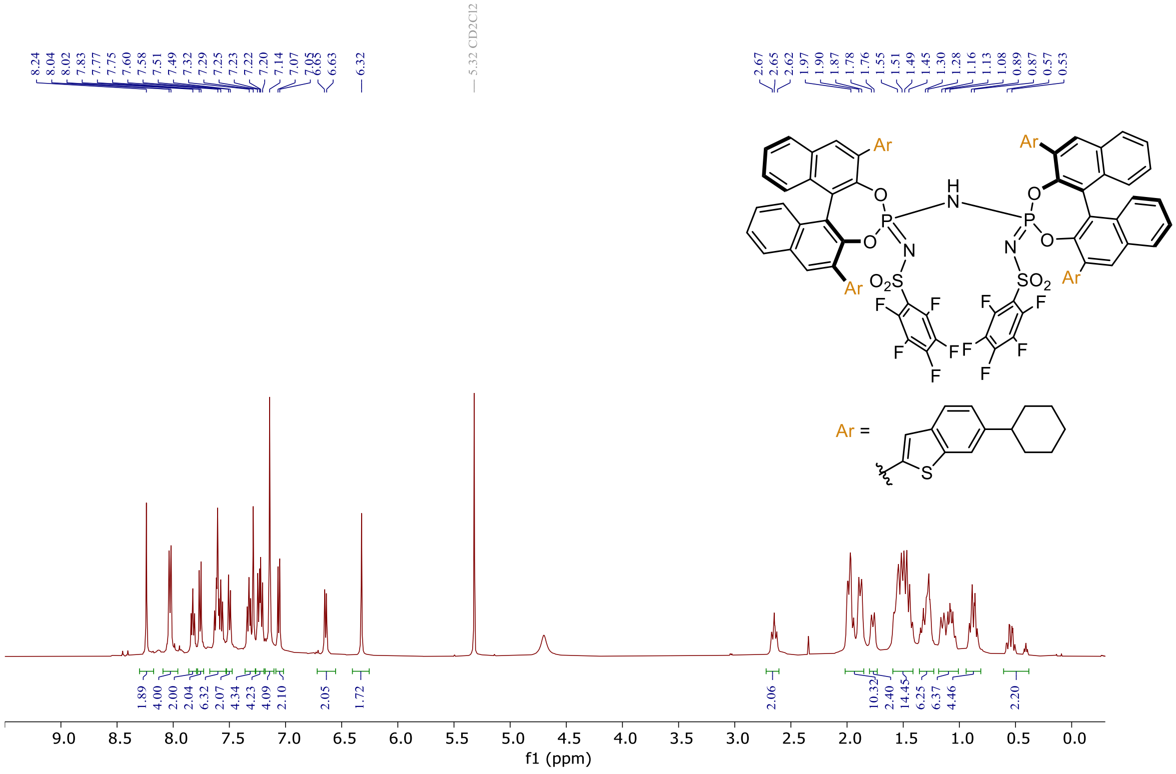

^{1}H-NMR spectrum of compound **7b.**

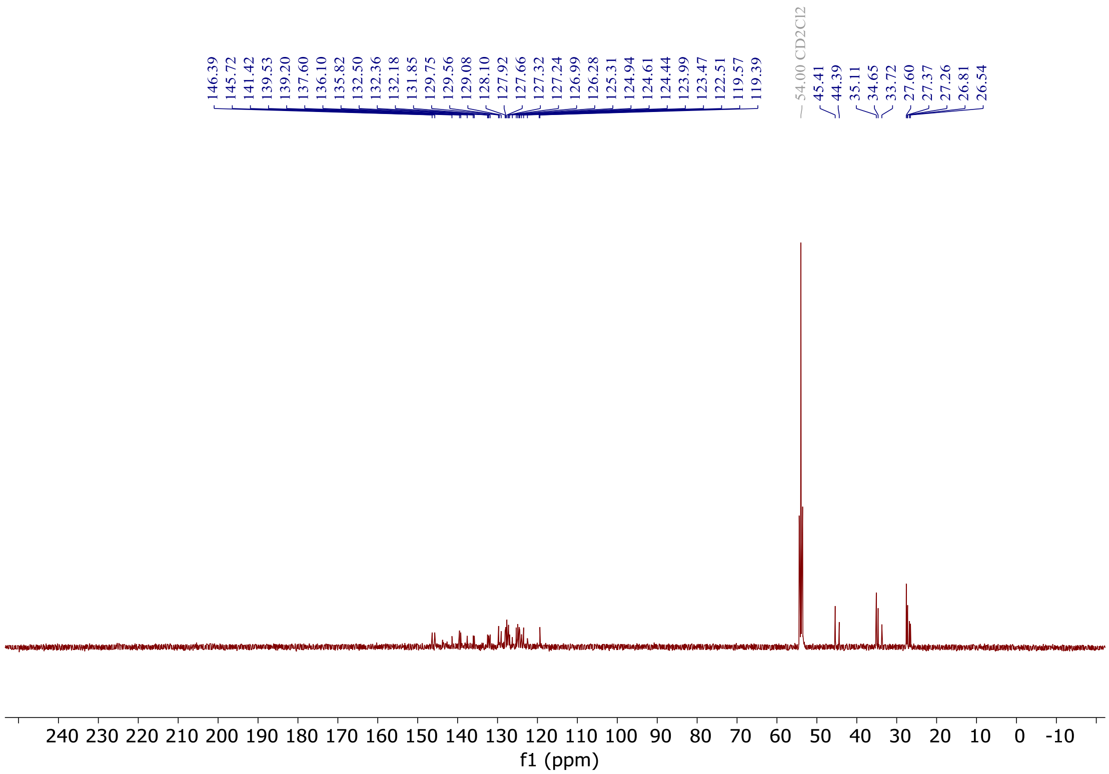

^{13}C-NMR spectrum of compound **7b**.

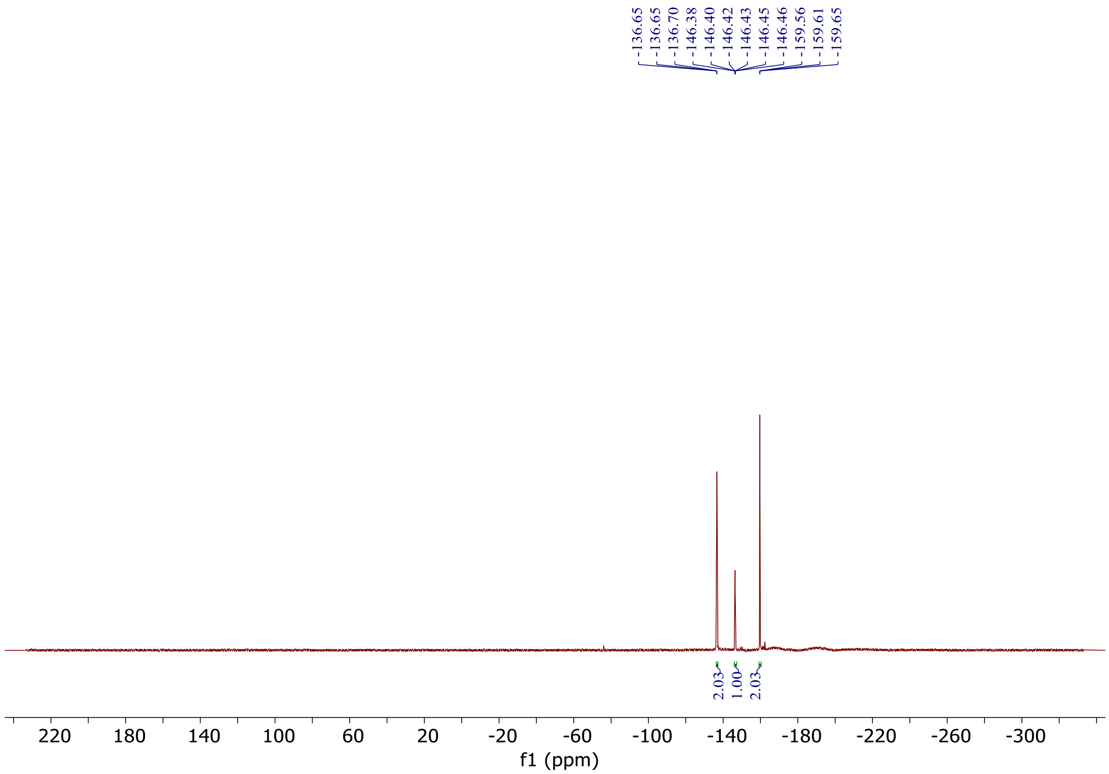

^{19}F-NMR spectrum of compound **7b**.

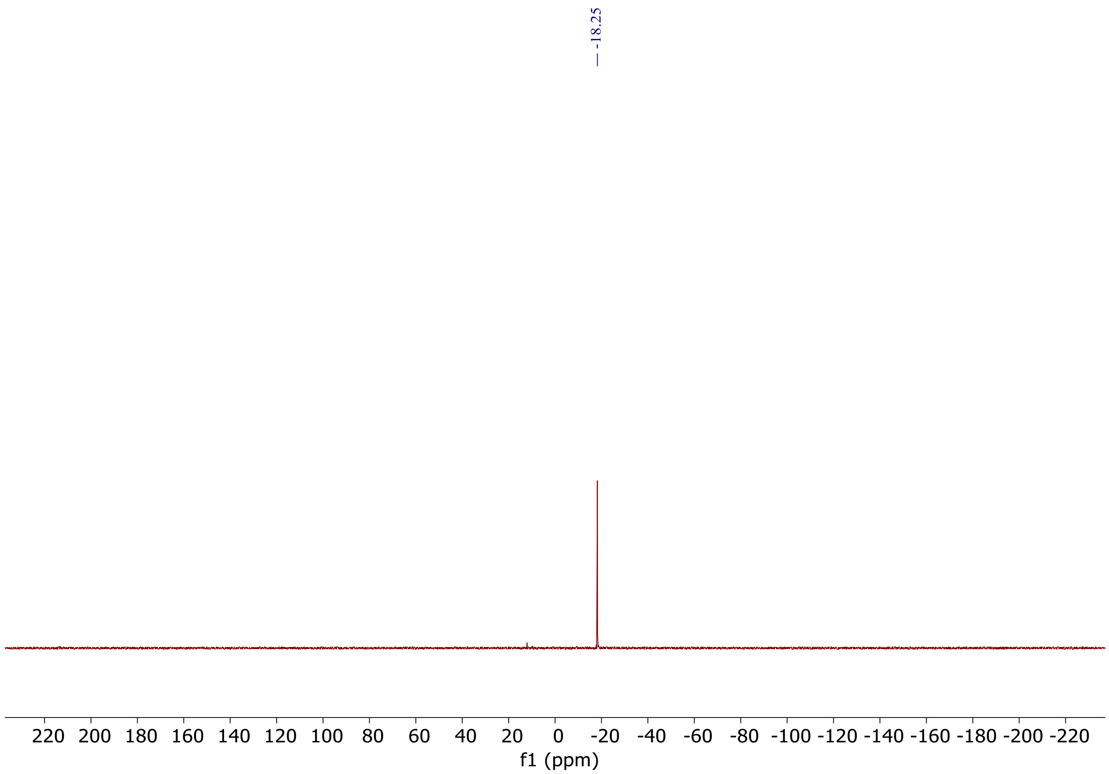

^{31}P-NMR spectrum of compound **7b**.

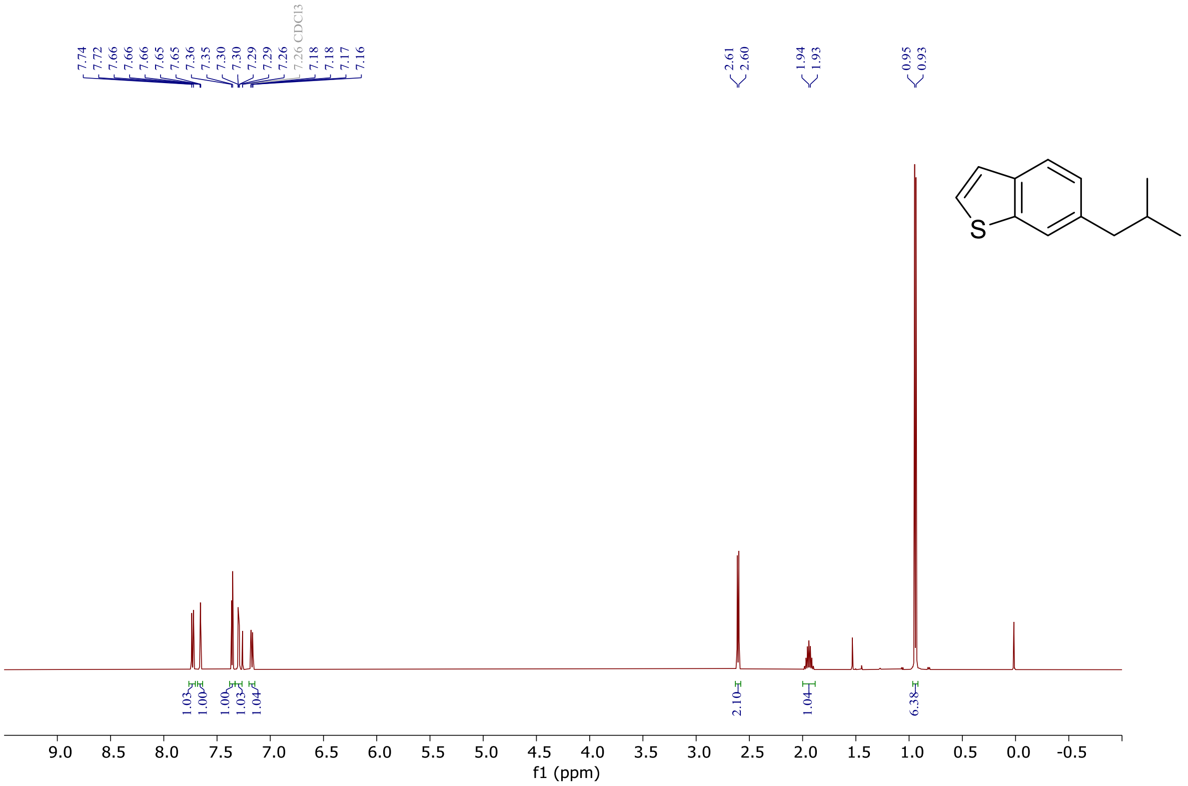

^{1}H-NMR spectrum of compound **S5.**

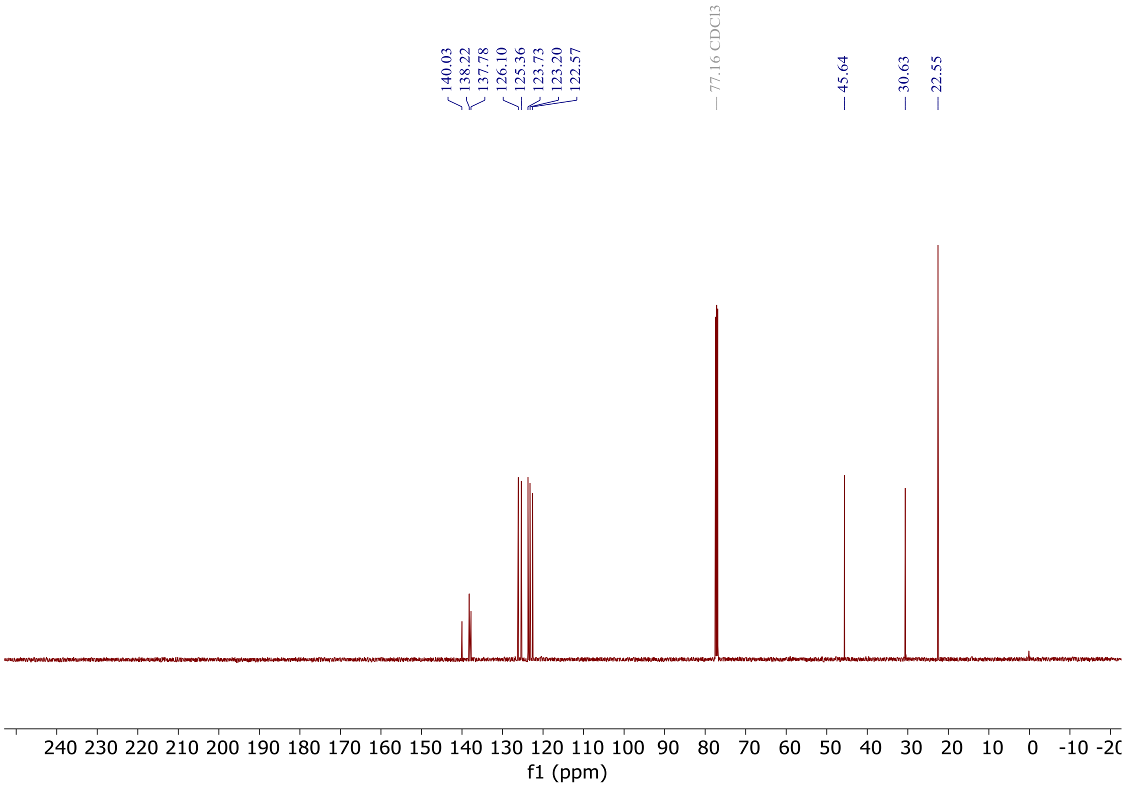

^{13}C-NMR spectrum of compound **S5**.

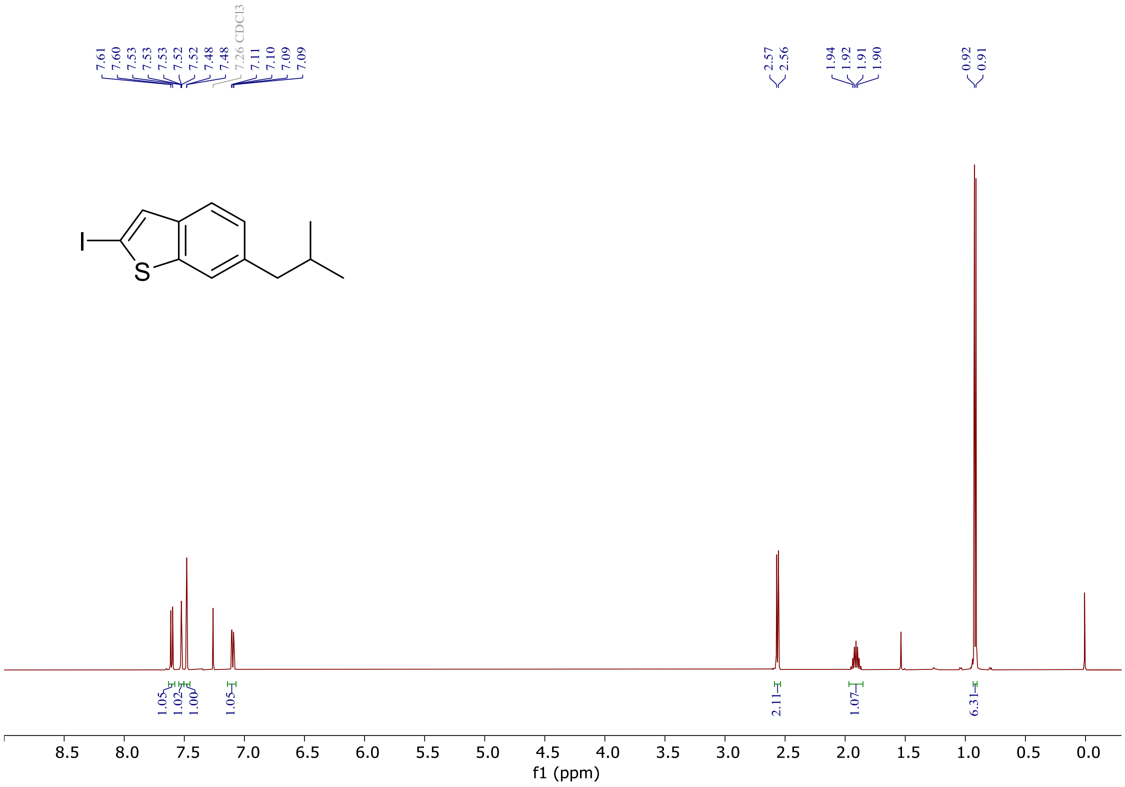

^{1}H-NMR spectrum of compound **S6.**

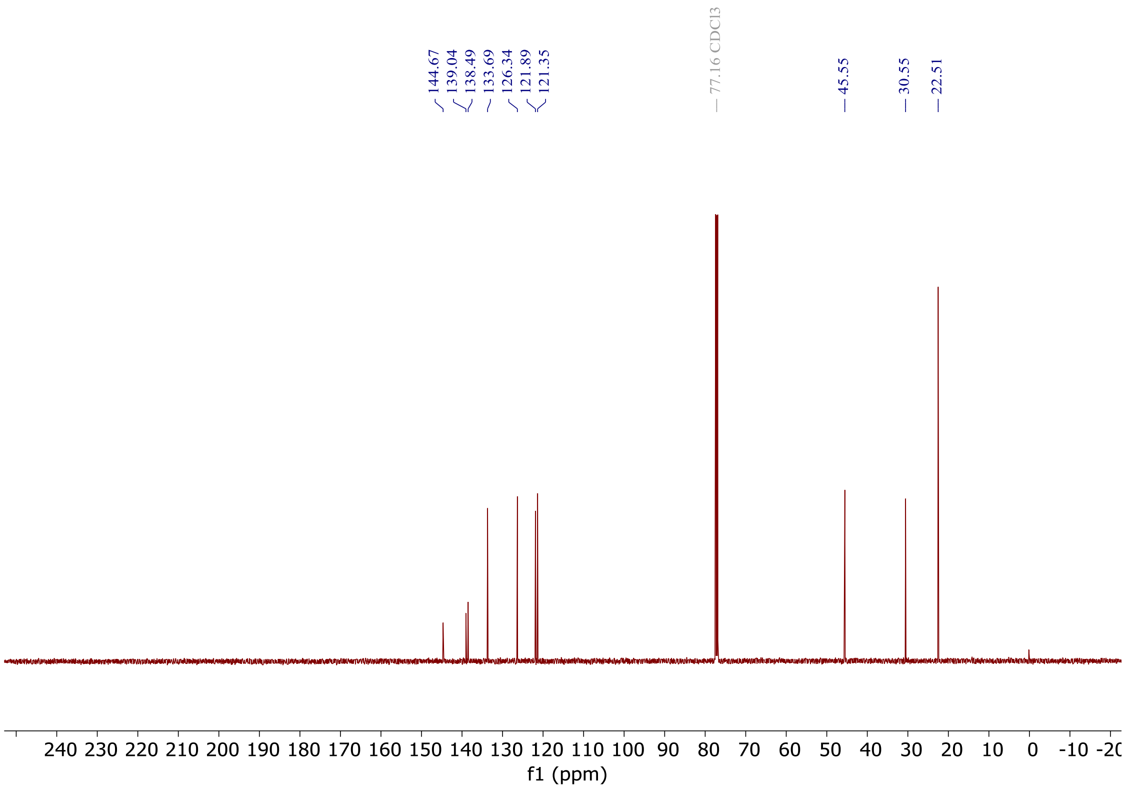

^{13}**C-NMR** spectrum of compound **S6**.

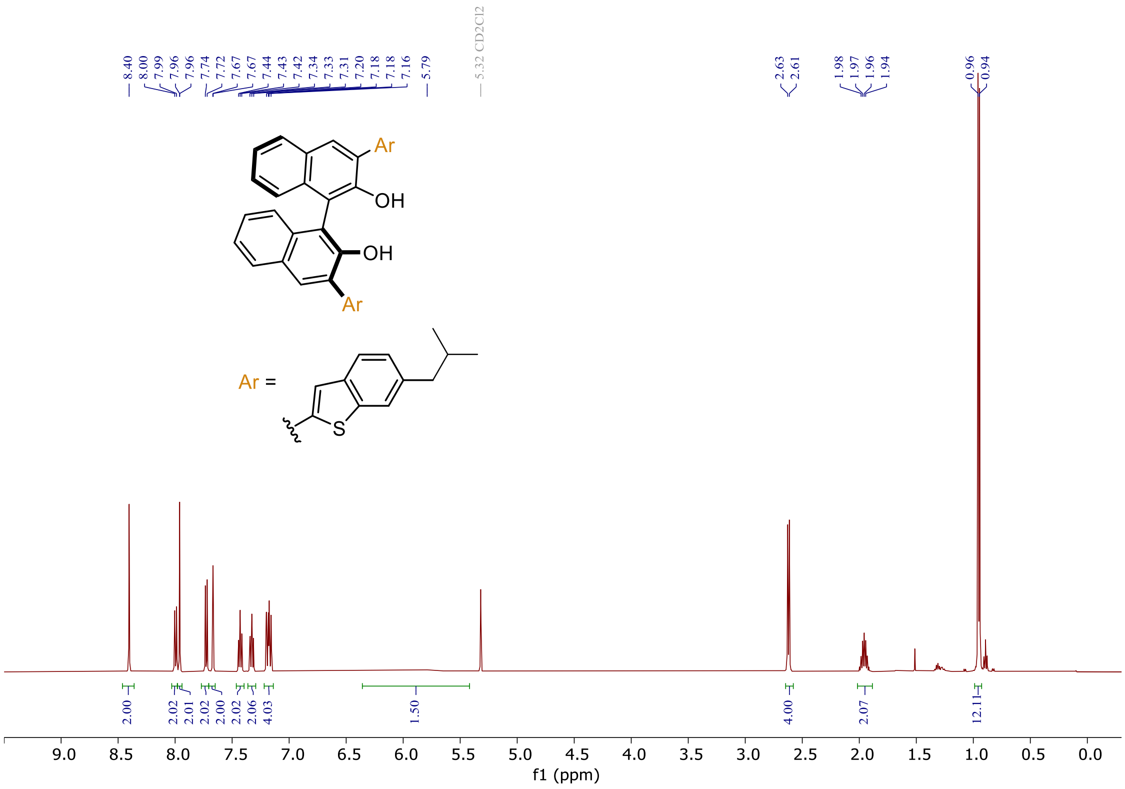

^{1}**H-NMR** spectrum of compound **S7.**

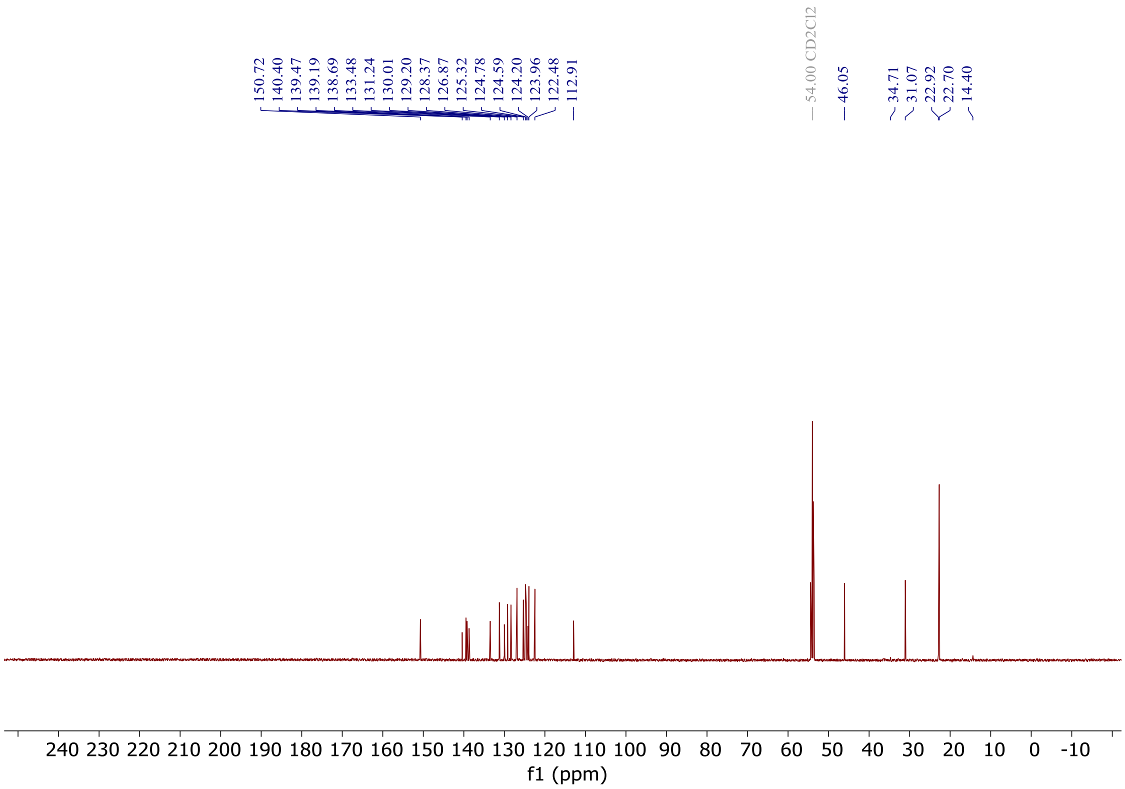

^{13}C-NMR spectrum of compound **S7**.

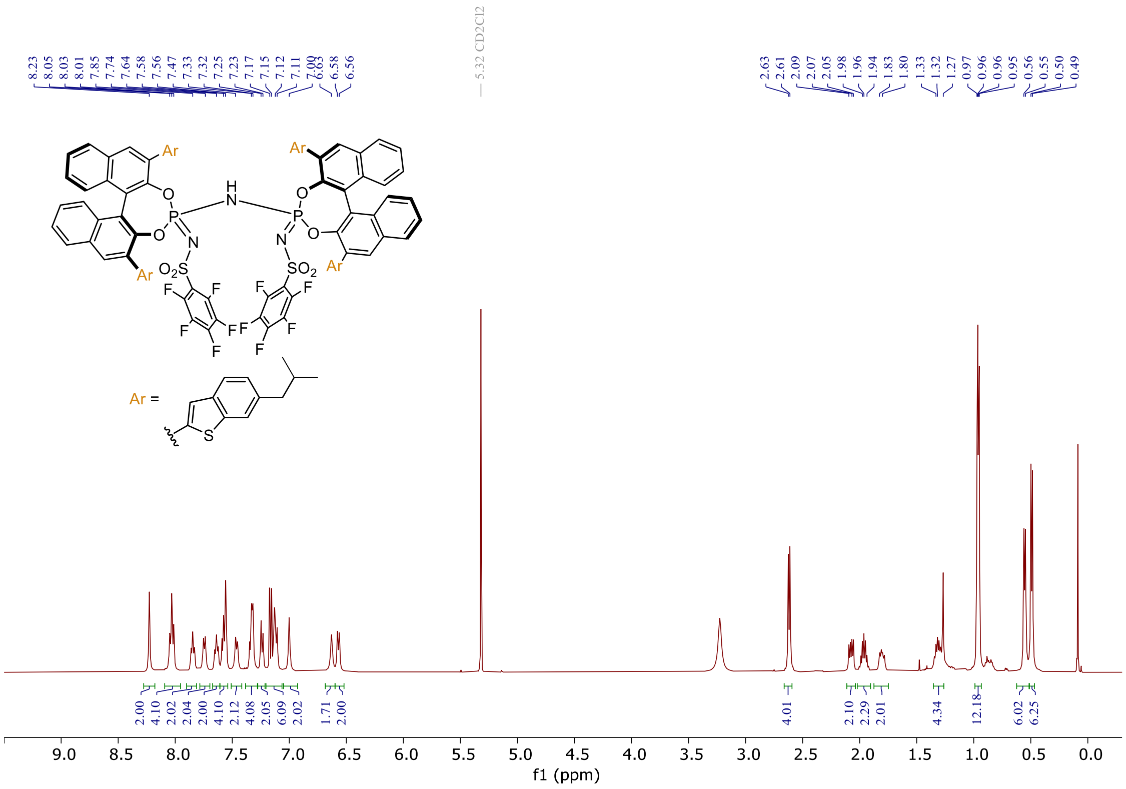

^{1}H-NMR spectrum of compound **6b.**

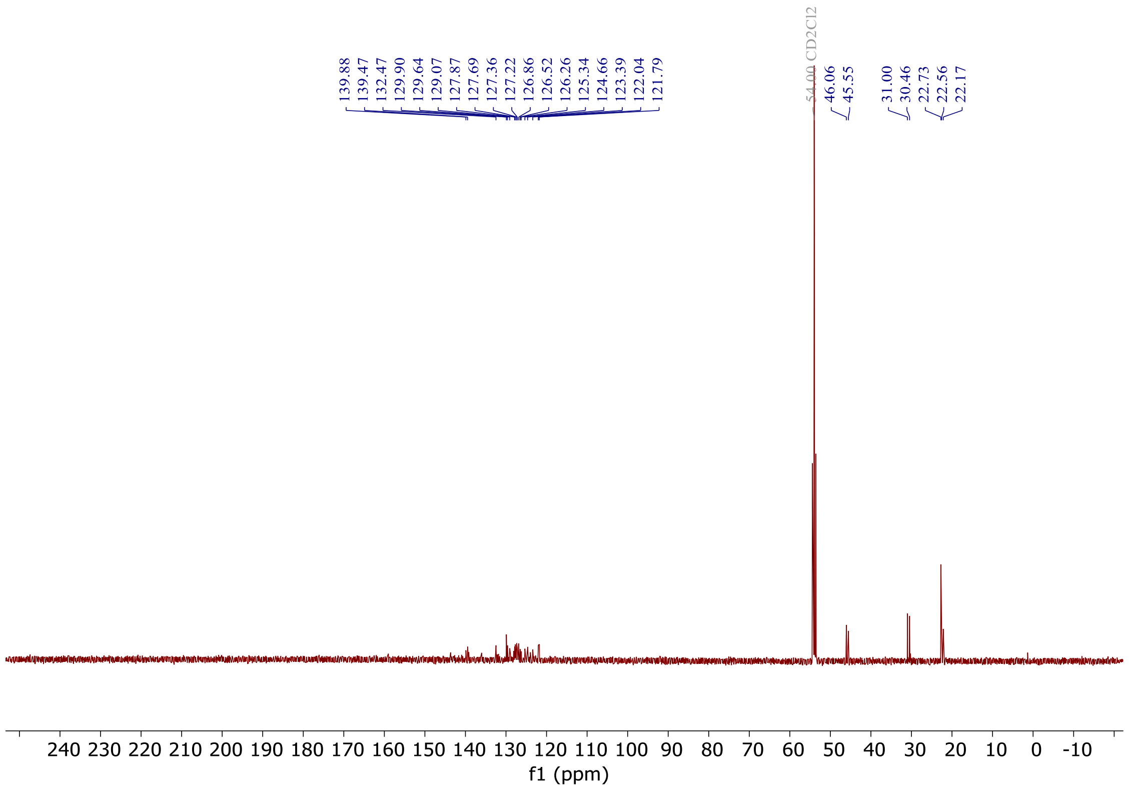

^{13}**C-NMR** spectrum of compound **6b**.

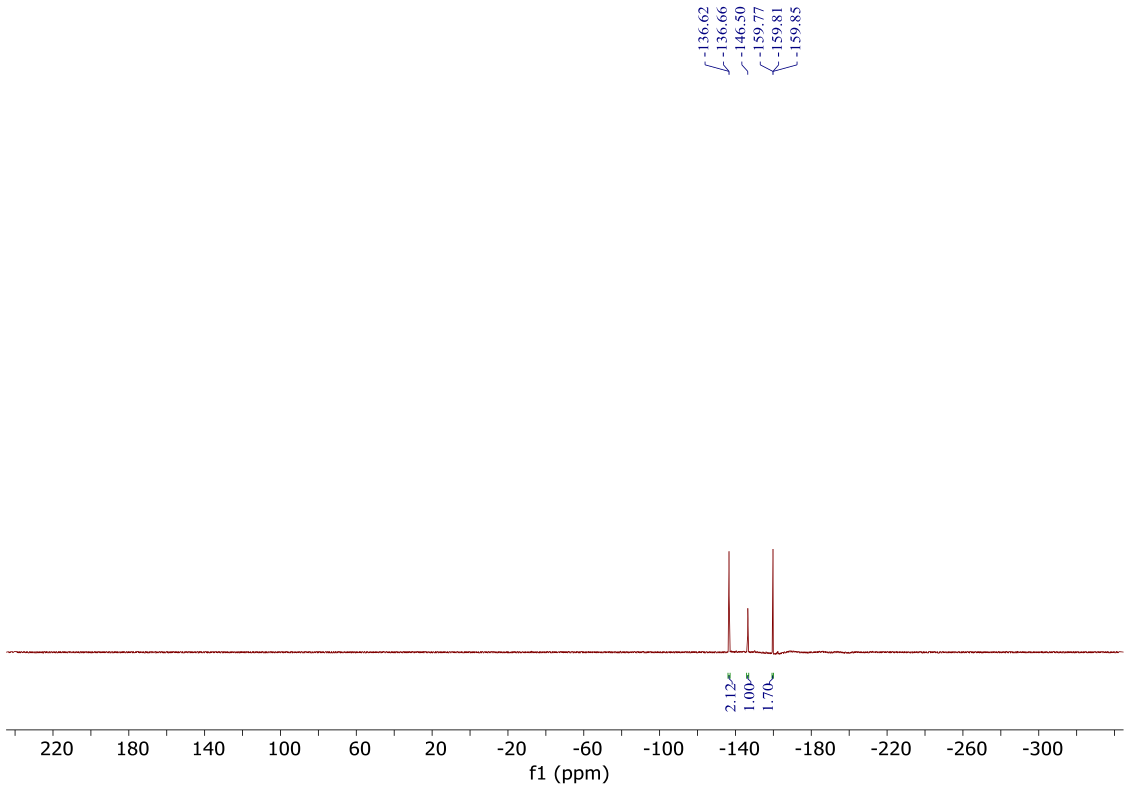

^{19}**F-NMR** spectrum of compound **6b**.

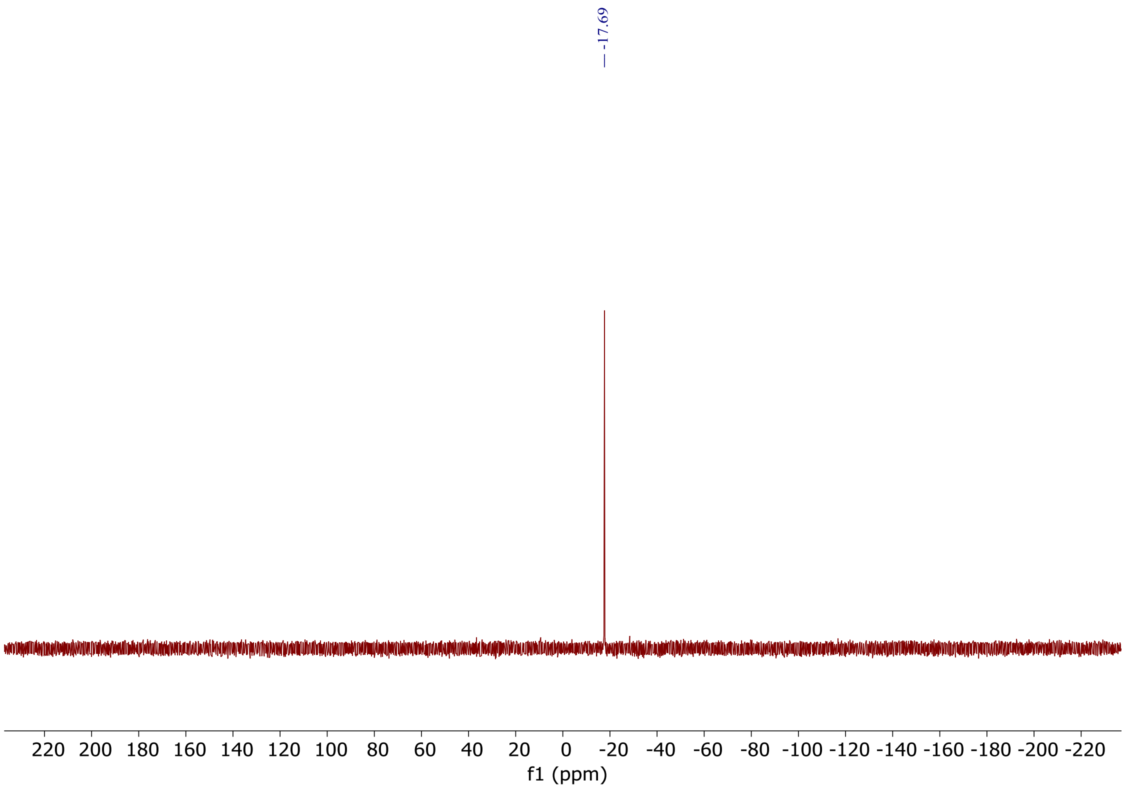

^{31}P-NMR spectrum of compound **6b**.

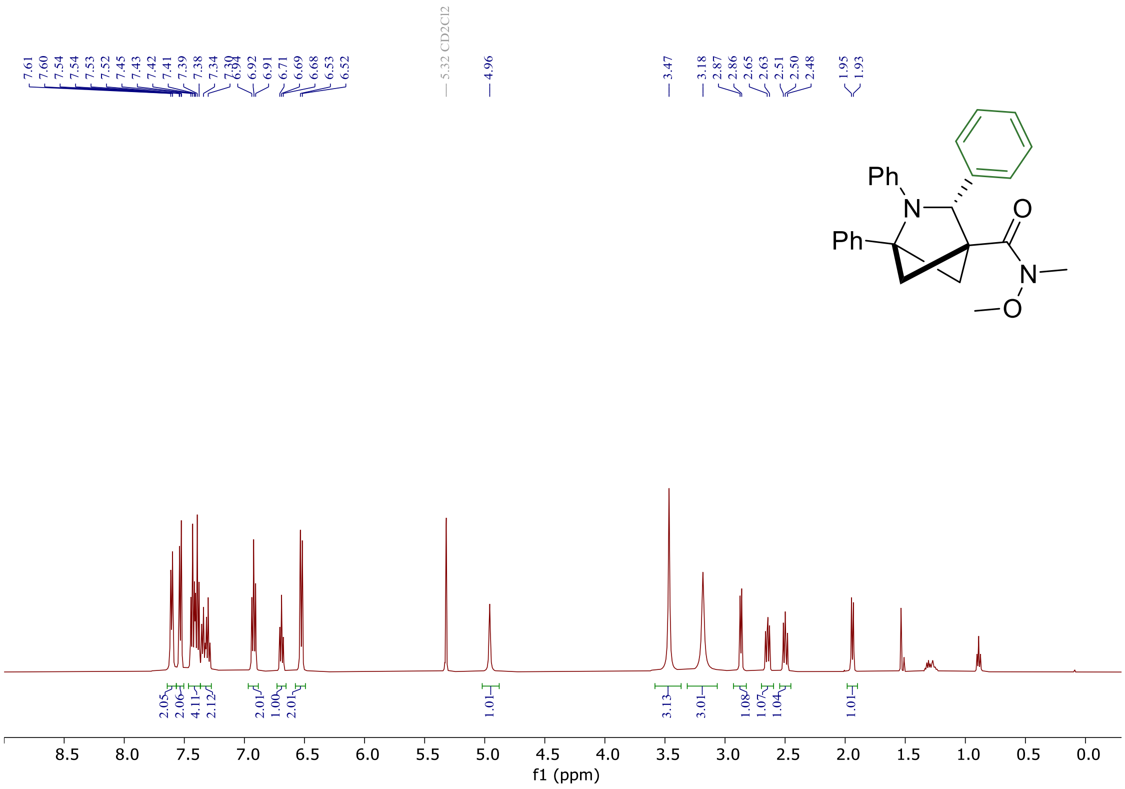

^{1}H-NMR spectrum of compound **3a.**

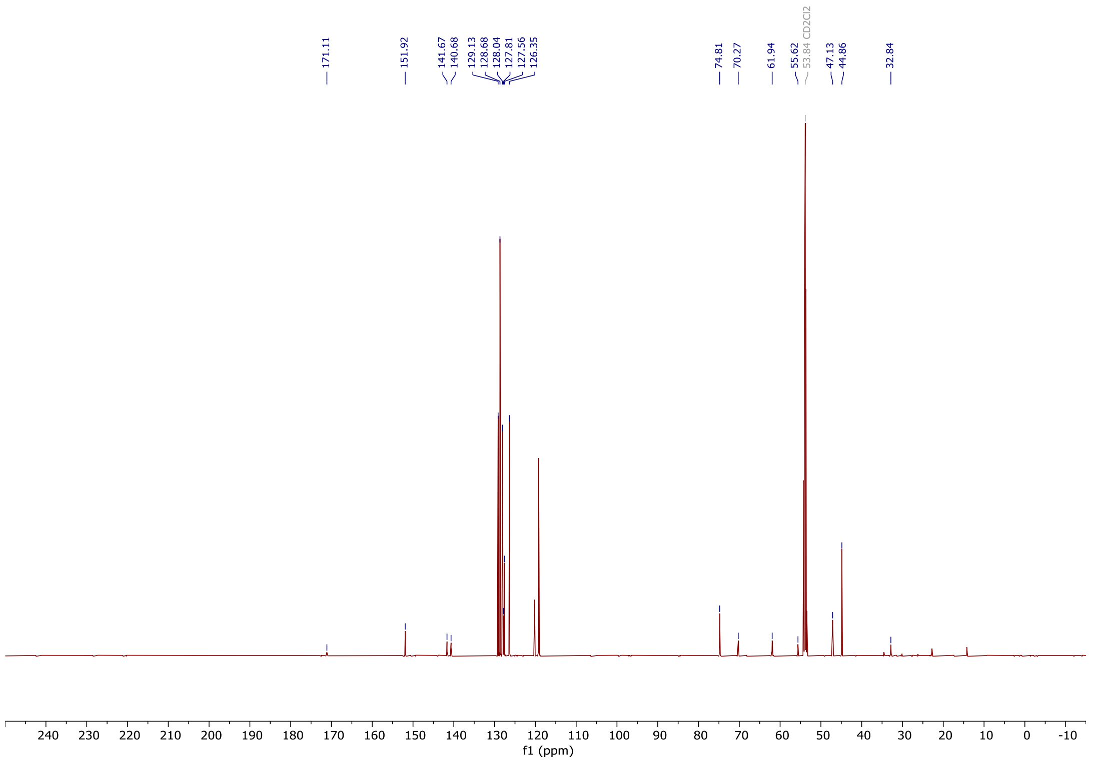

^{13}C-NMR spectrum of compound **3a**.

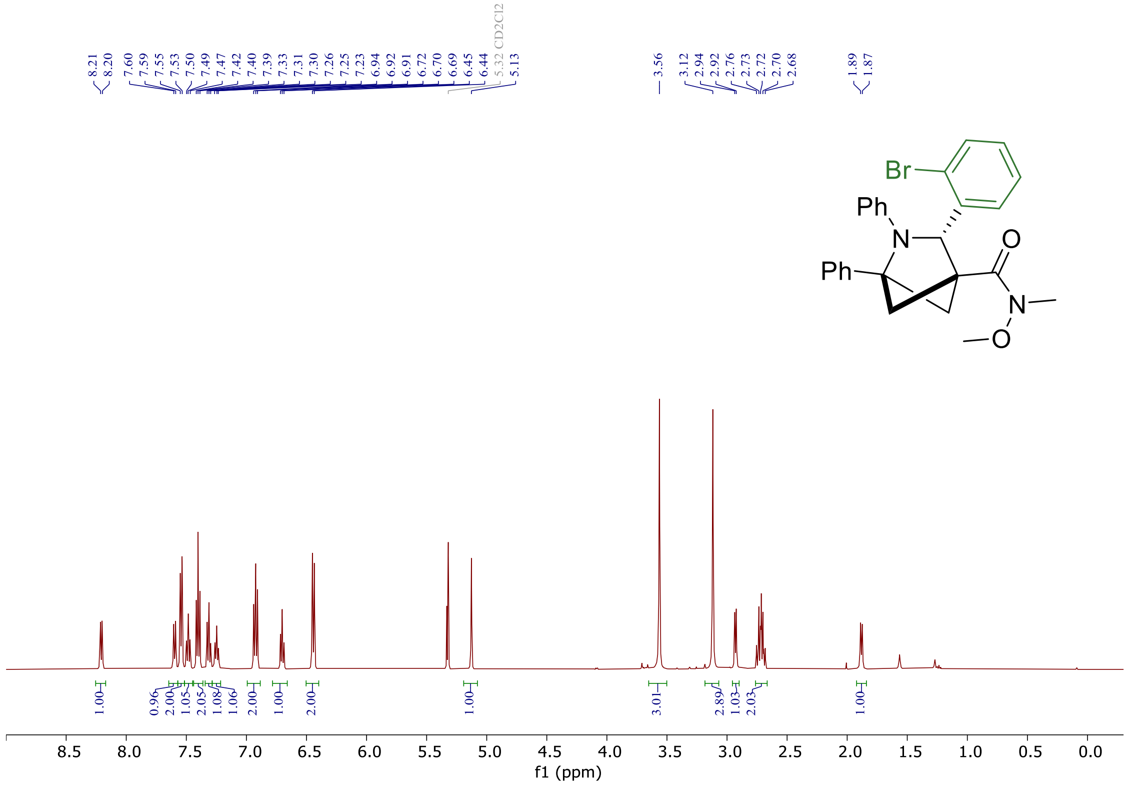

^{1}H-NMR spectrum of compound **3b.**

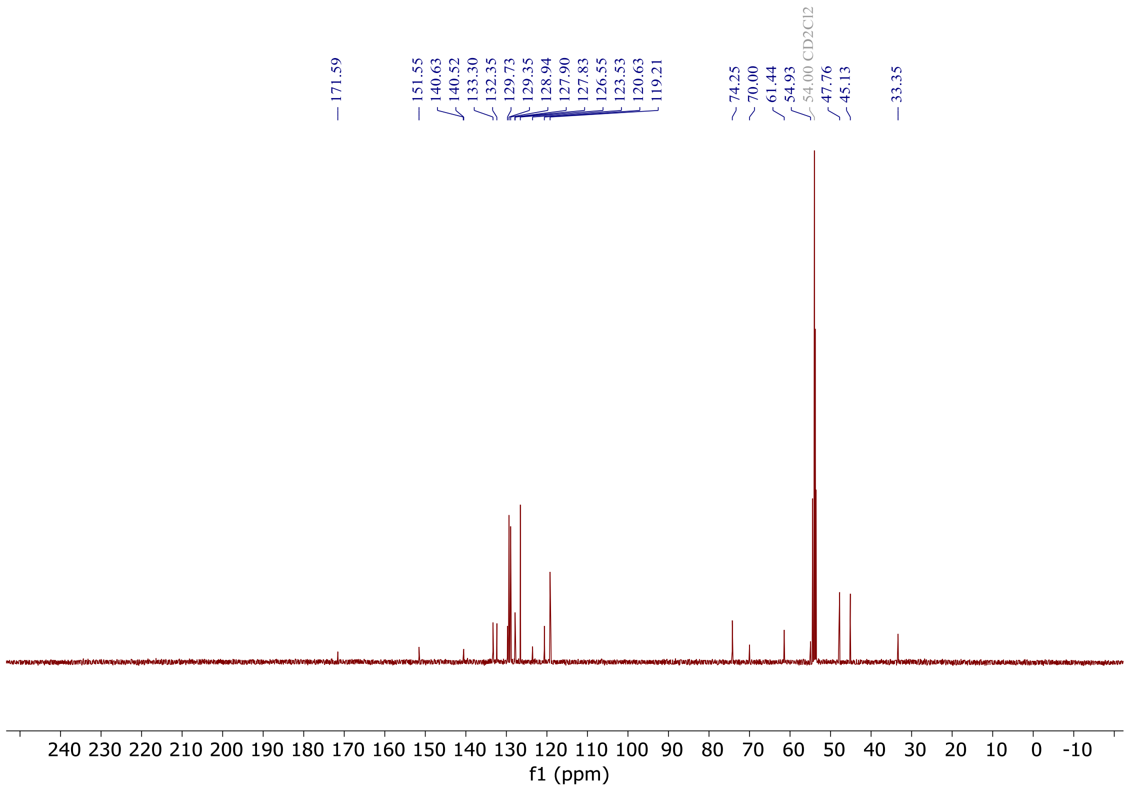

^{13}C-NMR spectrum of compound **3b.**

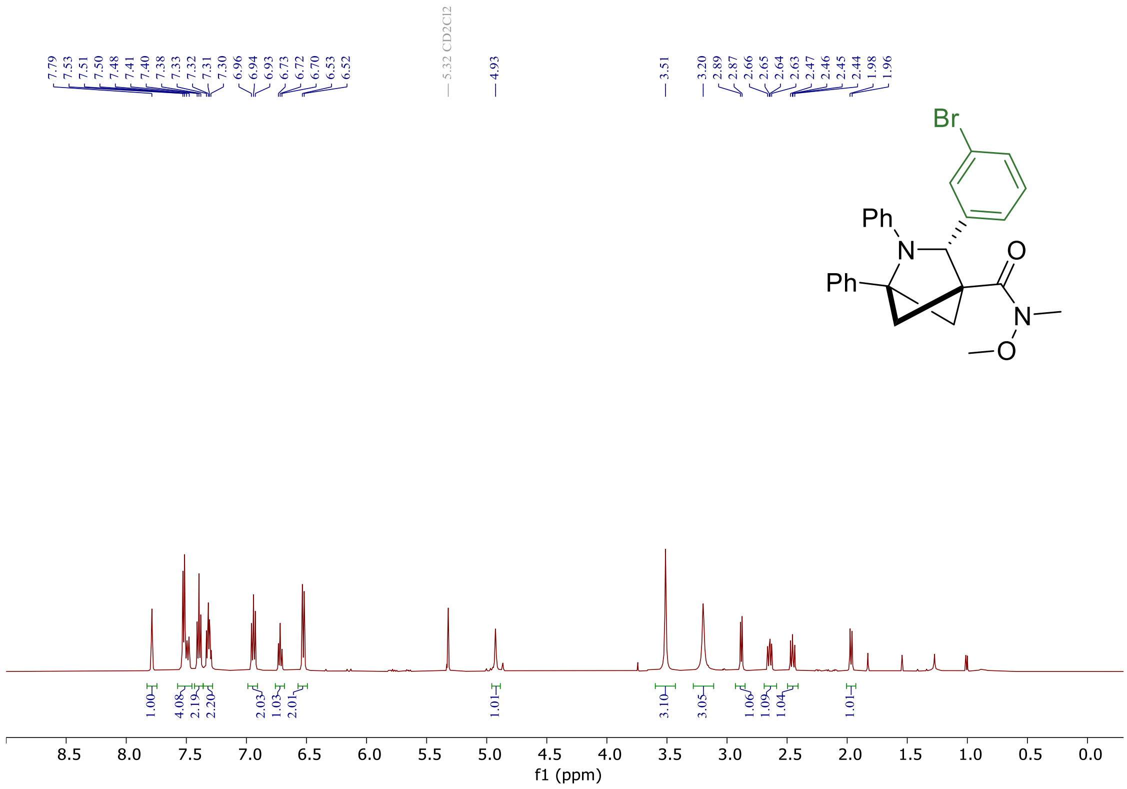

^{1}H-NMR spectrum of compound **3c.**

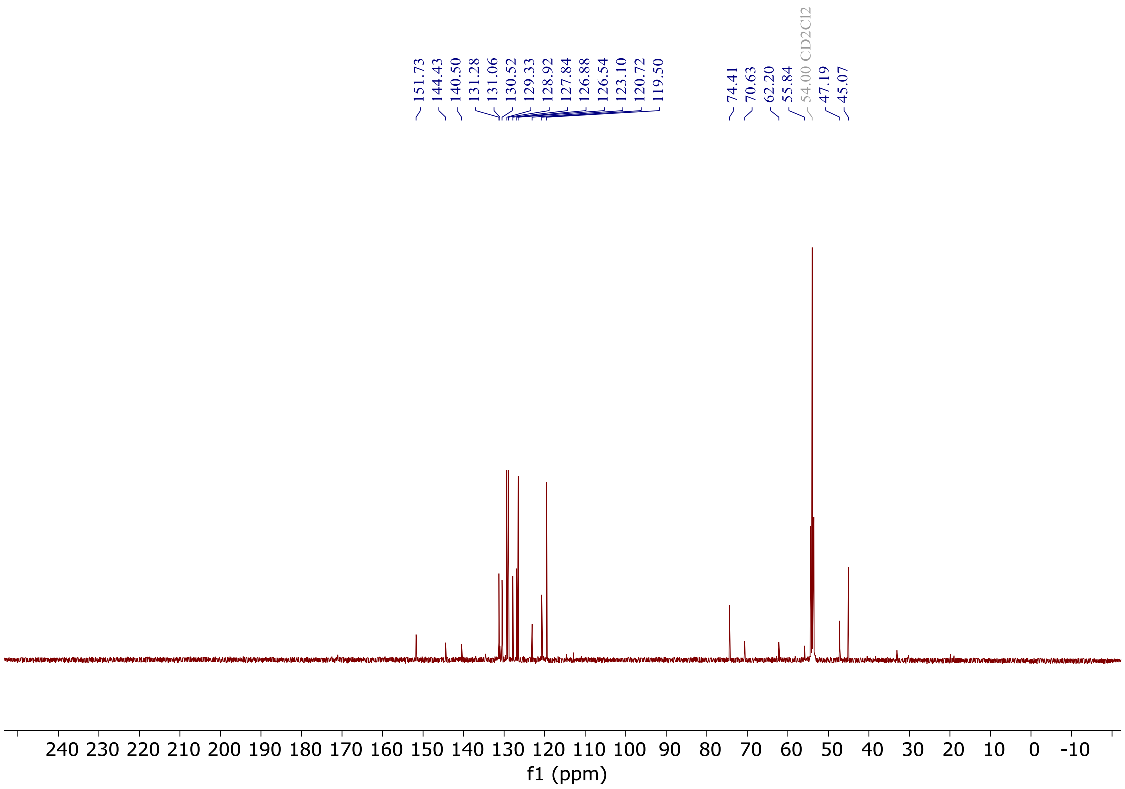

^{13}C-NMR spectrum of compound **3c.**

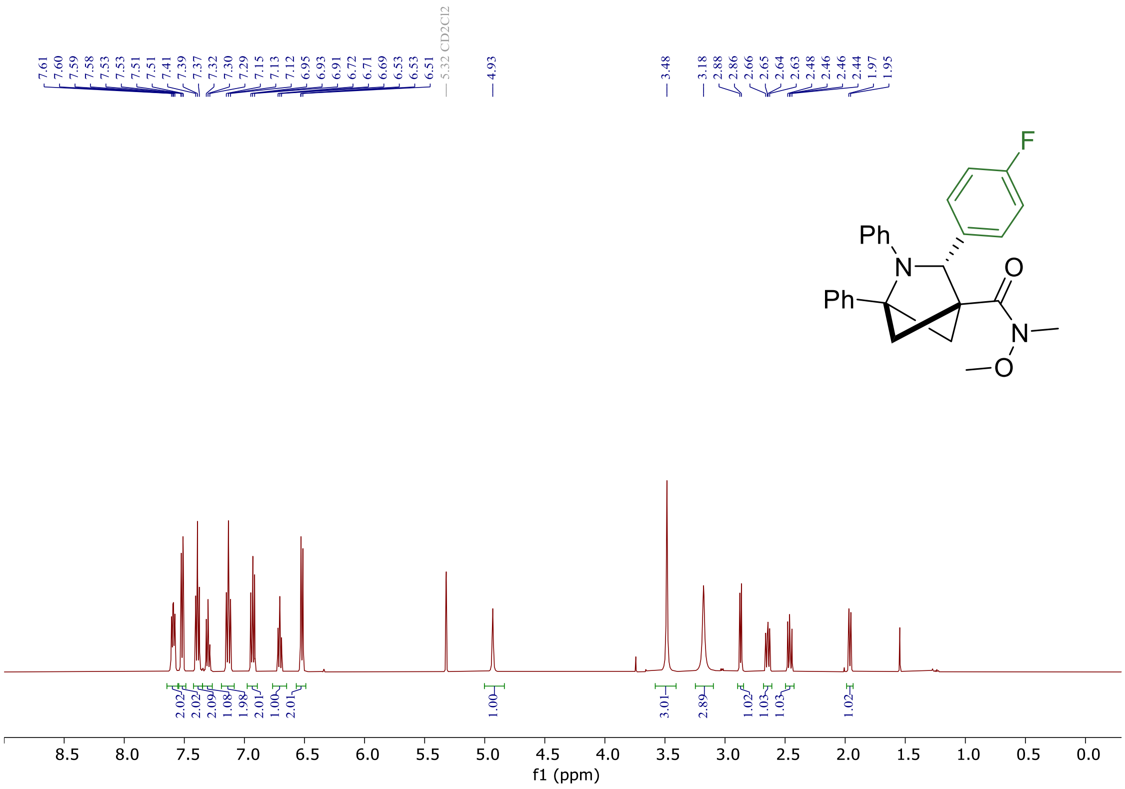

^{1}H-NMR spectrum of compound **3d.**

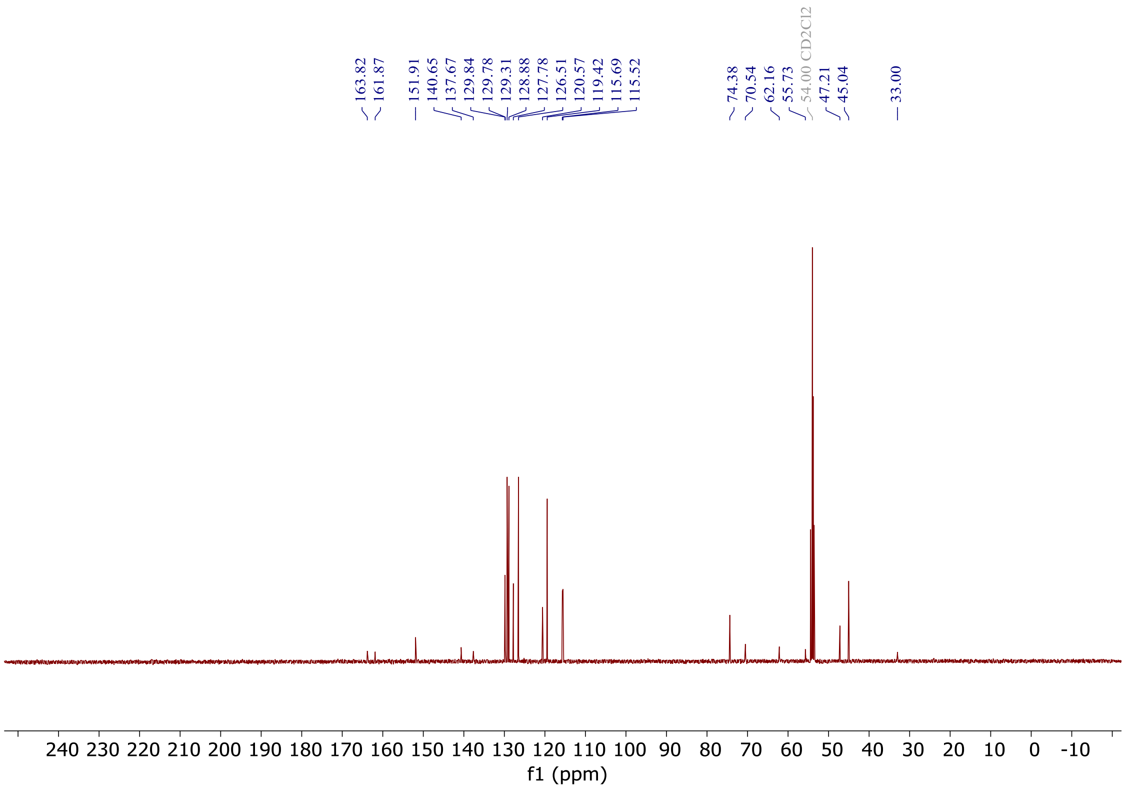

^{13}C-NMR spectrum of compound **3d.**

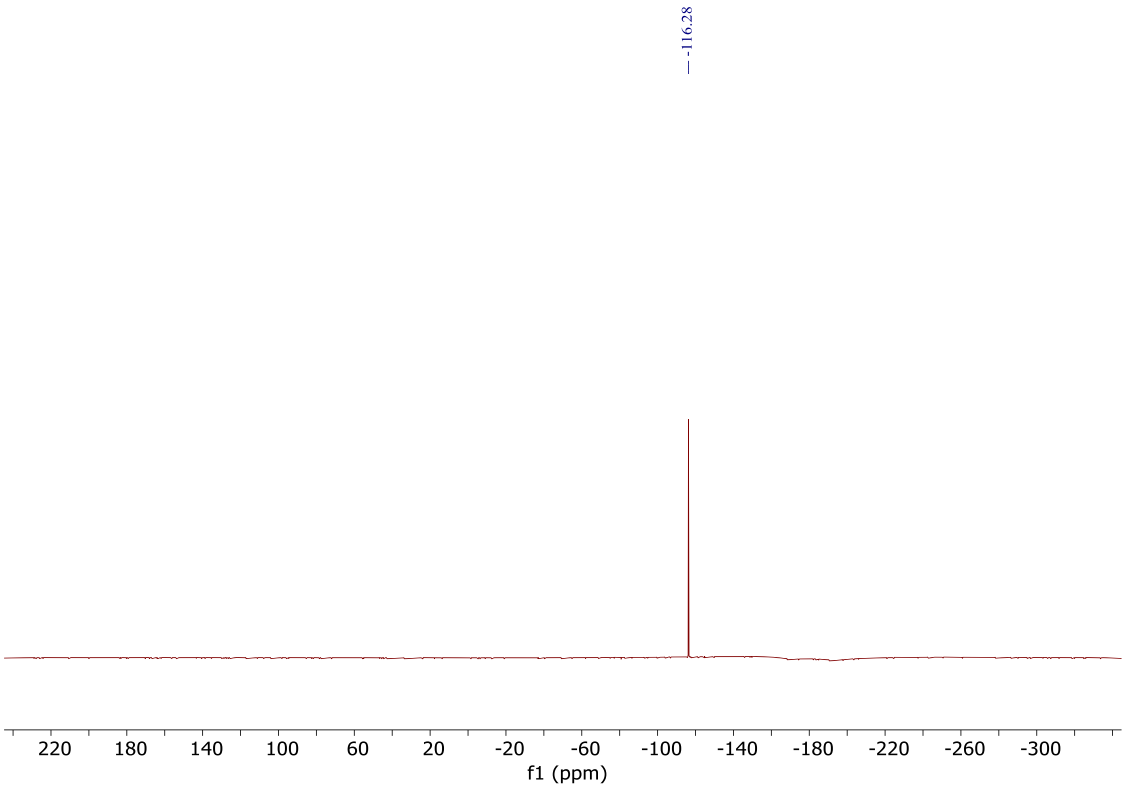

^{19}F-NMR spectrum of compound **3d.**

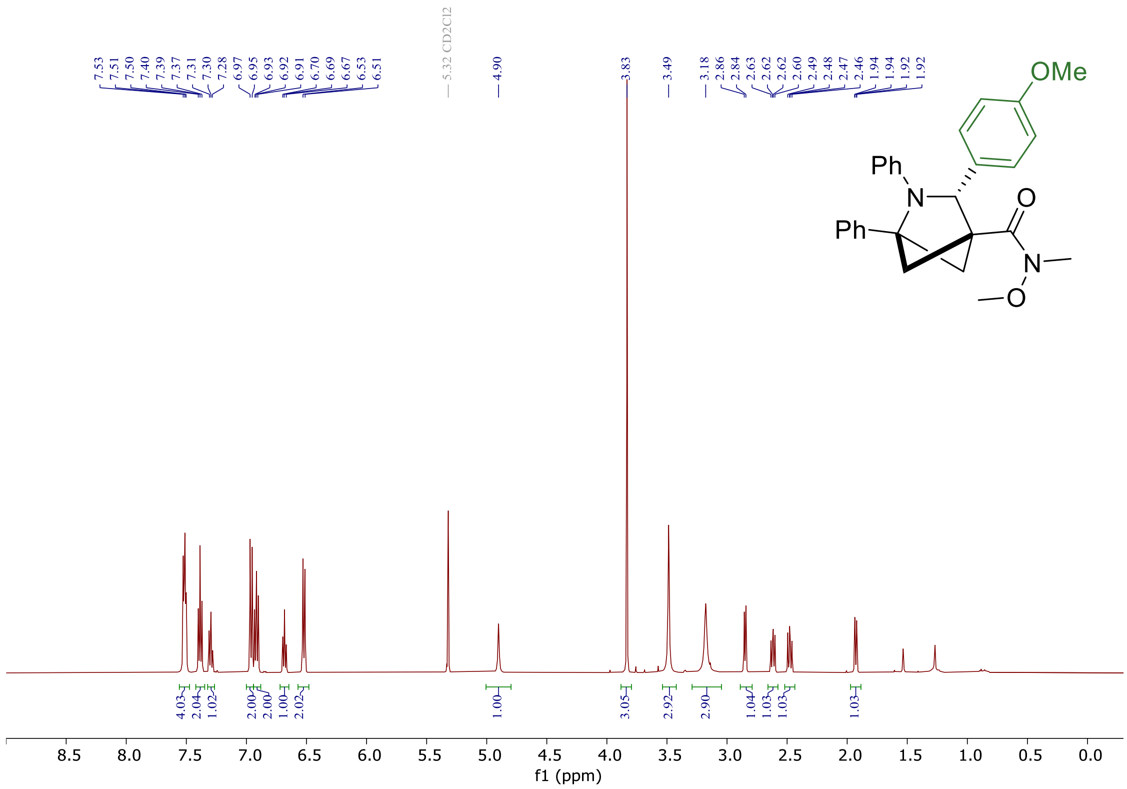

^{1}H-NMR spectrum of compound **3e.**

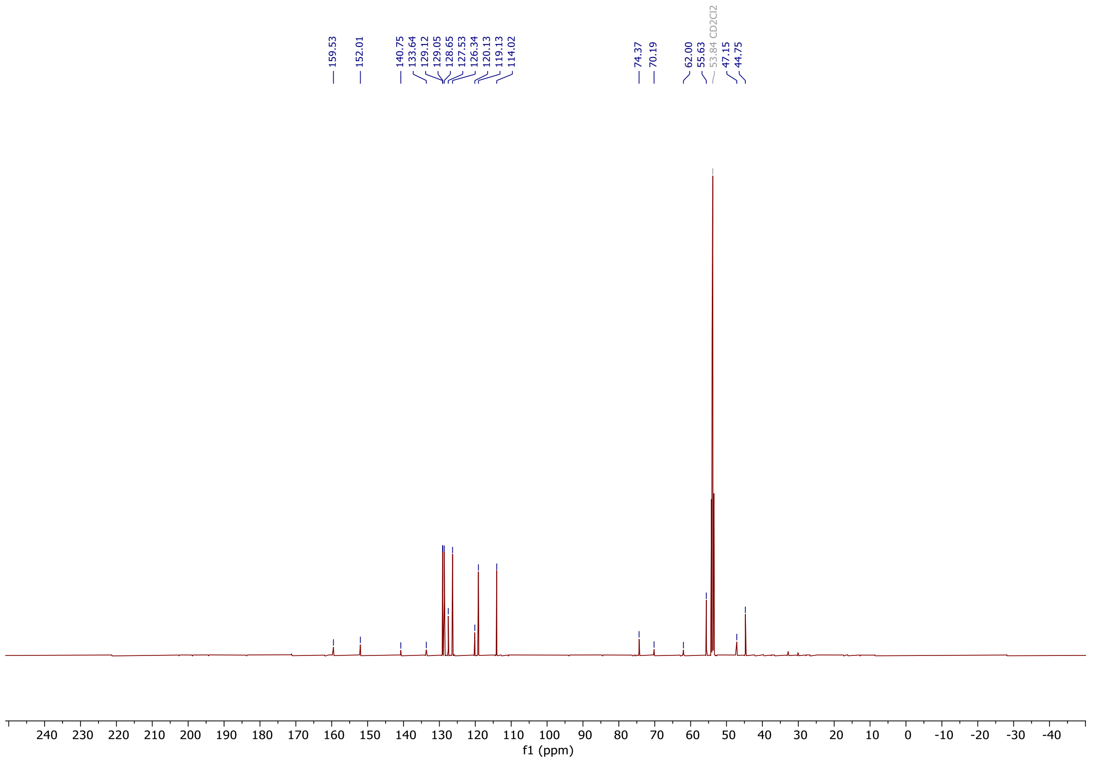

^{13}C-NMR spectrum of compound **3e.**

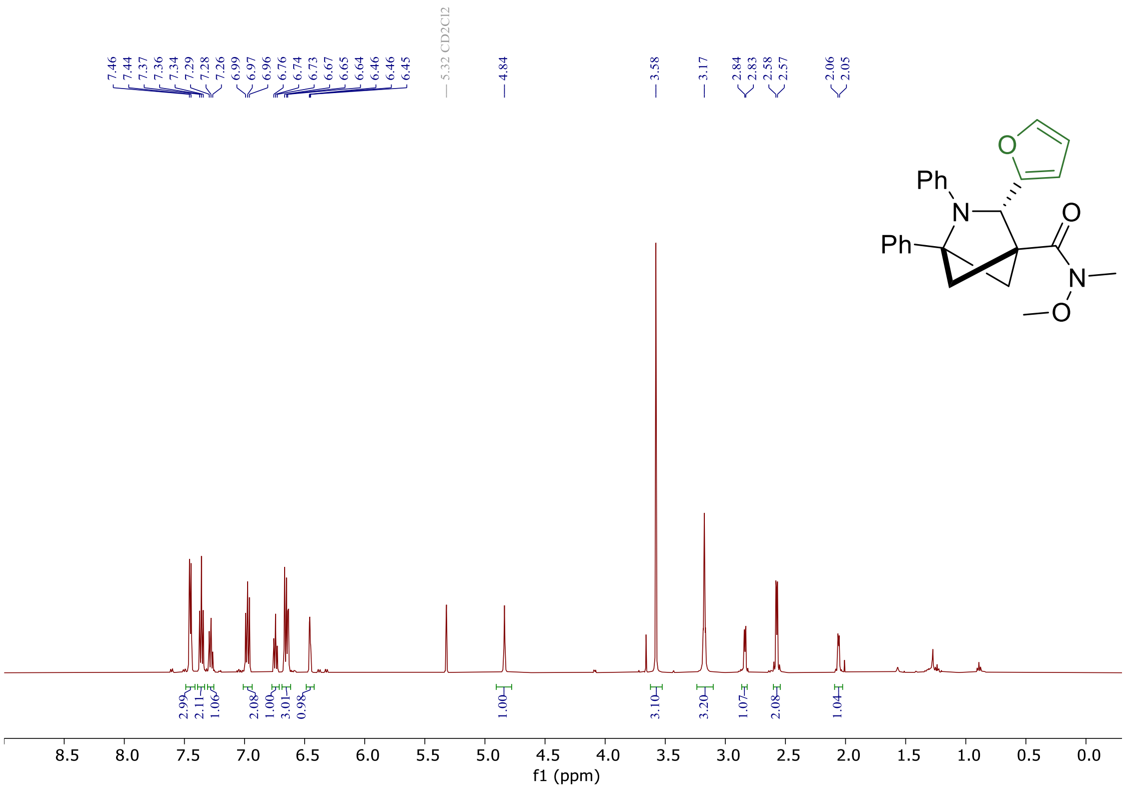

^{1}H-NMR spectrum of compound **3f.**

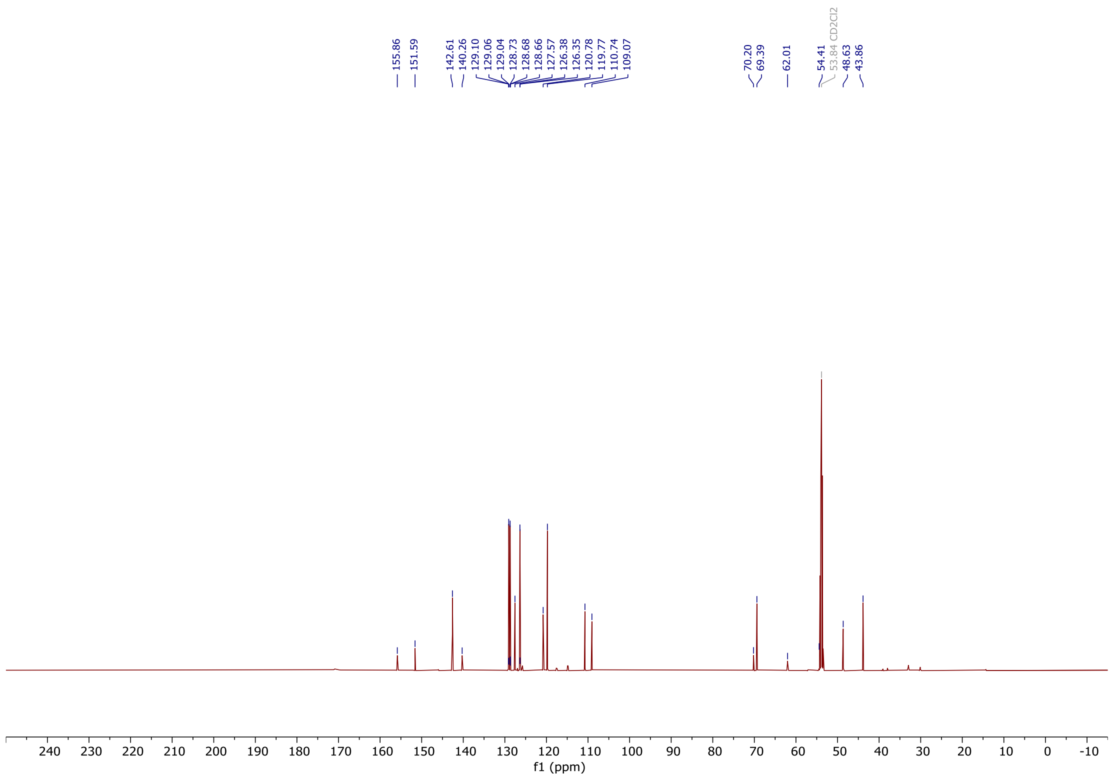

^{13}C-NMR spectrum of compound **3f.**

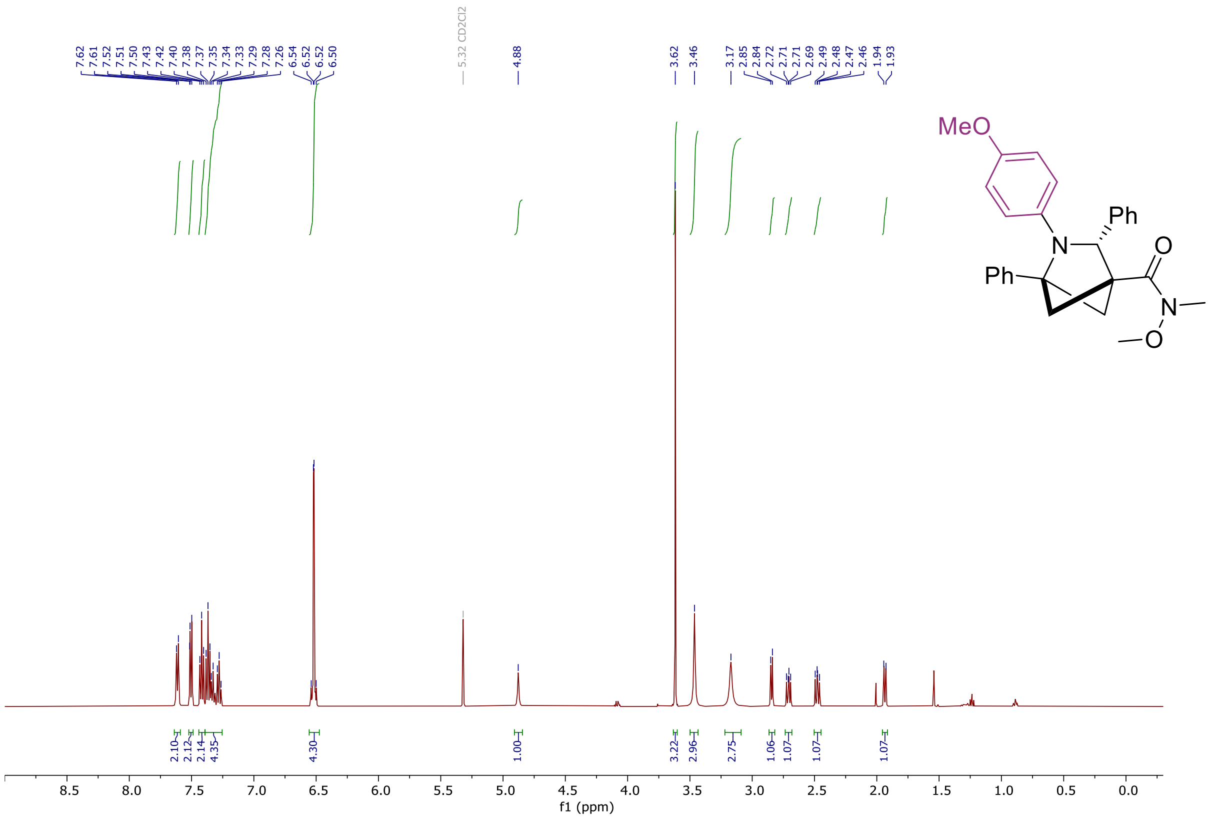

^{1}H-NMR spectrum of compound **3g**.

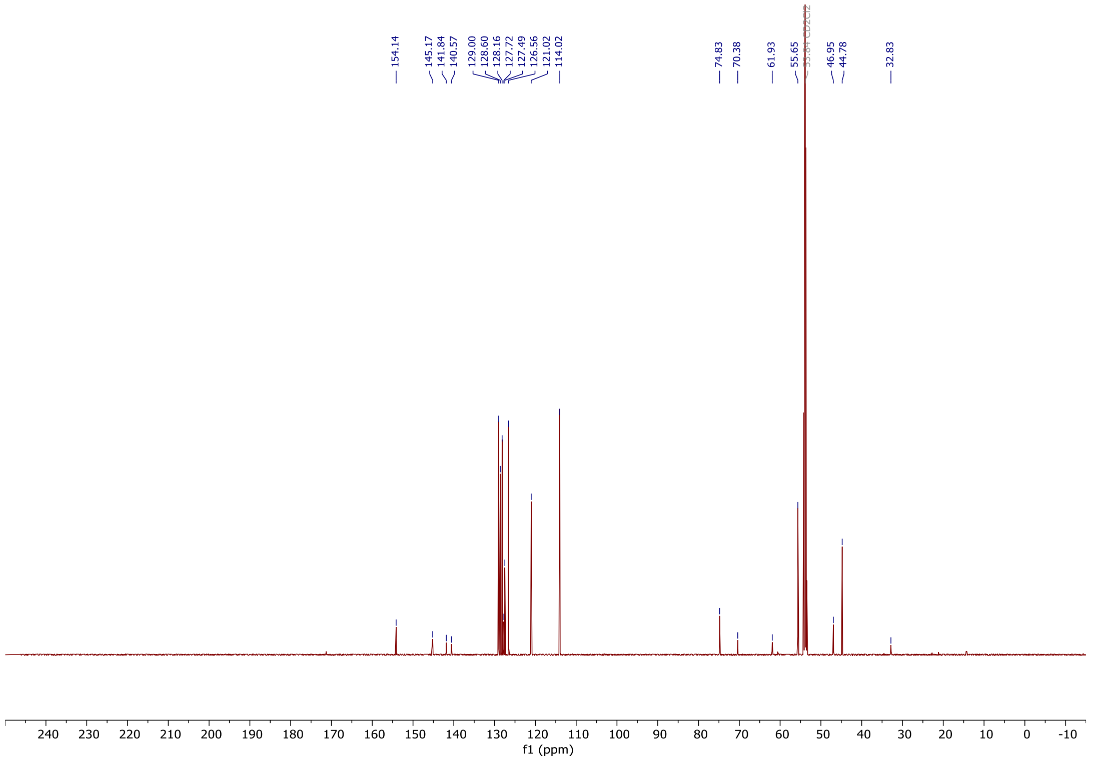

^{13}C-NMR spectrum of compound 3g.

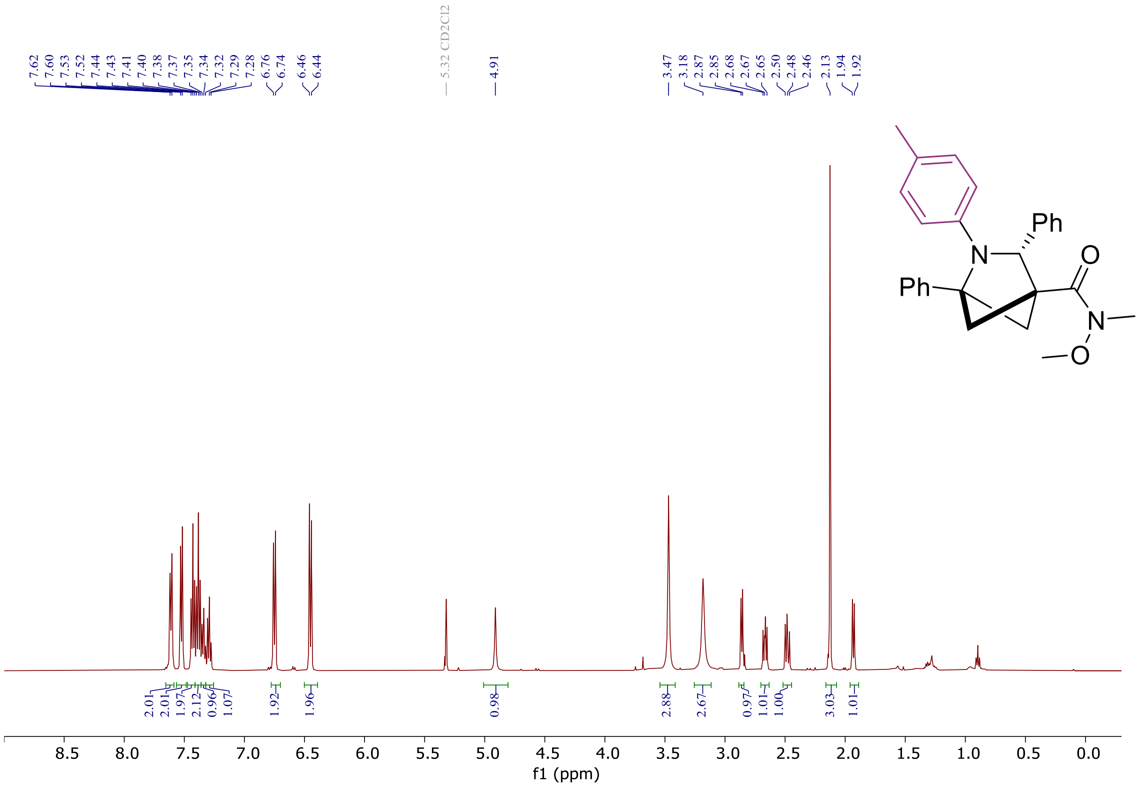

^{1}H-NMR spectrum of compound **3h.**

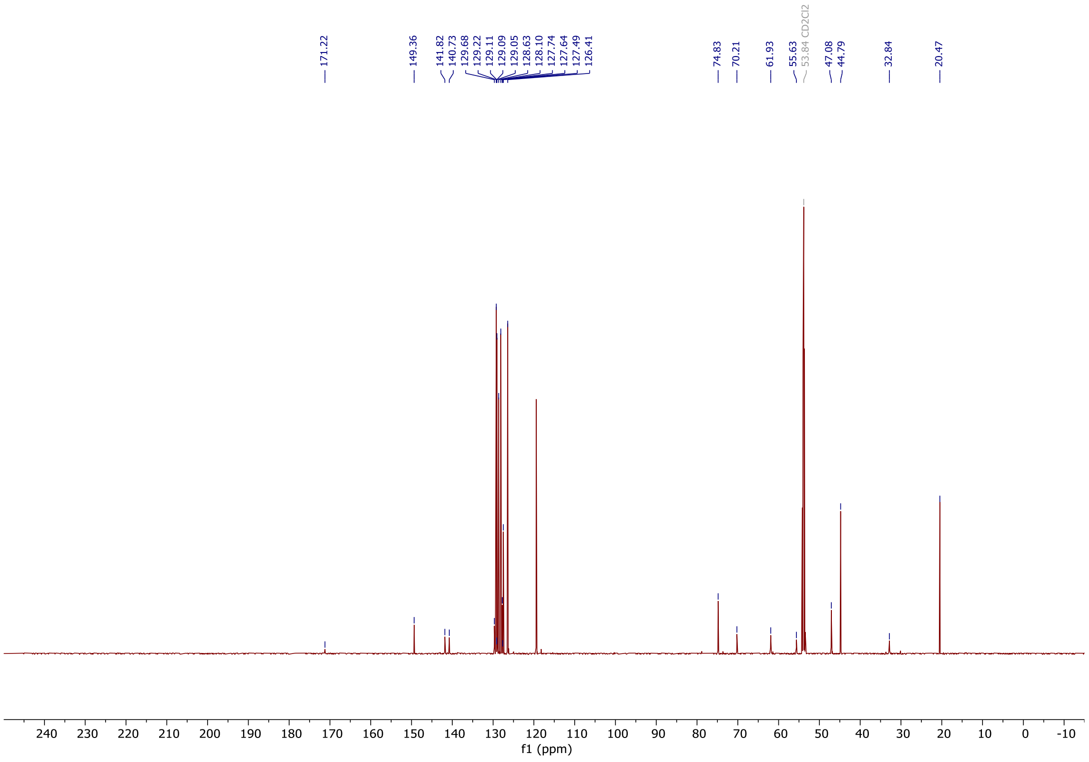

^{13}C-NMR spectrum of compound **3h.**

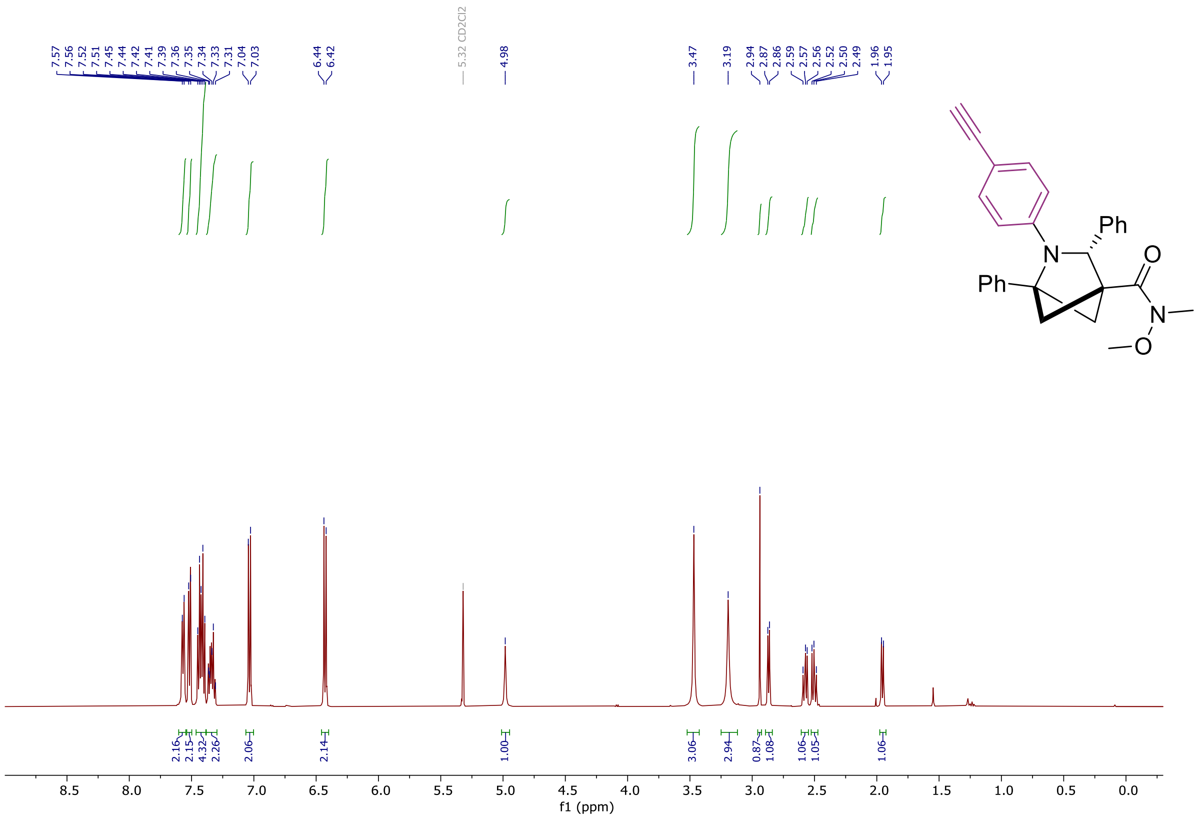

^{1}H-NMR spectrum of compound **3i.**

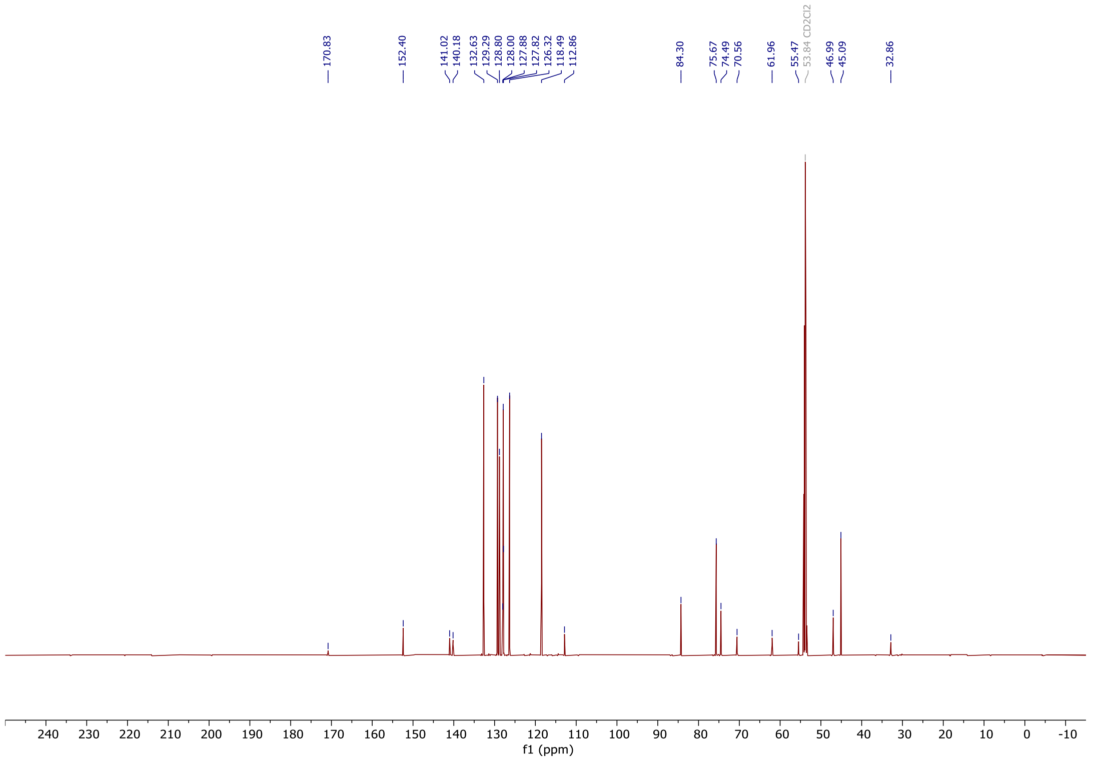

^{13}C-NMR spectrum of compound **3i.**

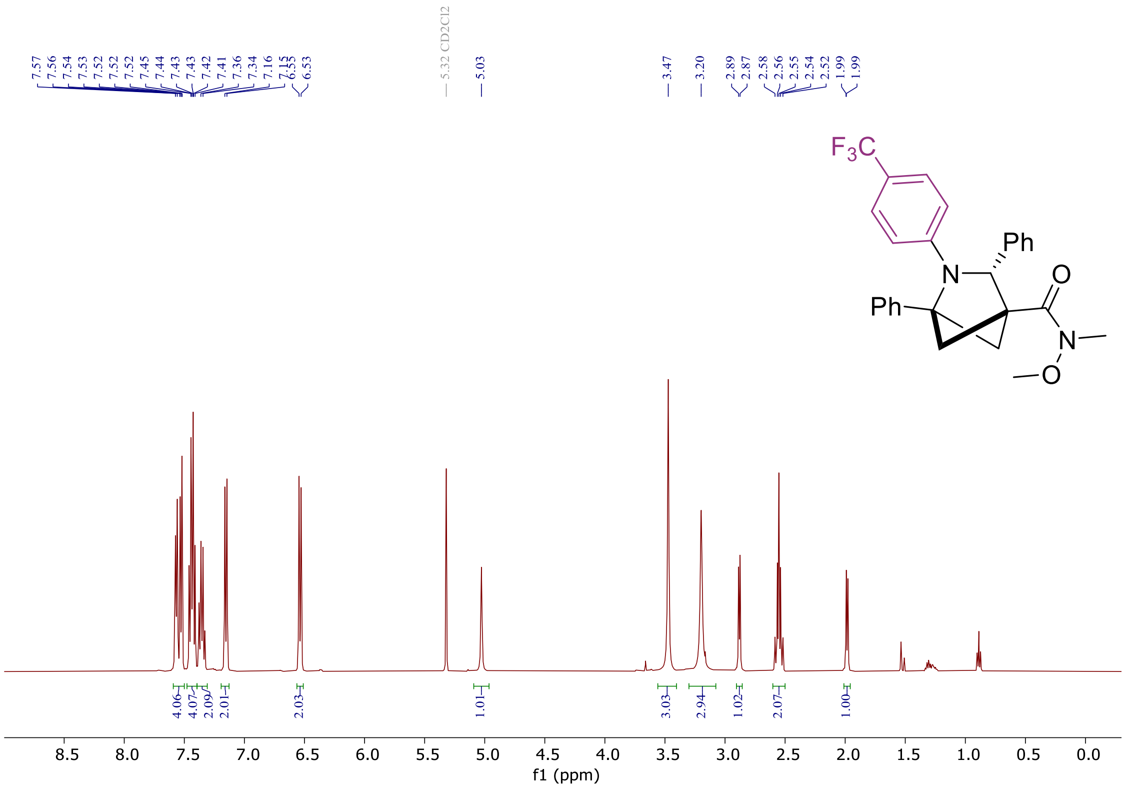

^{1}H-NMR spectrum of compound **3j.**

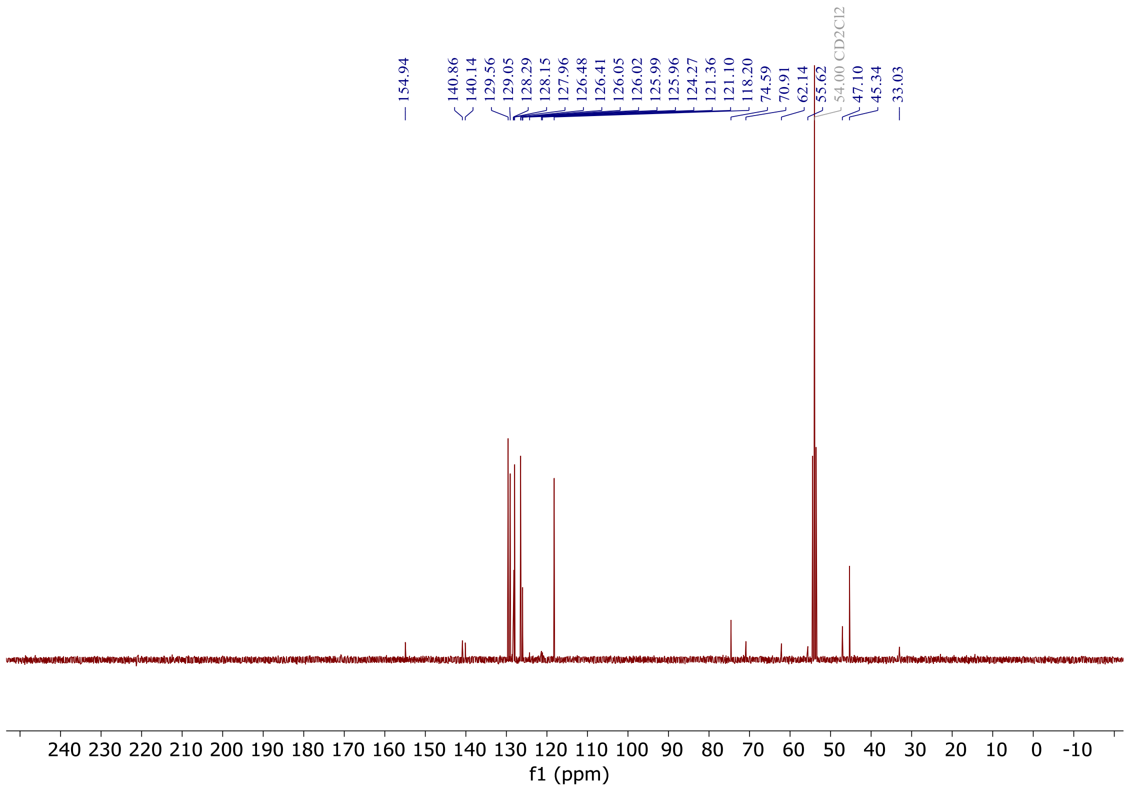

^{13}**C-NMR** spectrum of compound **3j.**

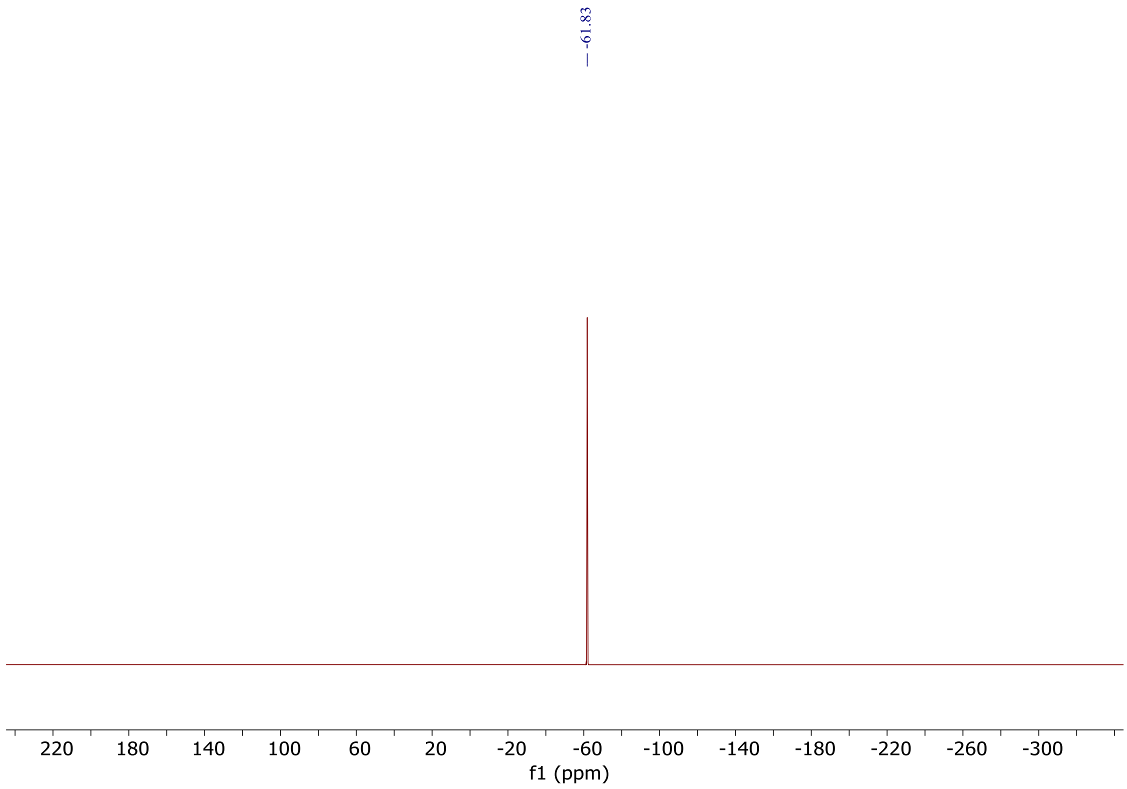

^{19}**F-NMR** spectrum of compound **3j.**

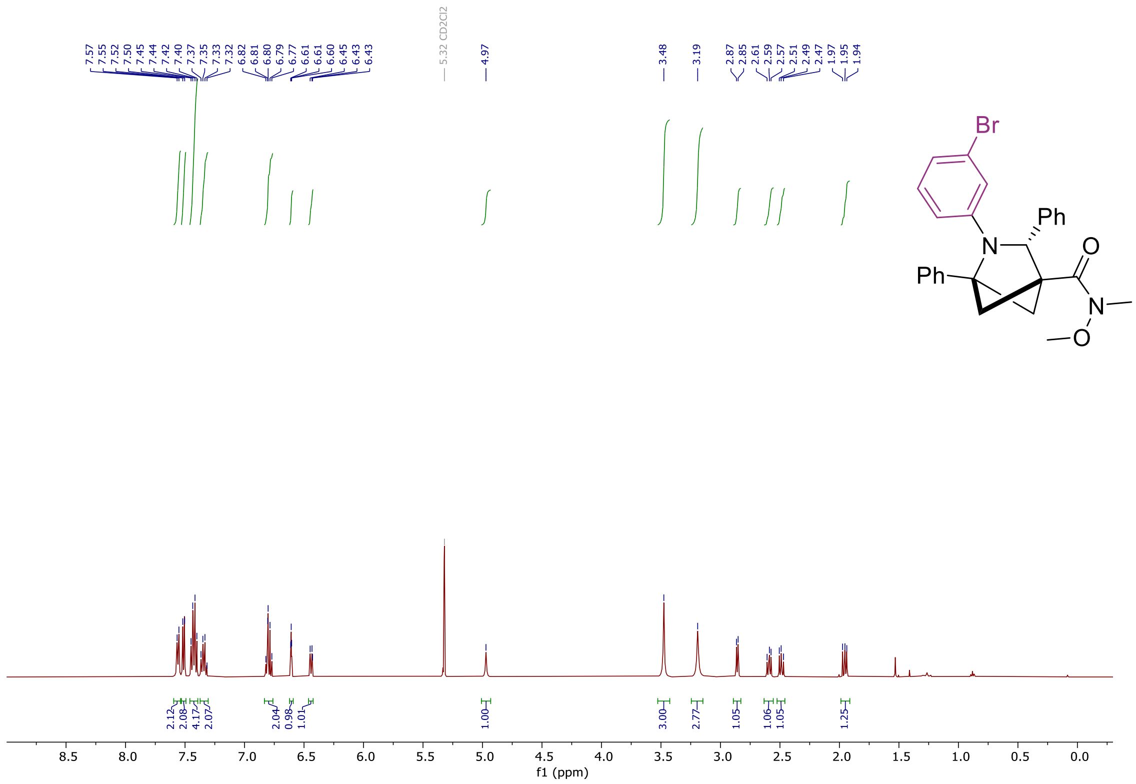

^{1}H-NMR spectrum of compound **3k.**

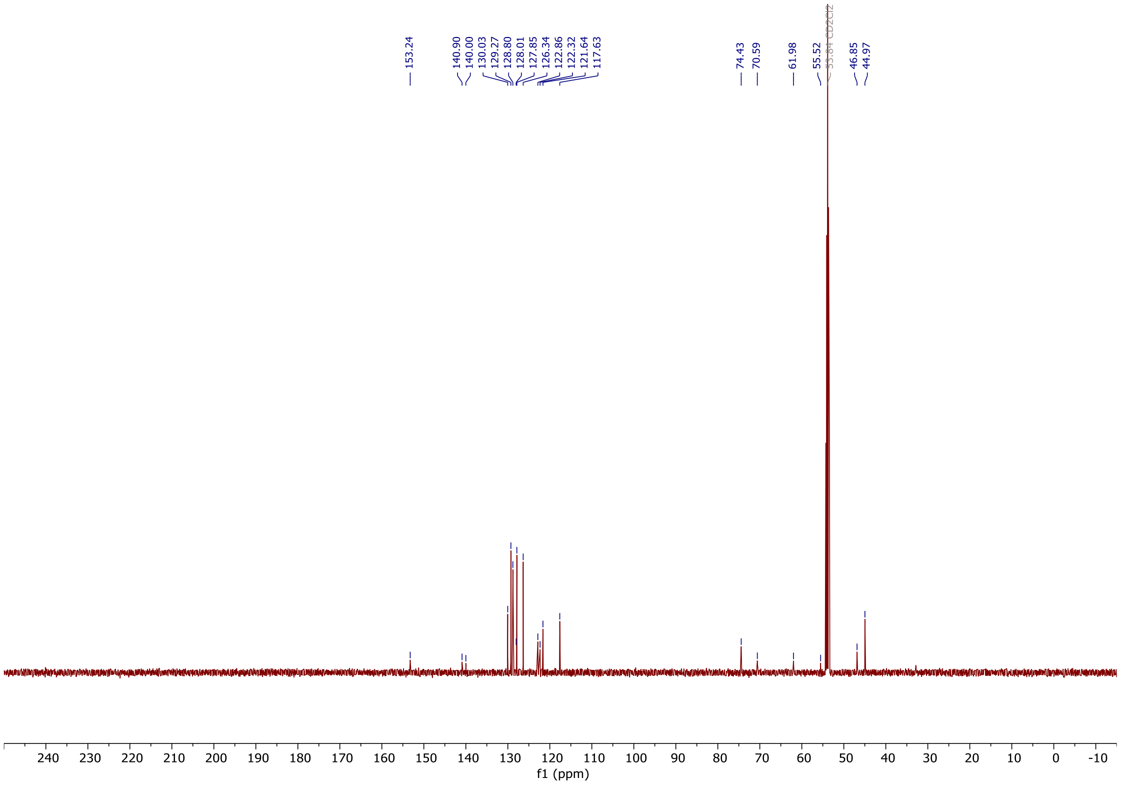

^{13}C-NMR spectrum of compound **3k.**

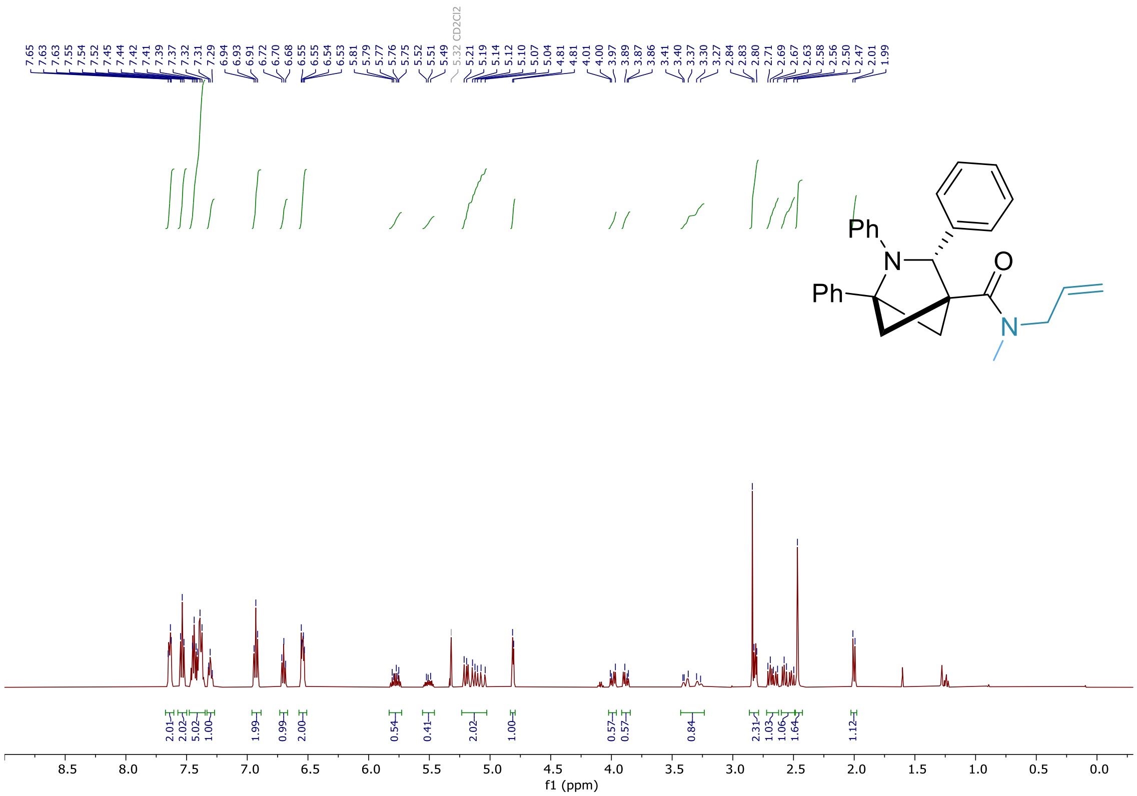

1H-NMR spectrum of compound **3l.**

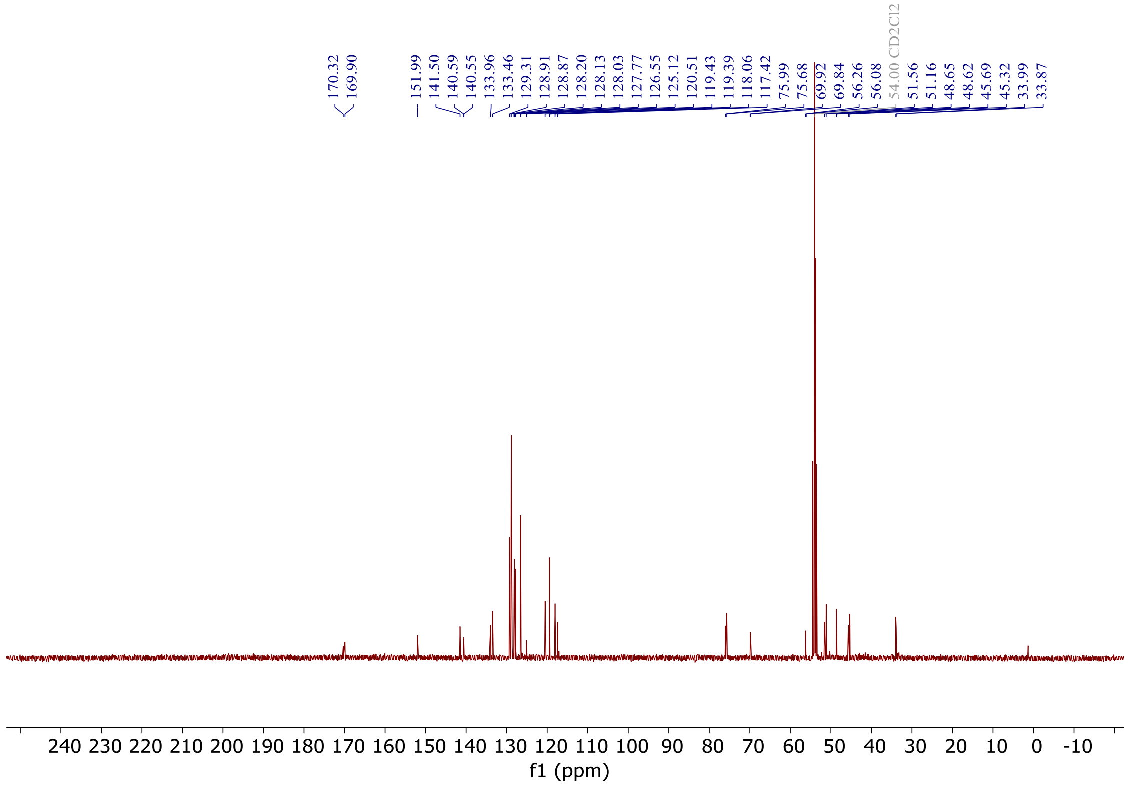

13C-NMR spectrum of compound **3l.**

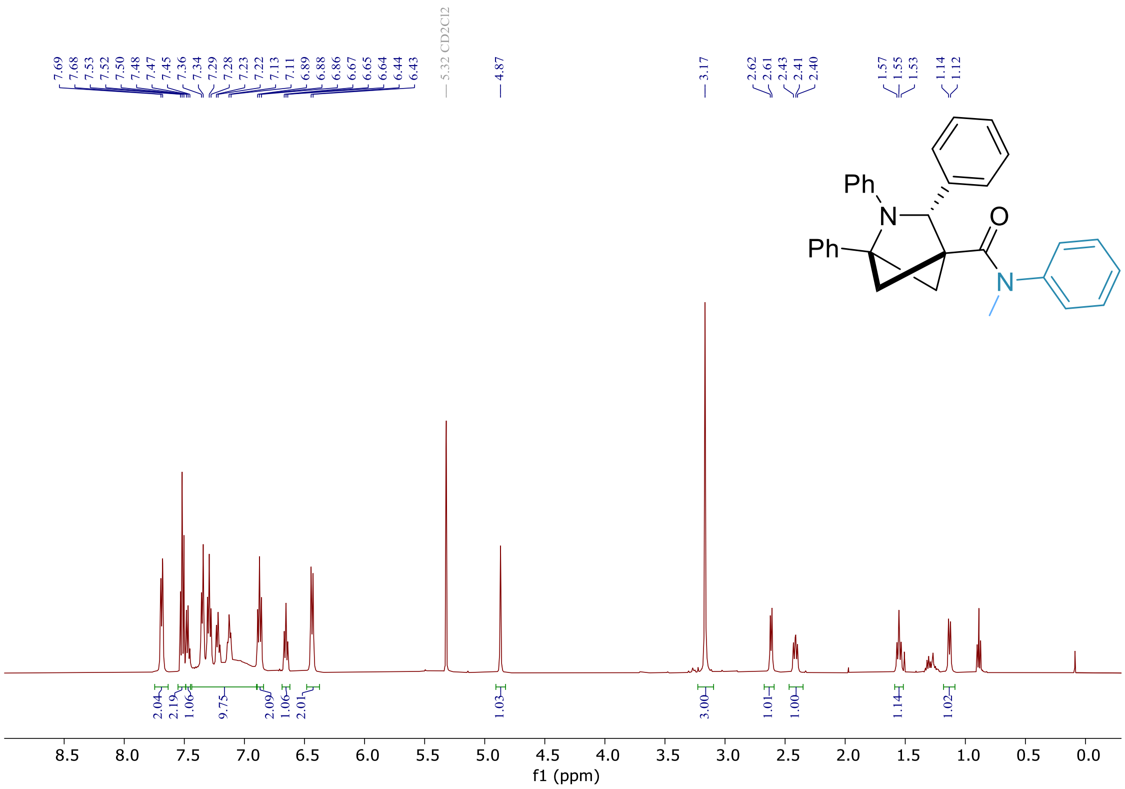

^{1}H-NMR spectrum of compound **3m.**

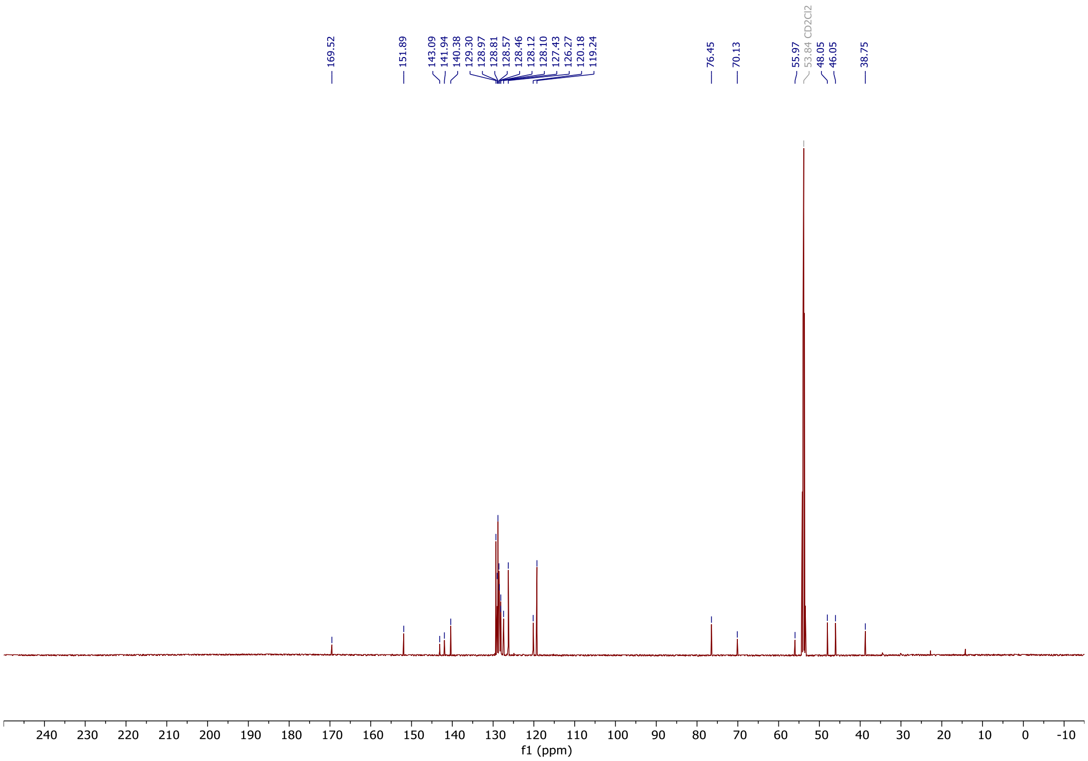

^{13}C-NMR spectrum of compound **3m.**

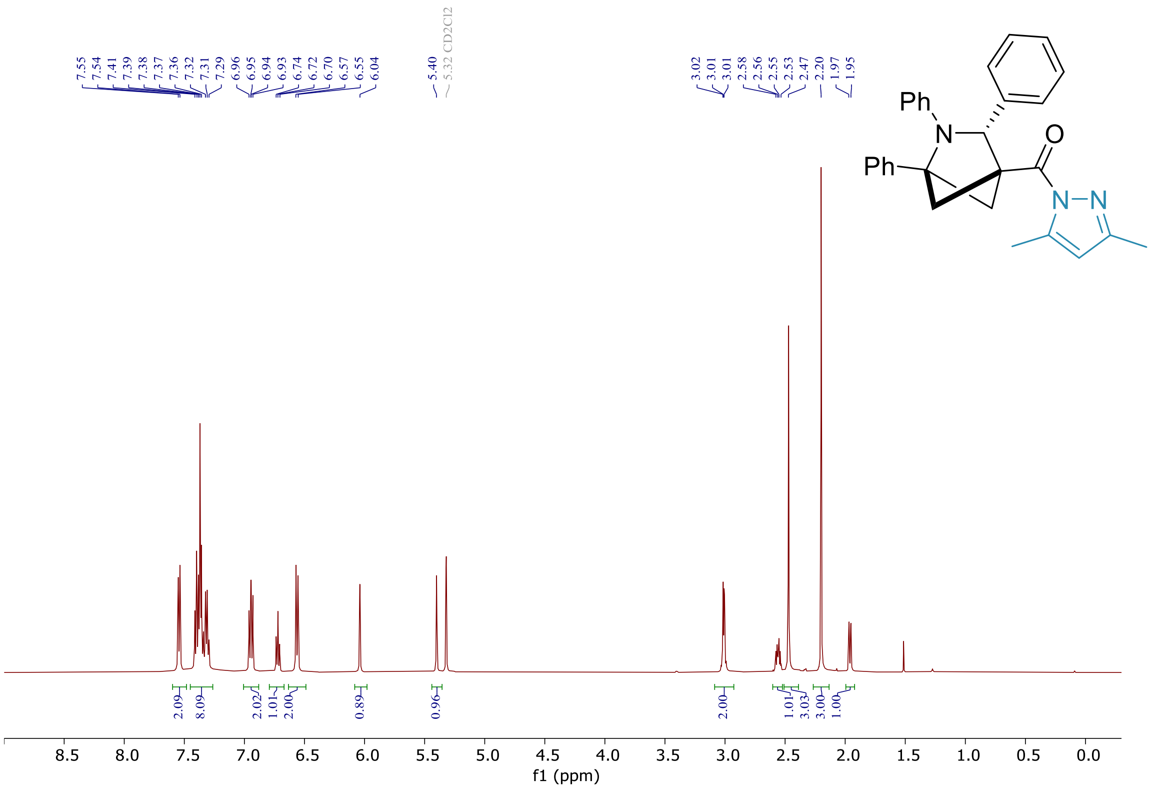

^{1}H-NMR spectrum of compound **3n.**

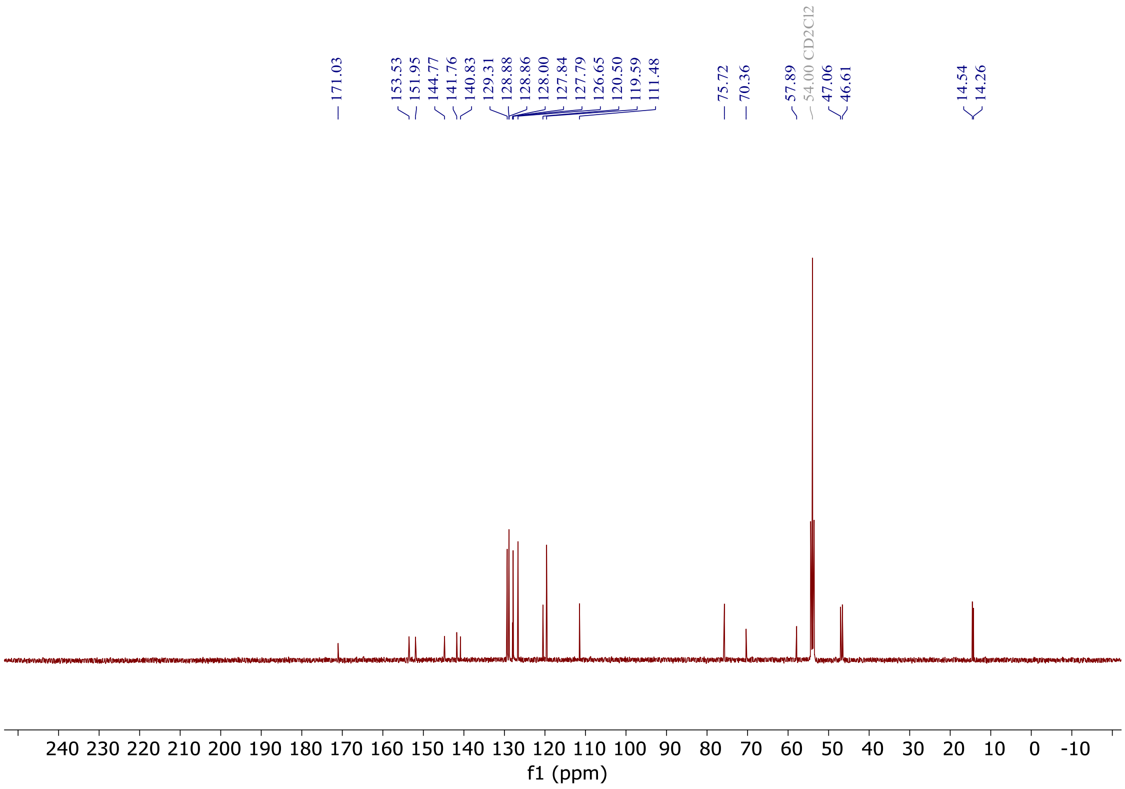

^{13}C-NMR spectrum of compound **3n.**

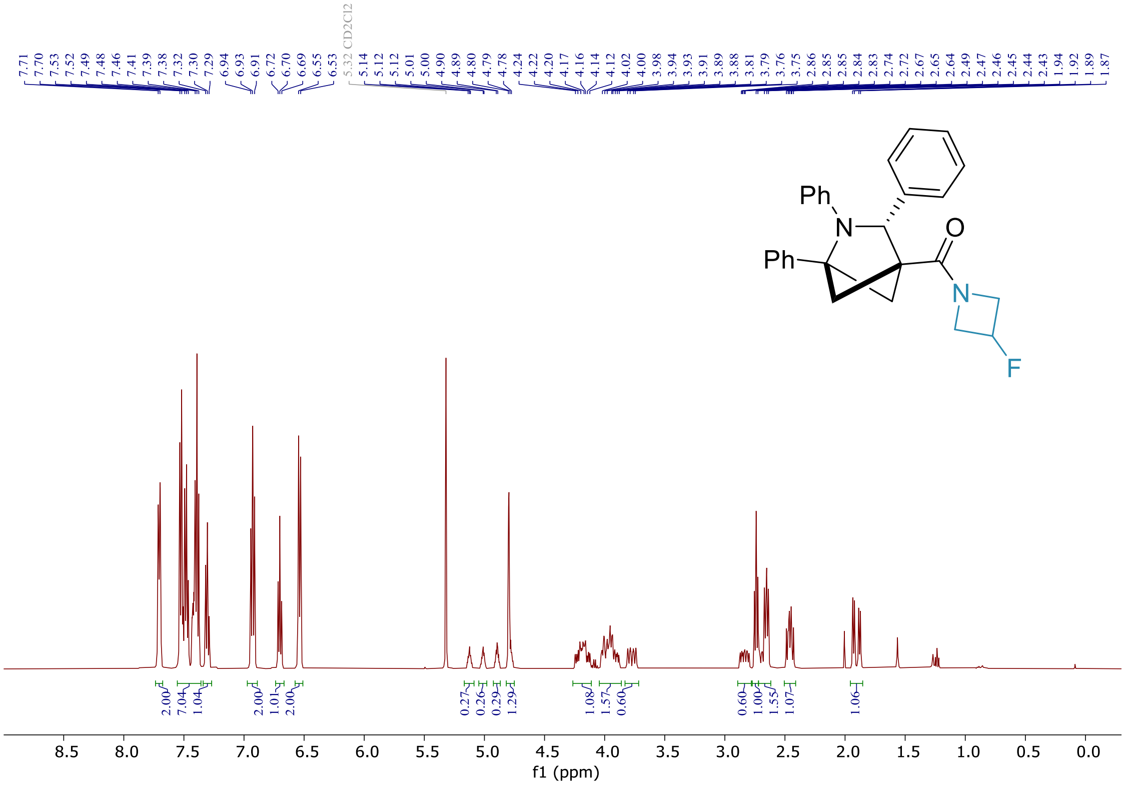

^{1}H-NMR spectrum of compound **3o.**

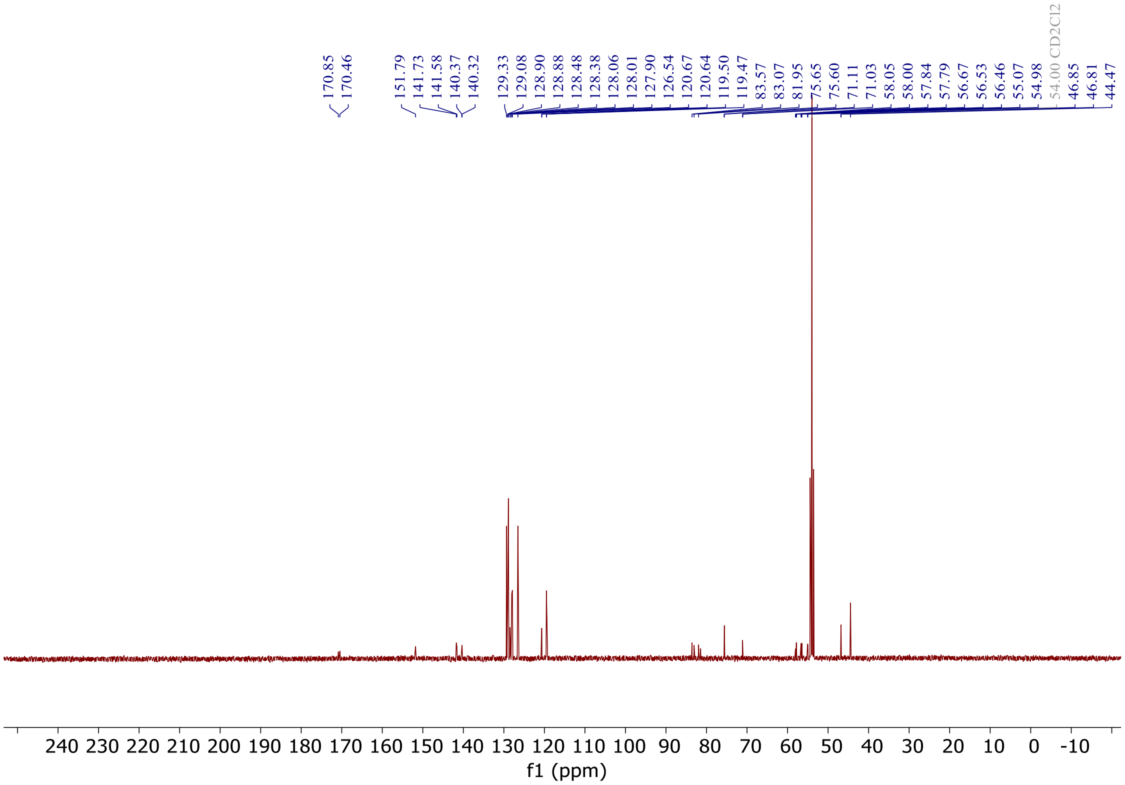

^{13}C-NMR spectrum of compound **3o.**

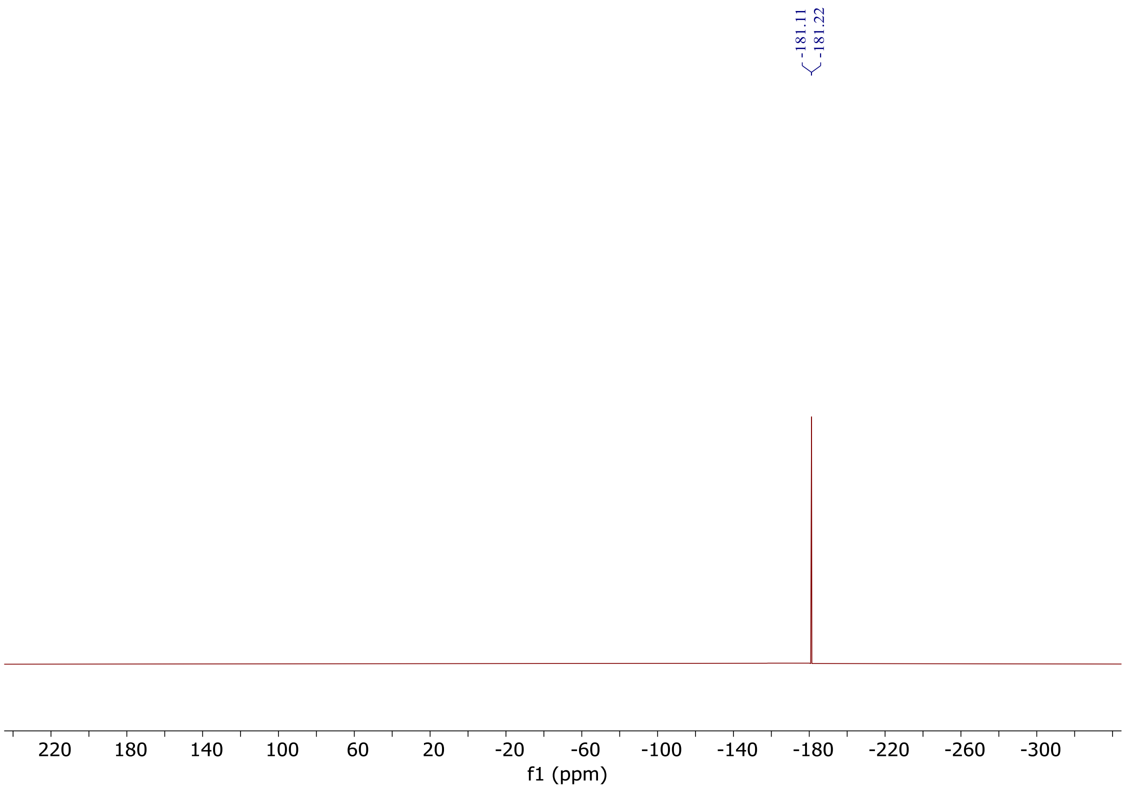

^{19}F-NMR spectrum of compound **3o.**

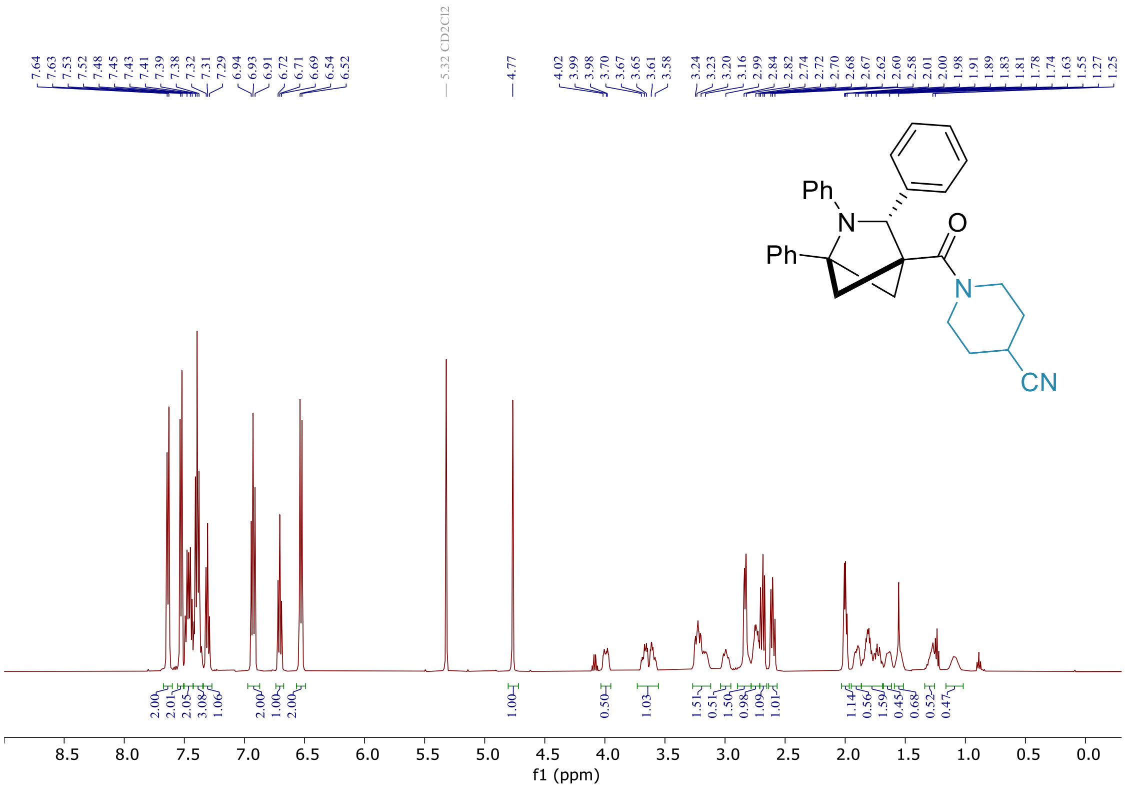

^{1}H-NMR spectrum of compound **3p.**

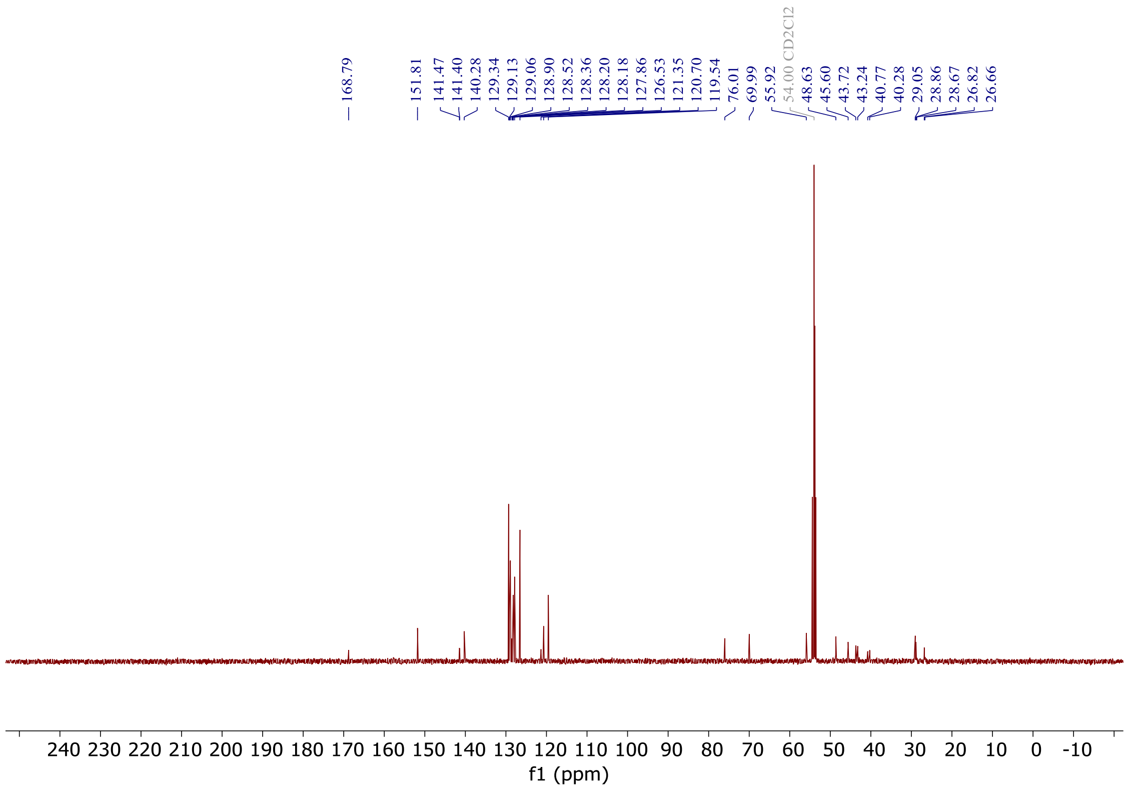

^{13}**C-NMR** spectrum of compound **3p.**

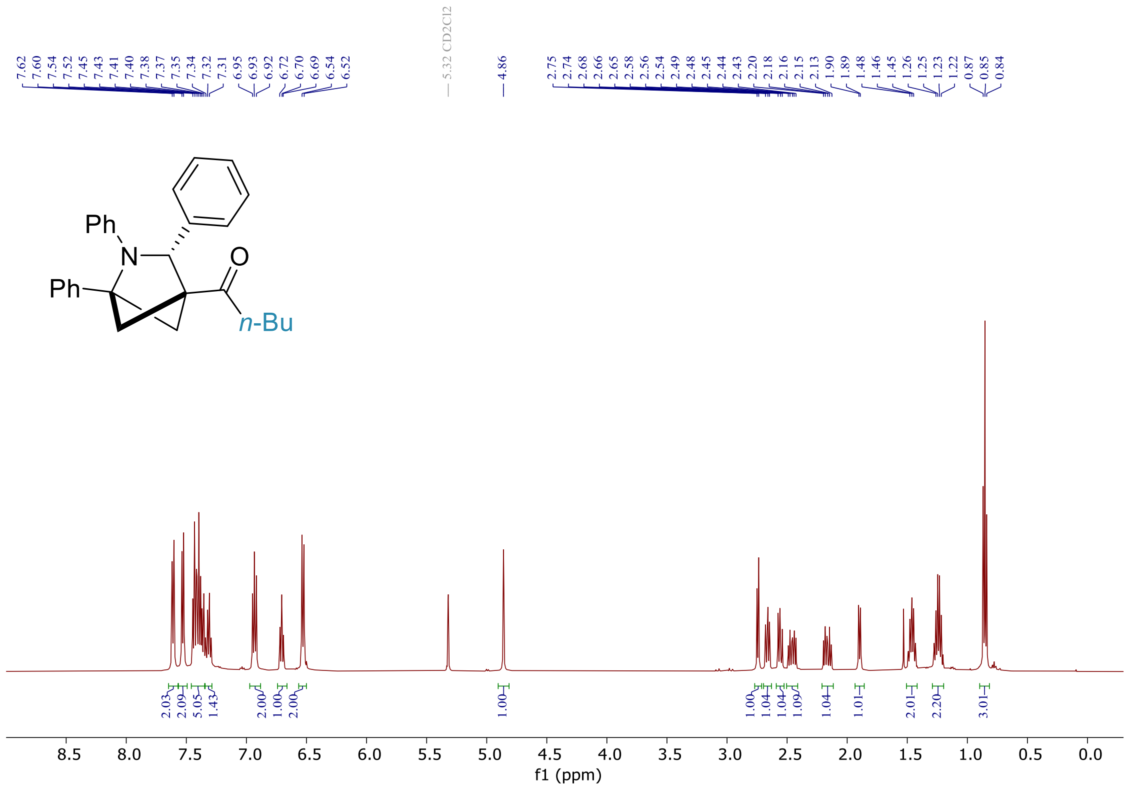

^{1}**H-NMR** spectrum of compound **3q.**

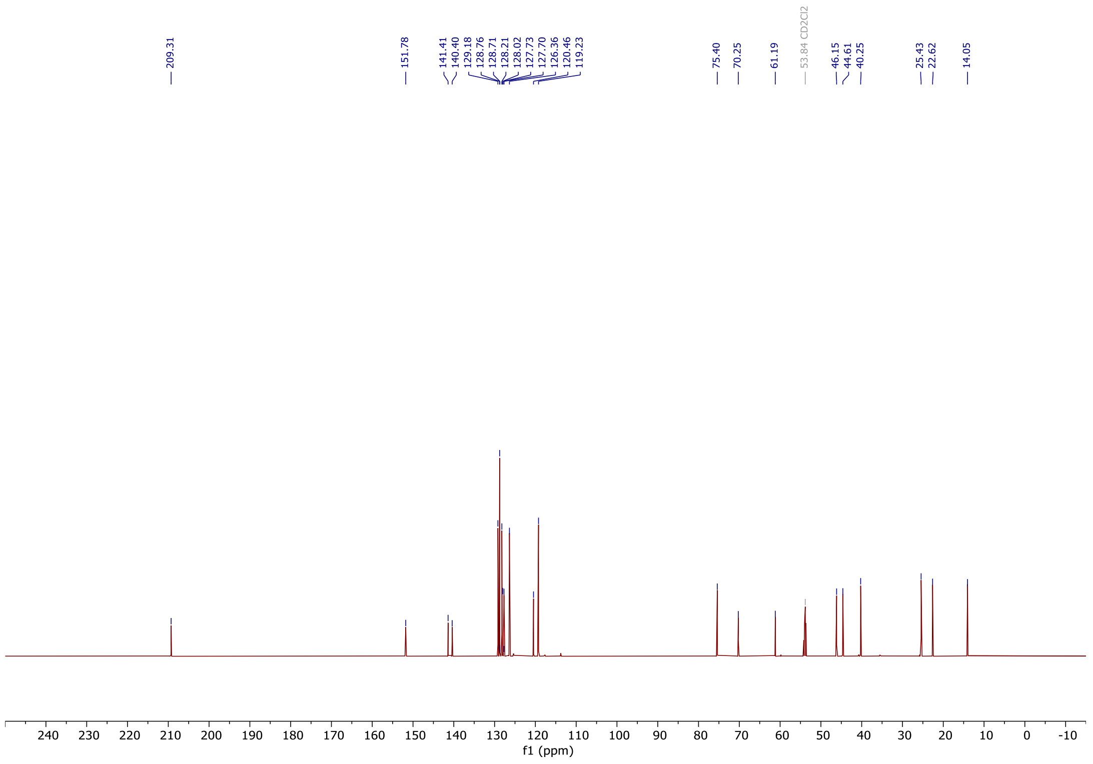

^{13}C-NMR spectrum of compound **3q.**

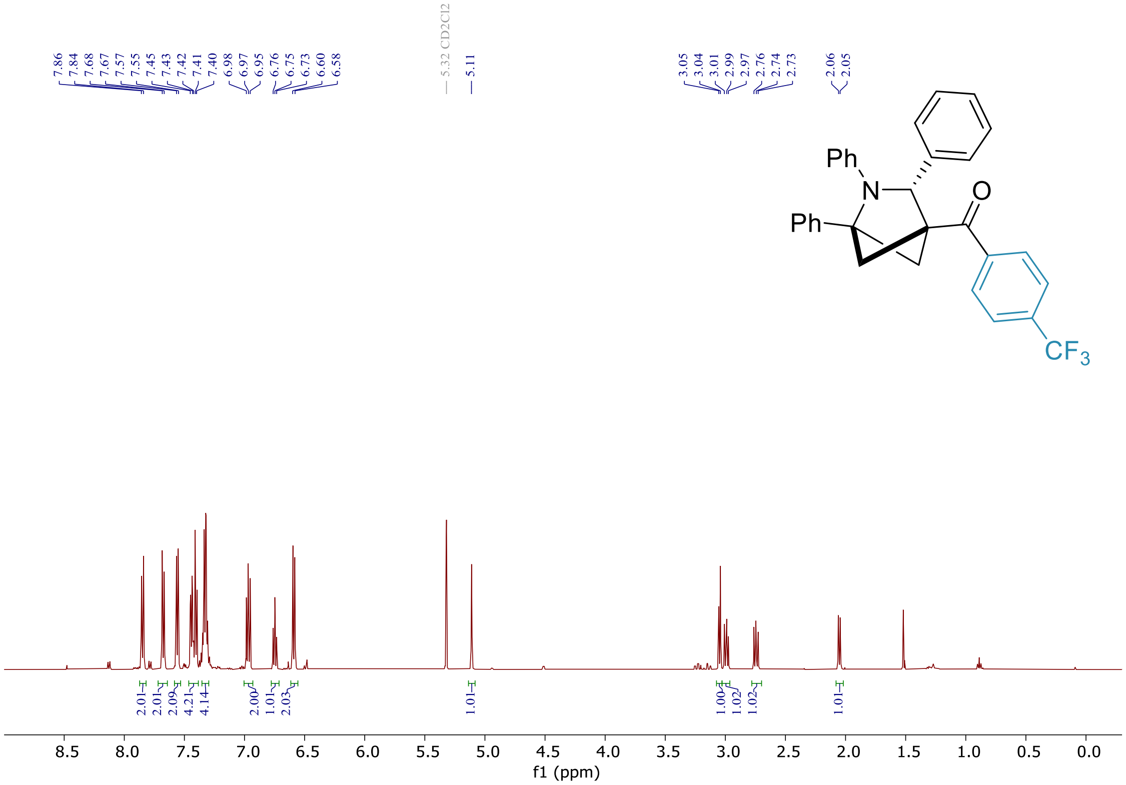

^{1}H-NMR spectrum of compound **3r.**

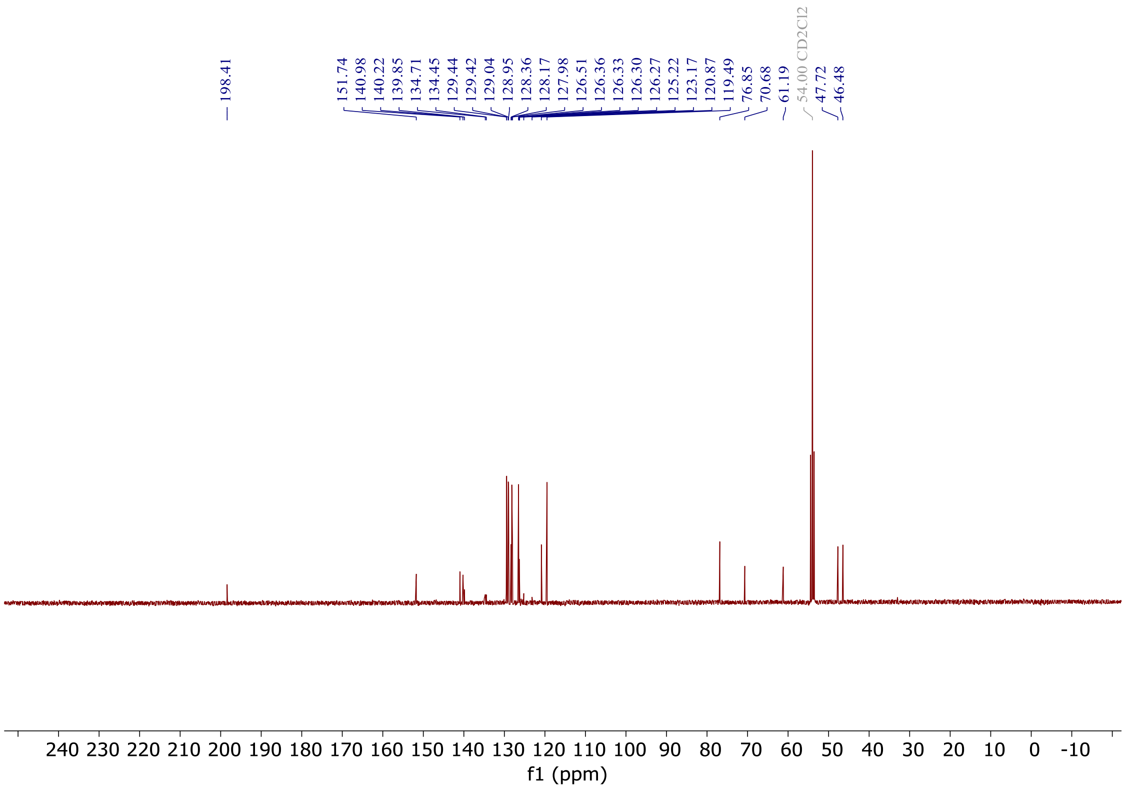

^{13}C-NMR spectrum of compound **3r.**

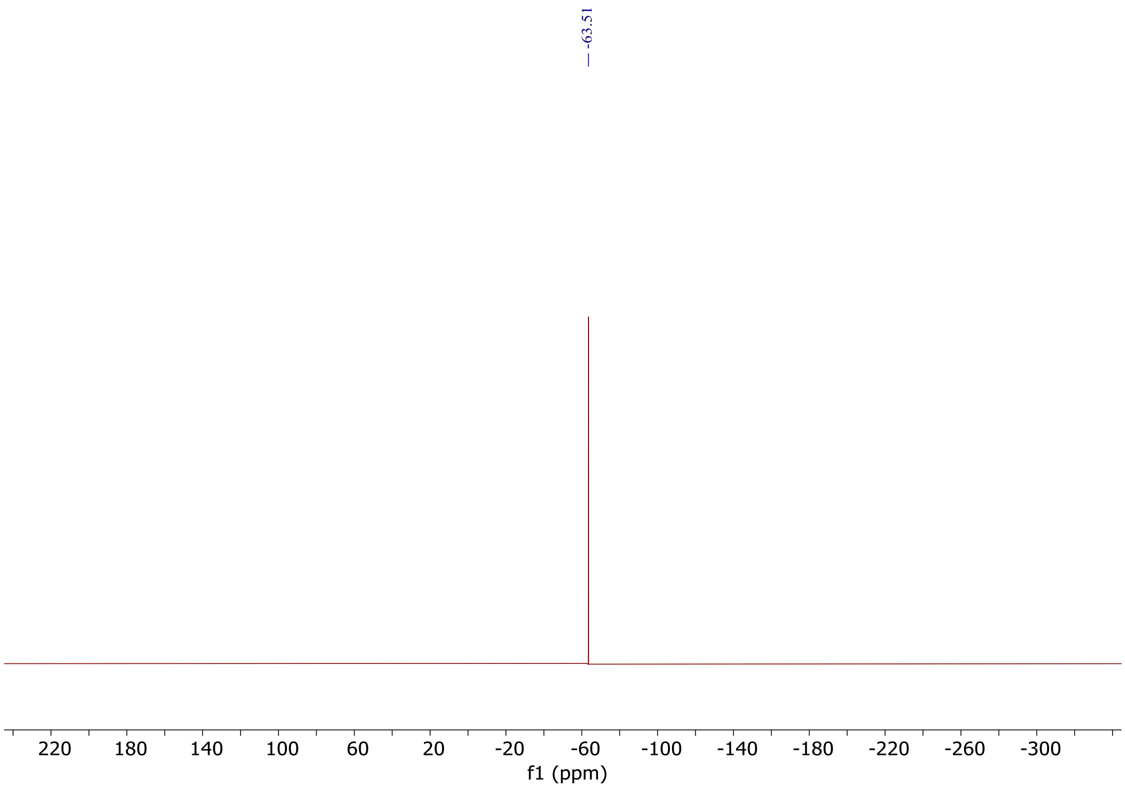

^{19}F-NMR spectrum of compound **3r.**

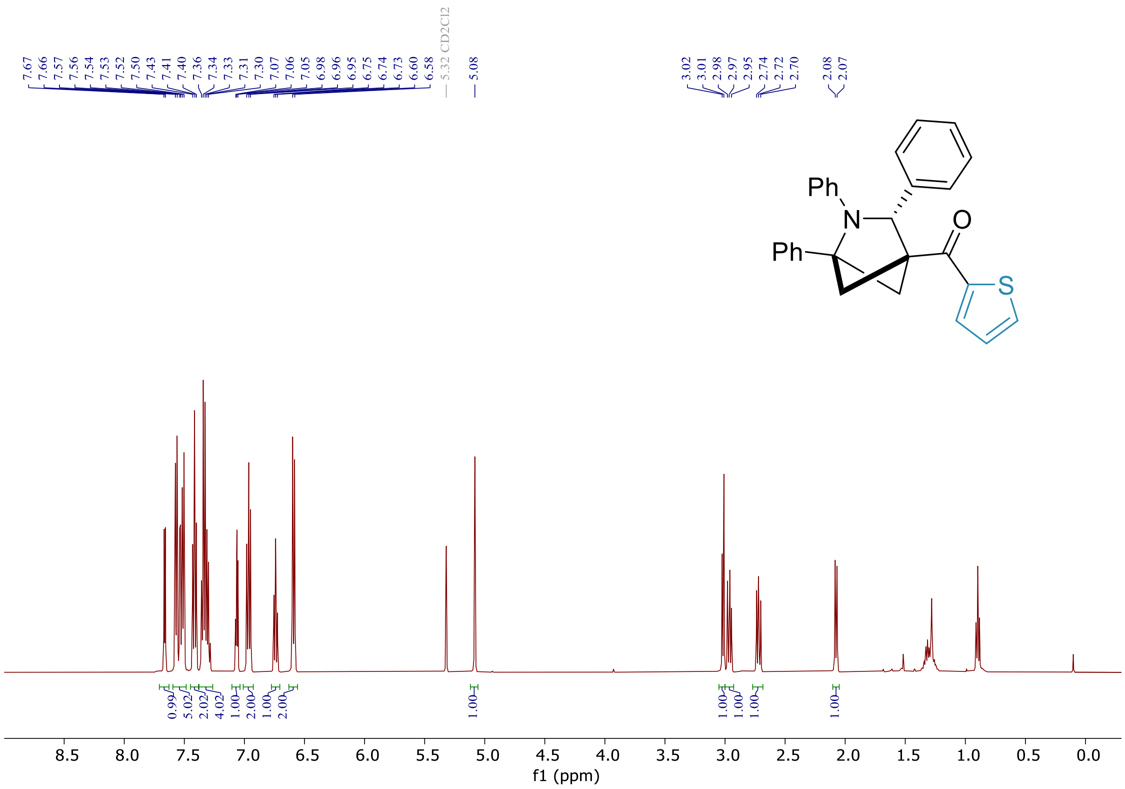

^{1}H-NMR spectrum of compound **3s.**

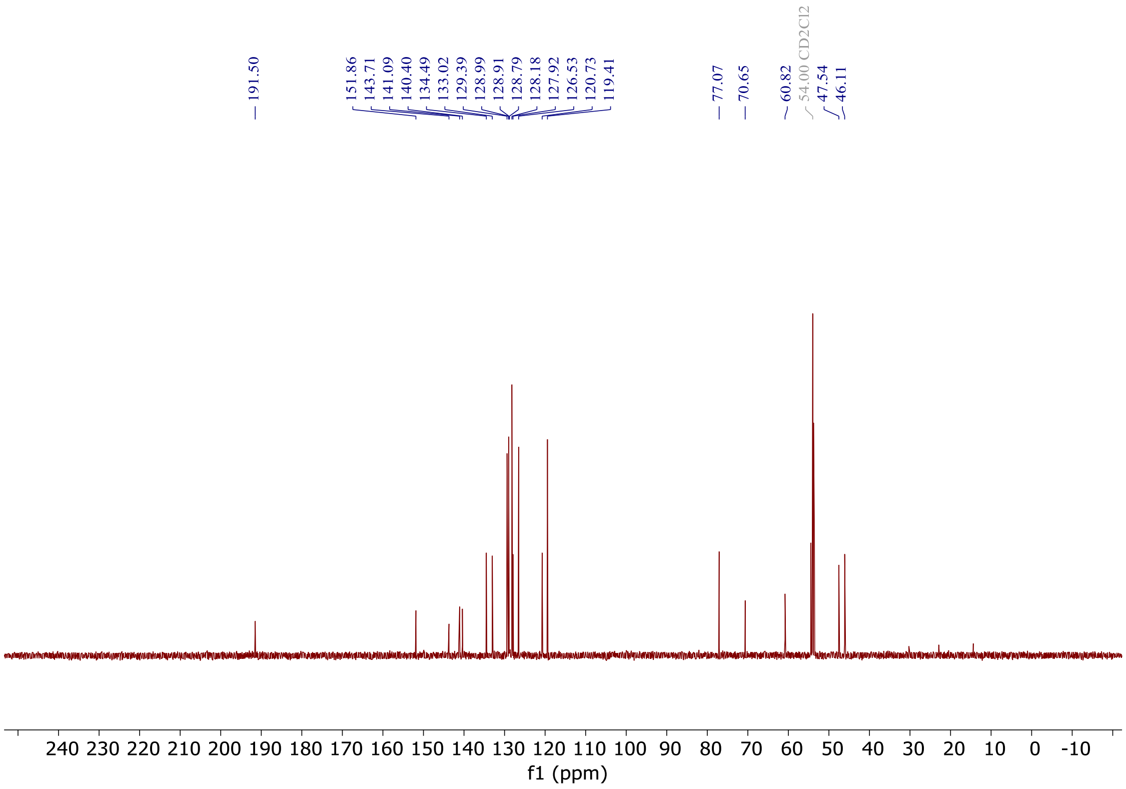

^{13}C-NMR spectrum of compound **3s.**

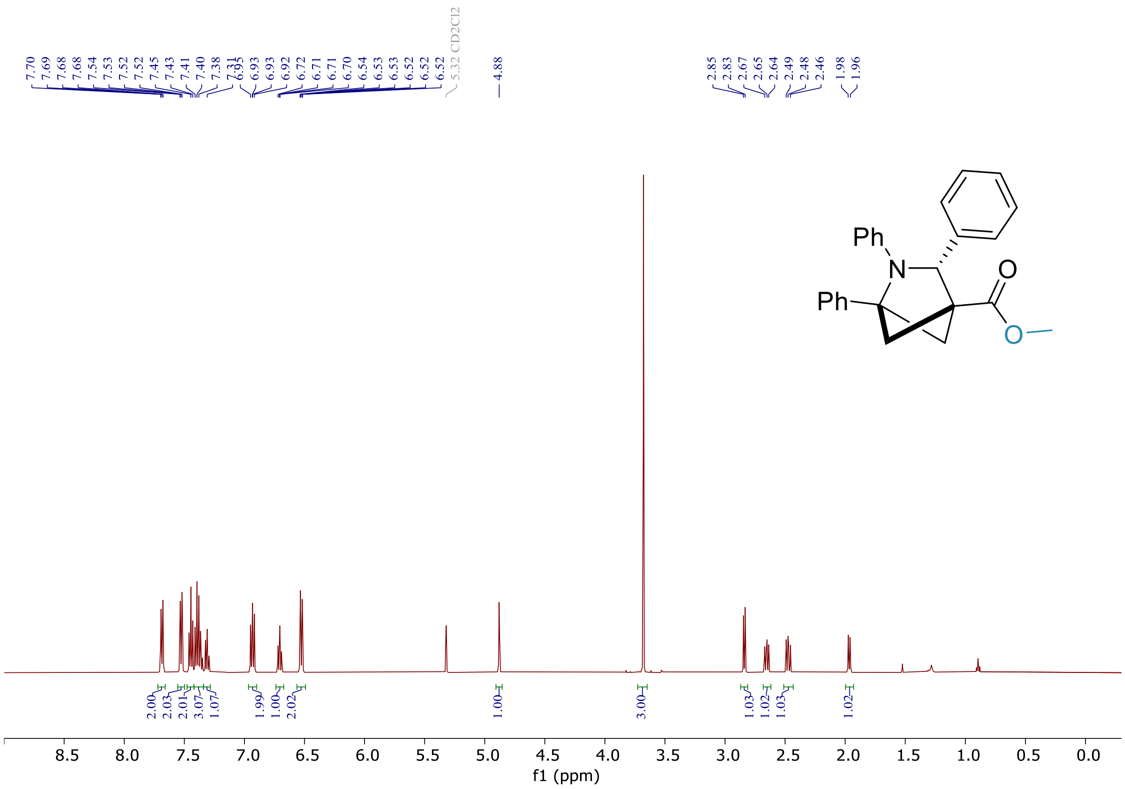

^{1}H-NMR spectrum of compound **3t.**

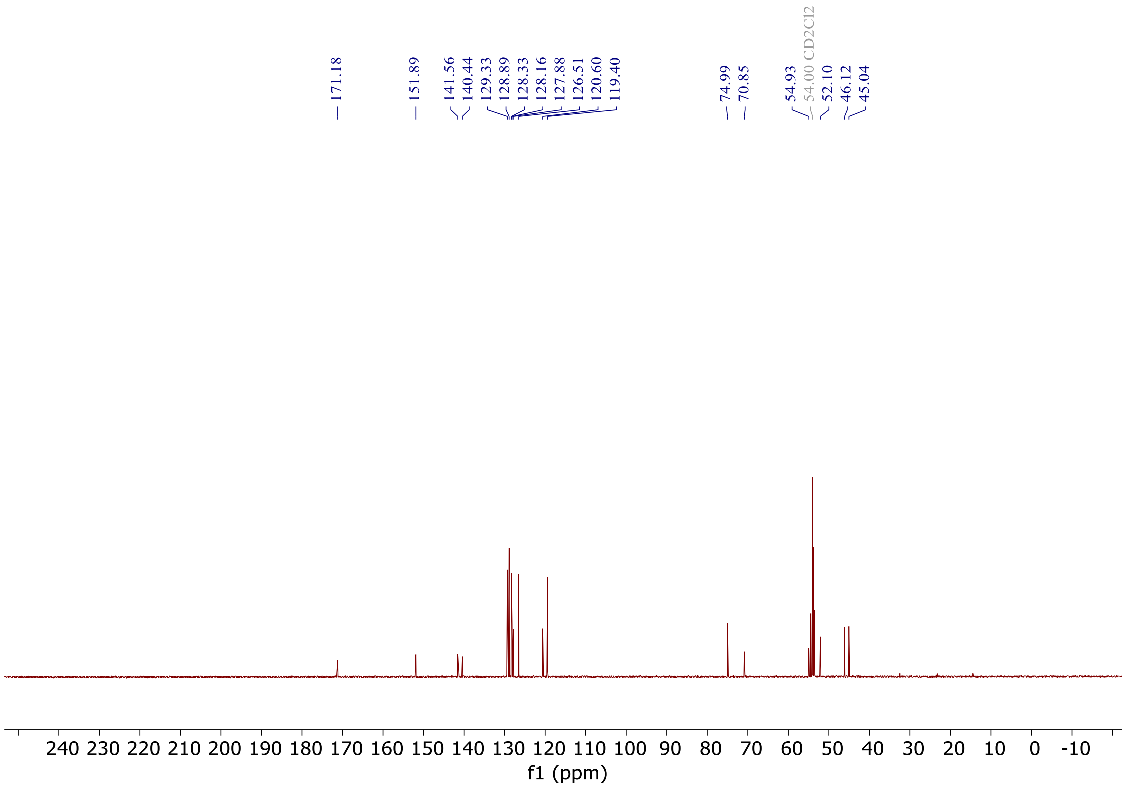

^{13}C-NMR spectrum of compound **3t.**

9 HPLC Chromatograms

HPLC traces of *rac*-**3a** and chiral **3a.**

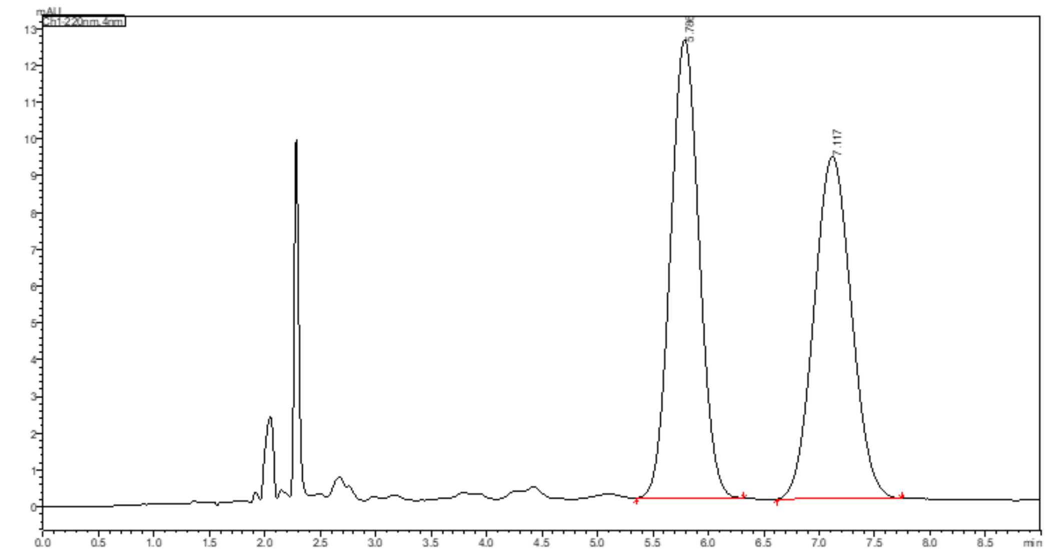

150 mm Chiralpak IK-3R, 4.6 mm i.D., 3µm, MeOH/water = 85/15, 1 ml/min, 298K, 220nm

Peak	Retention time (min)	Area (%)
1	5.8	49.2
2	7.1	50,8
Total		100.0

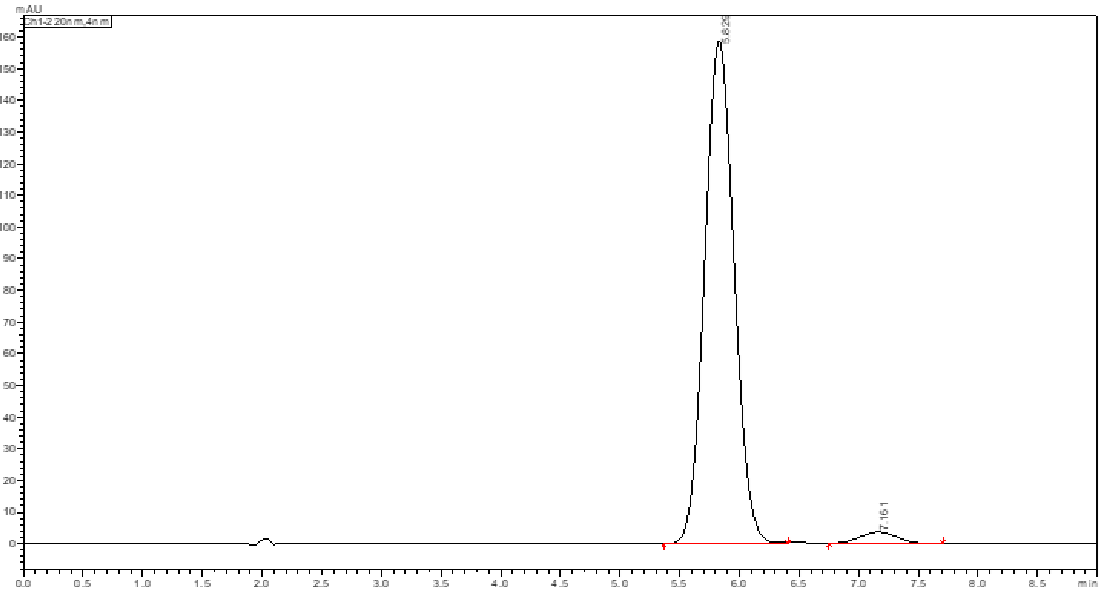

Peak	Retention time (min)	Area (%)
1	5.8	96.9
2	7.2	3.1
Total		100.0

HPLC traces of *rac*-**3b** and chiral **3b.**

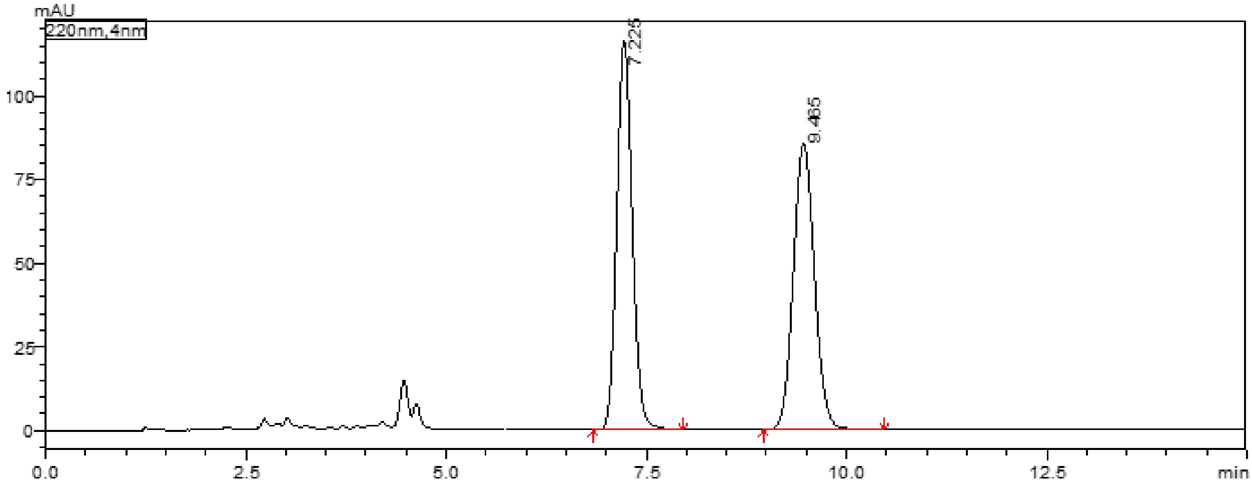

150 mm Chiralpak IC-3R, 4.6 mm i.D., 3μm, MeCN/water = 65/35, 1 ml/min, 298K, 220nm

Peak	Retention time (min)	Area (%)
1	7.2	50.1
2	9.5	49.9
Total		100.0

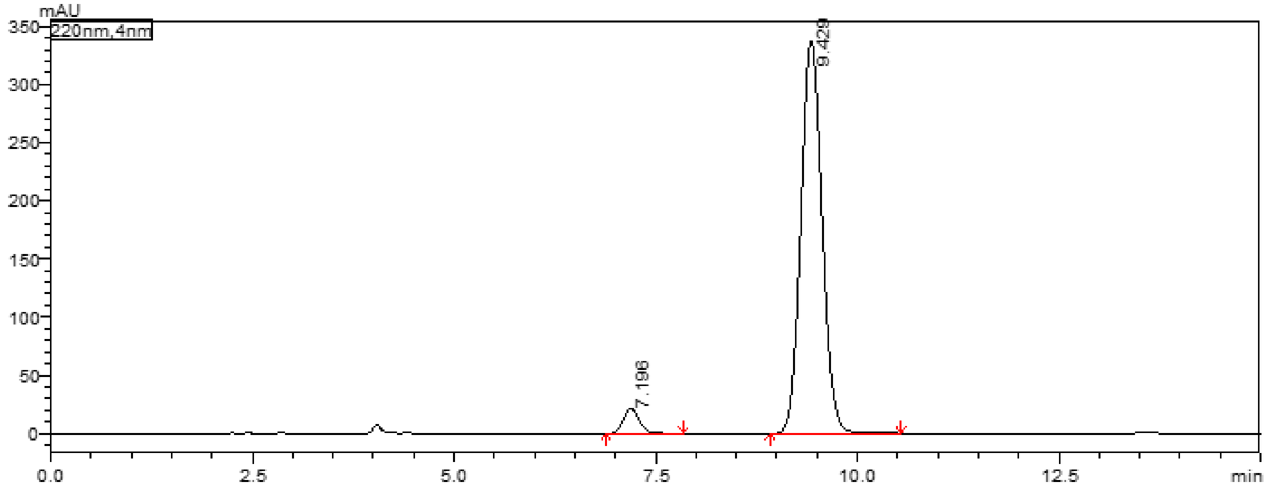

Peak	Retention time (min)	Area (%)
1	7.2	4.4
2	9.4	95.6
Total		100.0

HPLC traces of *rac*-**3c** and chiral **3c.**

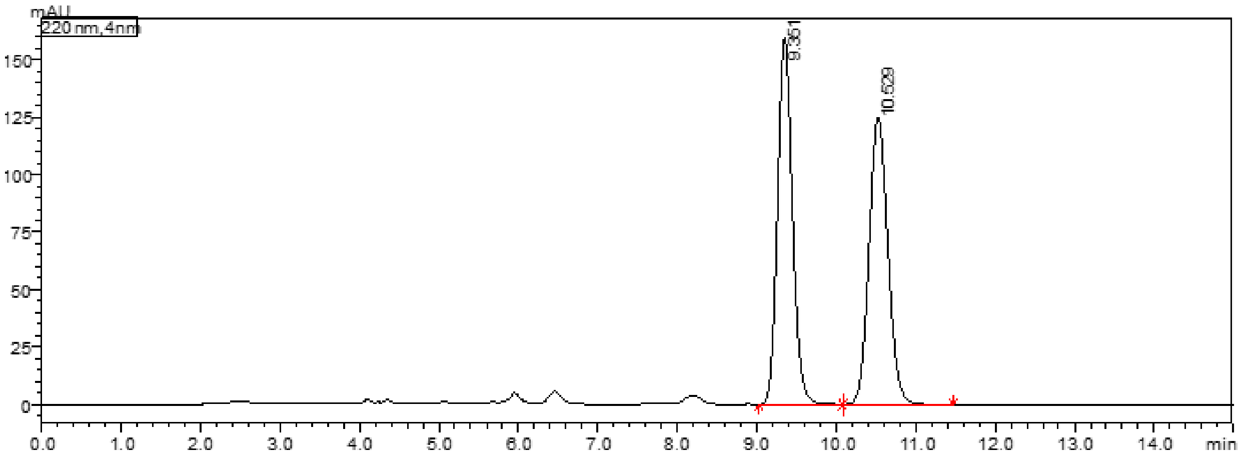

150 mm Chiralcel OJ-3R, 4.6 mm i.D., 3µm, MeCN/water = 55/45, 1 ml/min, 298K, 220nm

Peak	Retention time (min)	Area (%)
1	9.4	50.3
2	10.5	49.7
Total		100.0

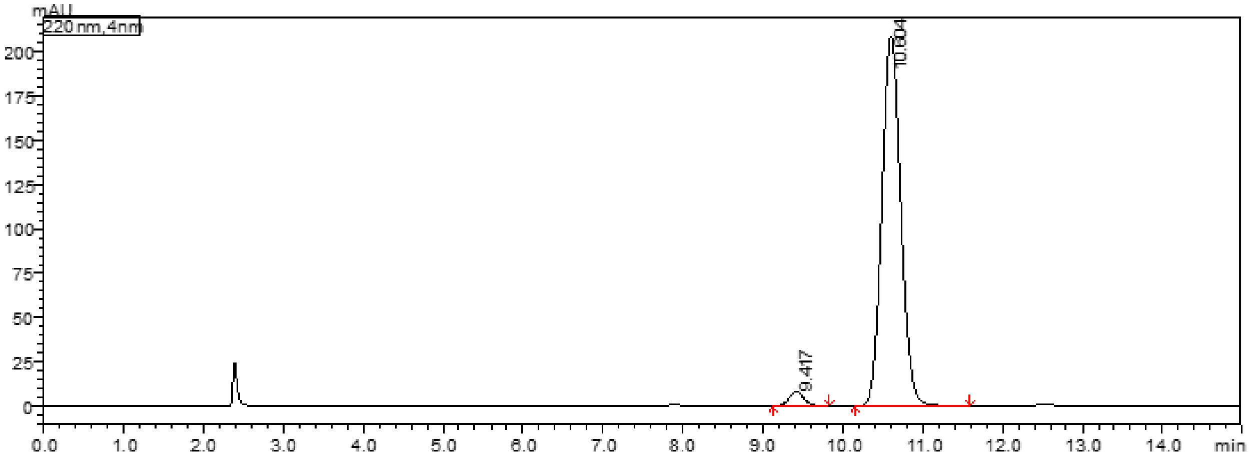

Peak	Retention time (min)	Area (%)
1	9.4	2.9
2	10.6	97.1
Total		100.0

HPLC traces of *rac*-**3d** and chiral **3d.**

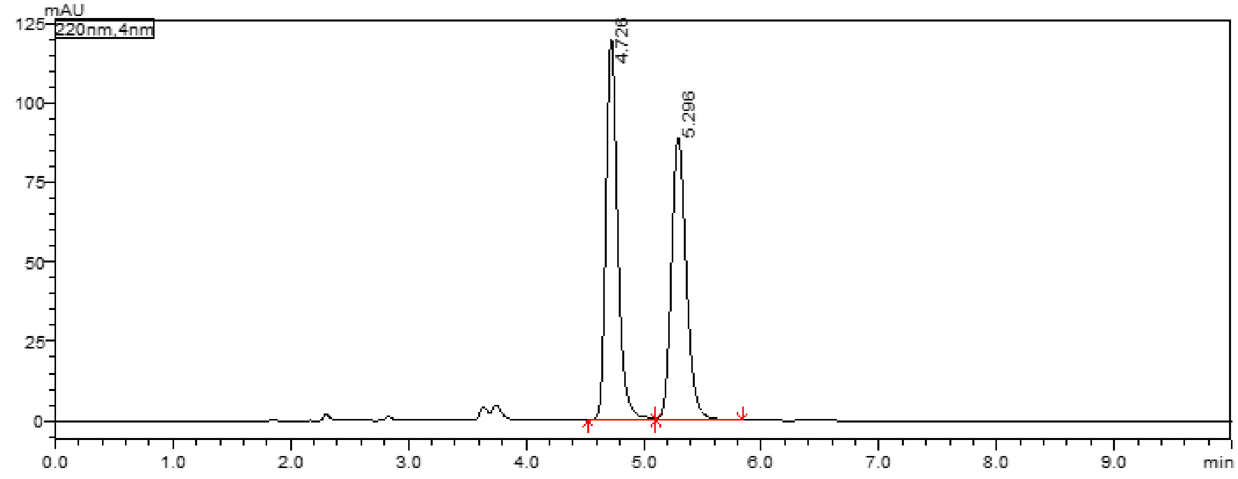

150 mm Chiralcel OJ-3R, 4.6 mm i.D., 3µm, MeCN/water = 70/30 1 ml/min, 298K, 220nm

Peak	Retention time (min)	Area (%)
1	4.7	51.6
2	5.3	49.4
Total		100.0

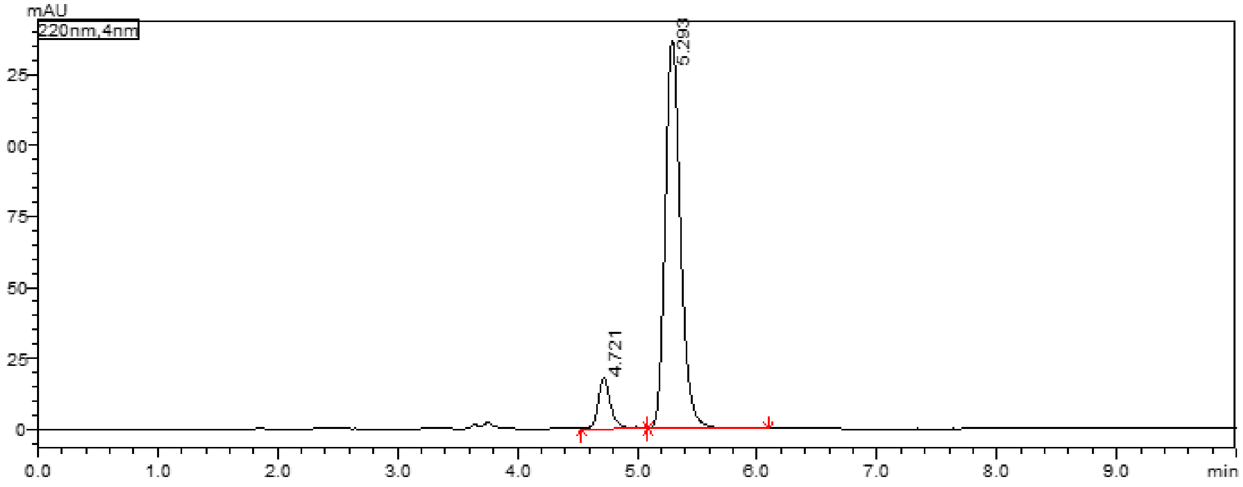

Peak	Retention time (min)	Area (%)
1	4.7	9.8
2	5.3	90.2
Total		100.0

HPLC traces of *rac*-**3e** and chiral **3e.**

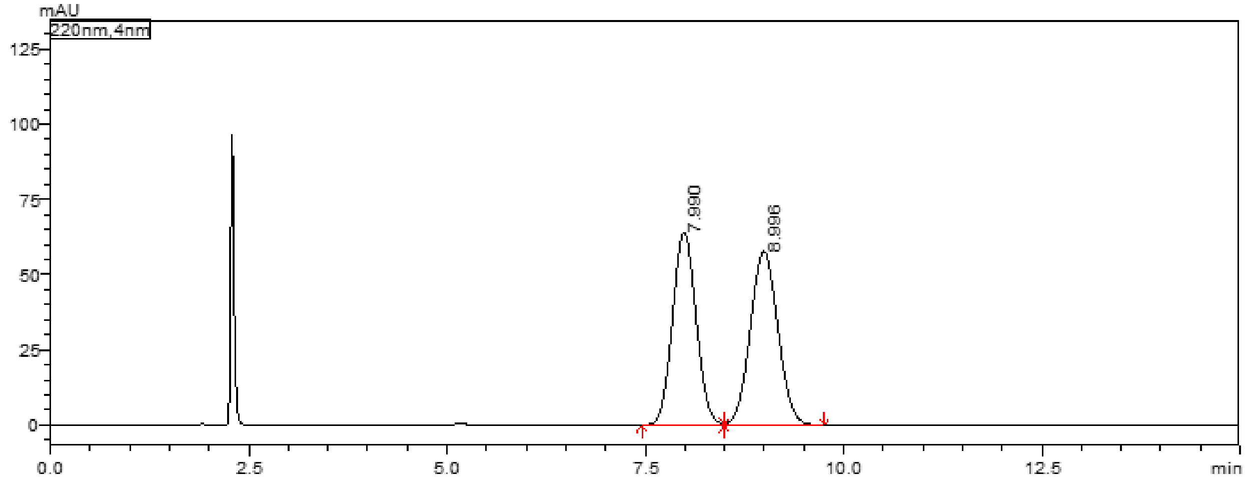

150 mm Chiralcel OZ-3R, 4.6 mm i.D., 3µm, MeCN/water = 65/35, 1 ml/min, 298K, 220nm

Peak	Retention time (min)	Area (%)
1	8.0	48.7
2	9.0	51.3
Total		100.0

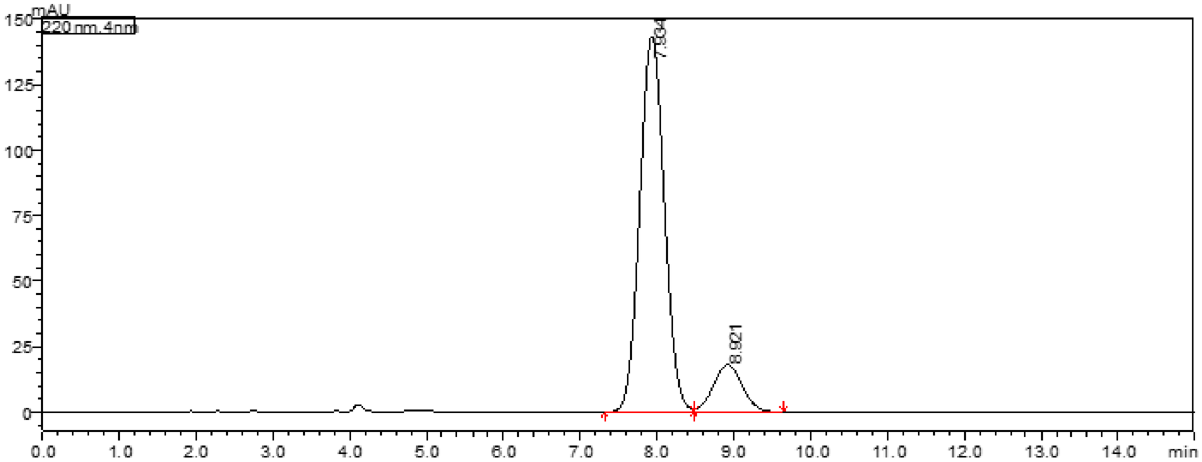

Peak	Retention time (min)	Area (%)
1	7.9	87.2
2	8.9	12.8
Total		100.0

HPLC traces of *rac*-**3f** and chiral **3f.**

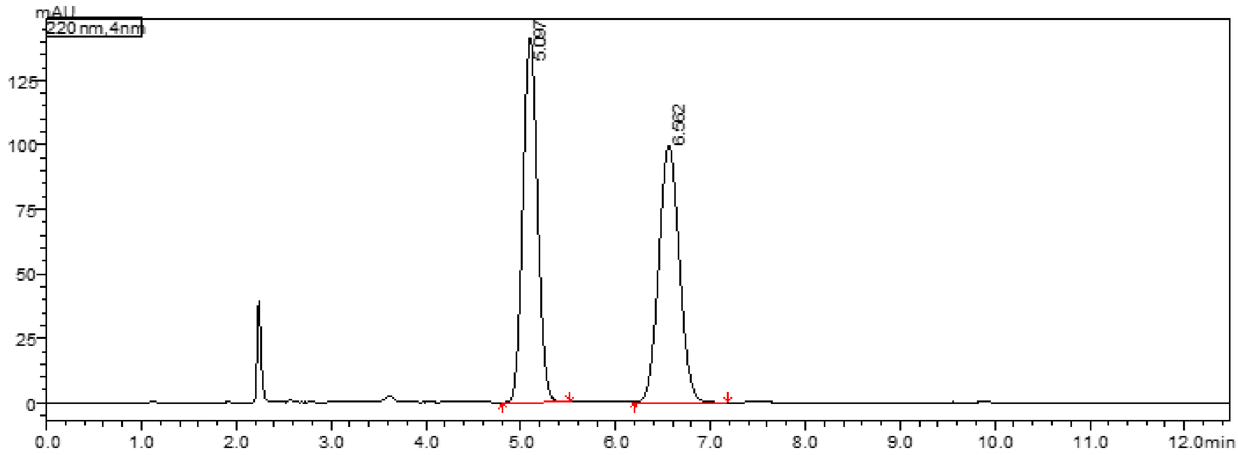

150 mm Chiralcel OZ-3R, 4.6 mm i.D., 3µm, MeCN/water = 70/30, 1 ml/min, 298K, 220nm

Peak	Retention time (min)	Area (%)
1	5.1	50.1
2	6.6	49.9
Total		100.0

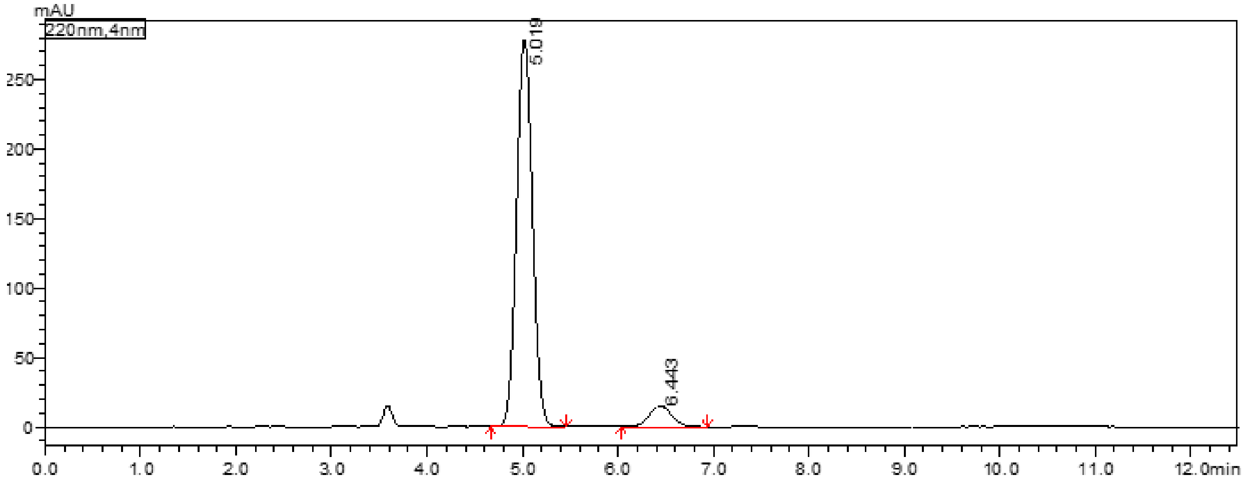

Peak	Retention time (min)	Area (%)
1	5.0	92.8
2	6.4	7.2
Total		100.0

HPLC traces of *rac*-**3g** and chiral **3g.**

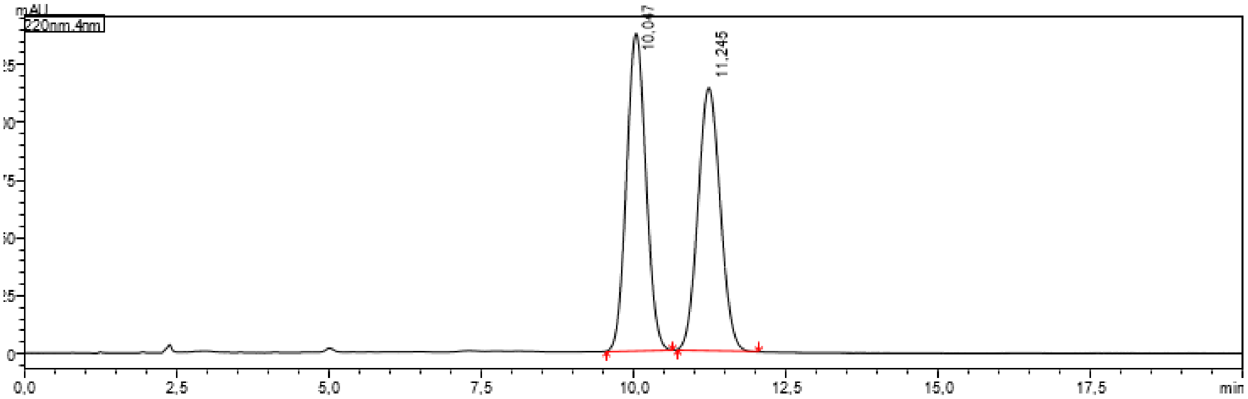

150 mm Chiralcel OZ-3R, 4.6 mm i.D., 3µm, MeCN/water = 60/40 1 ml/min, 298K, 220nm

Peak	Retention time (min)	Area (%)
1	10.0	51.3
2	11.2	48.7
Total		100.0

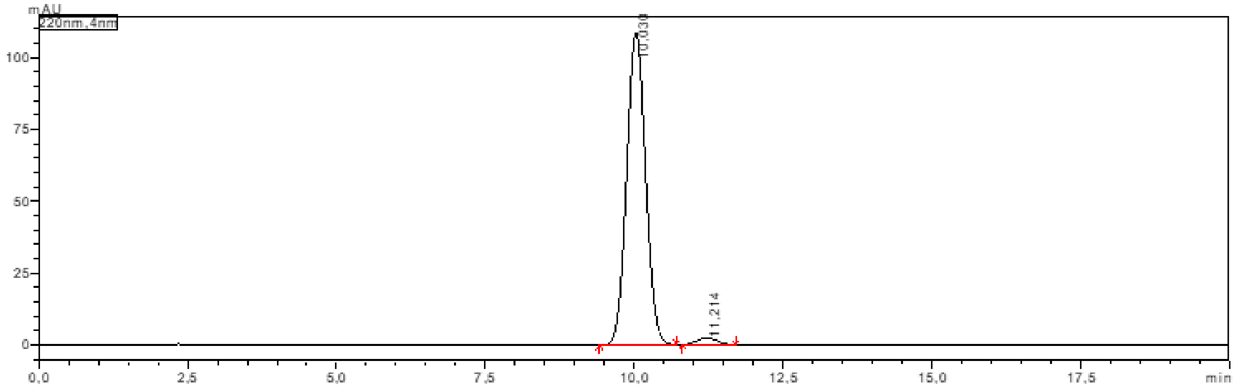

Peak	Retention time (min)	Area (%)
1	10.0	97.7
2	11.2	2.3
Total		100.0

HPLC traces of *rac*-**3h** and chiral **3h.**

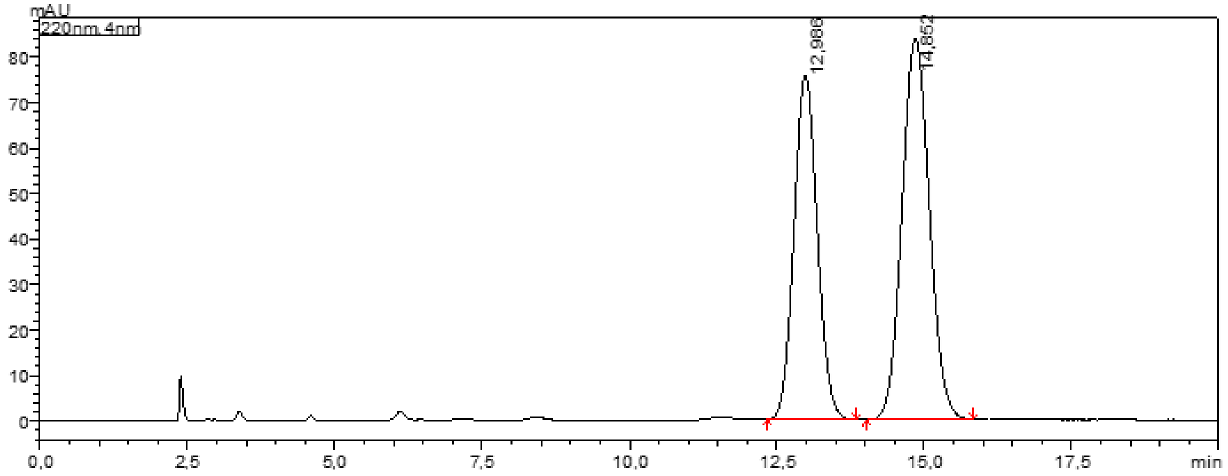

150 mm Chiralcel OZ-3R, 4.6 mm i.D., 3µm, MeCN/water = 60/40 1 ml/min, 298K, 220nm

Peak	Retention time (min)	Area (%)
1	12.9	43.5
2	14.9	56.5
Total		100.0

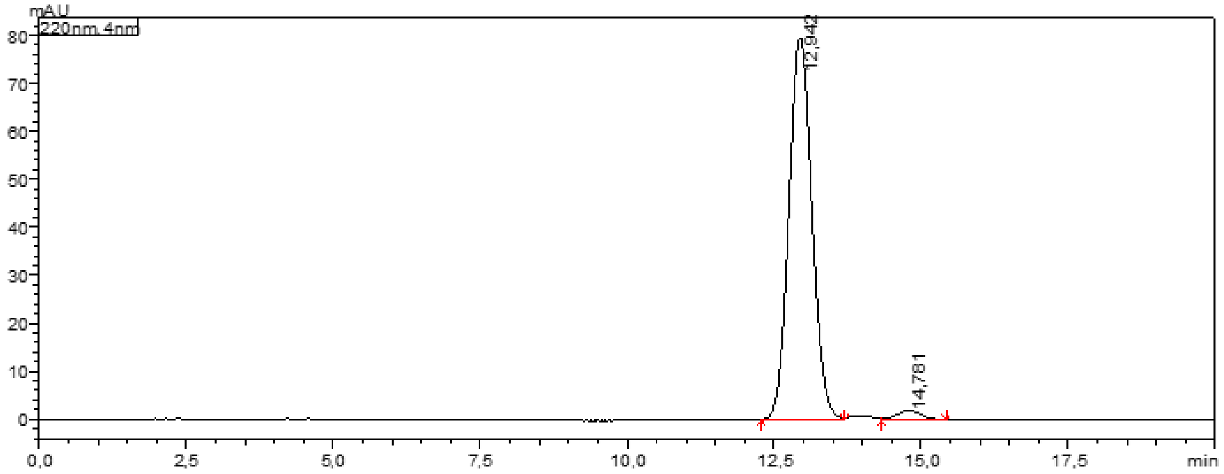

Peak	Retention time (min)	Area (%)
1	12.9	97.3
2	14.8	2.7
Total		100.0

HPLC traces of *rac*-**3i** and chiral **3i.**

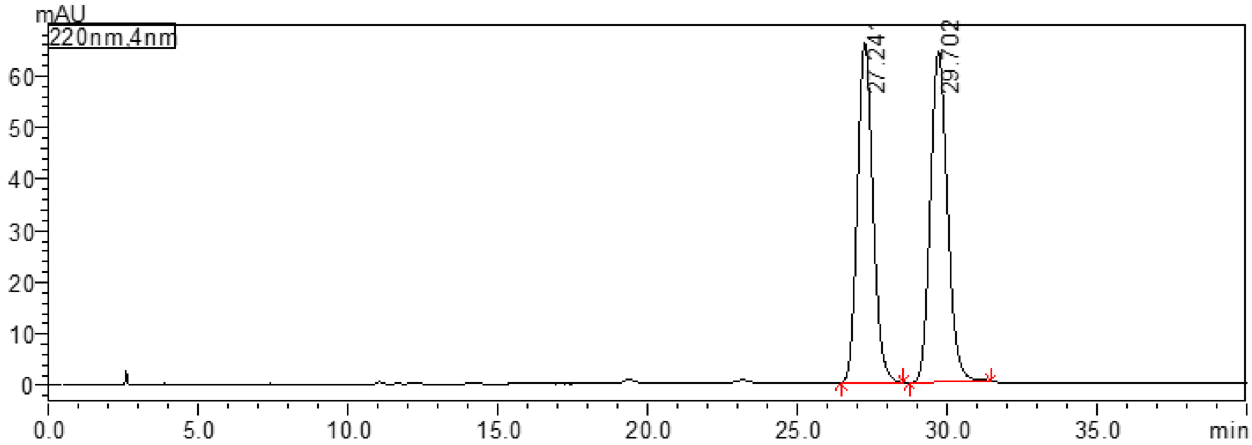

150 mm Chiralcel IB-3R, 4.6 mm i.D., 3μm, MeCN/water = 50/50 1 ml/min, 298K, 220nm

**Peak**	**Retention time (min)**	**Area (%)**
1	27.2	48.3
2	29.7	51.7
Total		100.0

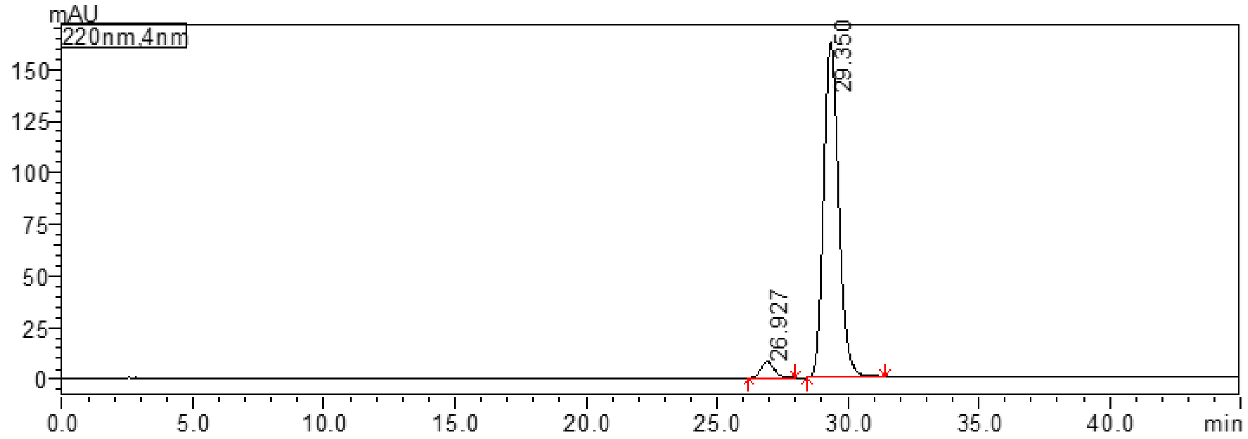

**Peak**	**Retention time (min)**	**Area (%)**
1	26.9	4.1
2	29.3	95.9
Total		100.0

HPLC traces of *rac*-**3j** and chiral **3j.**

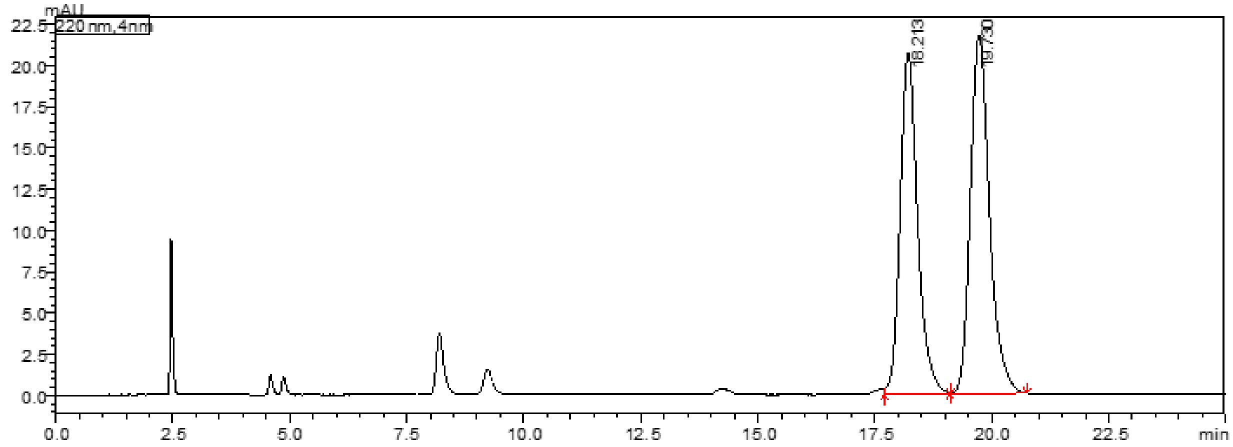

150 mm Chiralcel IBN-3R, 4.6 mm i.D., 3µm, MeCN/water = 55/45 1 ml/min, 298K, 220nm

Peak	Retention time (min)	Area (%)
1	18.2	46.8
2	19.7	53.2
Total		100.0

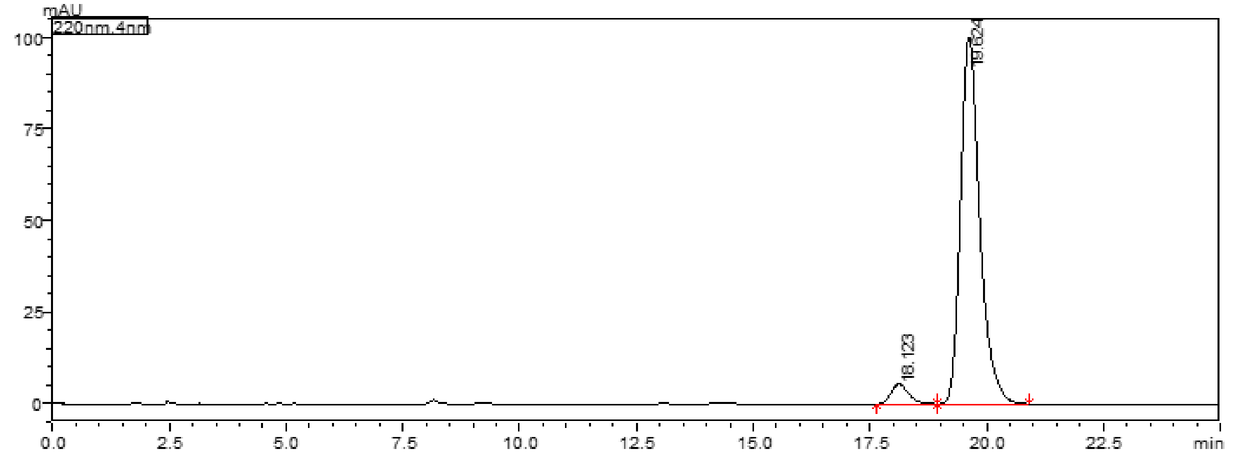

Peak	Retention time (min)	Area (%)
1	18.1	4.6
2	19.6	95.4
Total		100.0

HPLC traces of *rac*-**3k** and chiral **3k.**

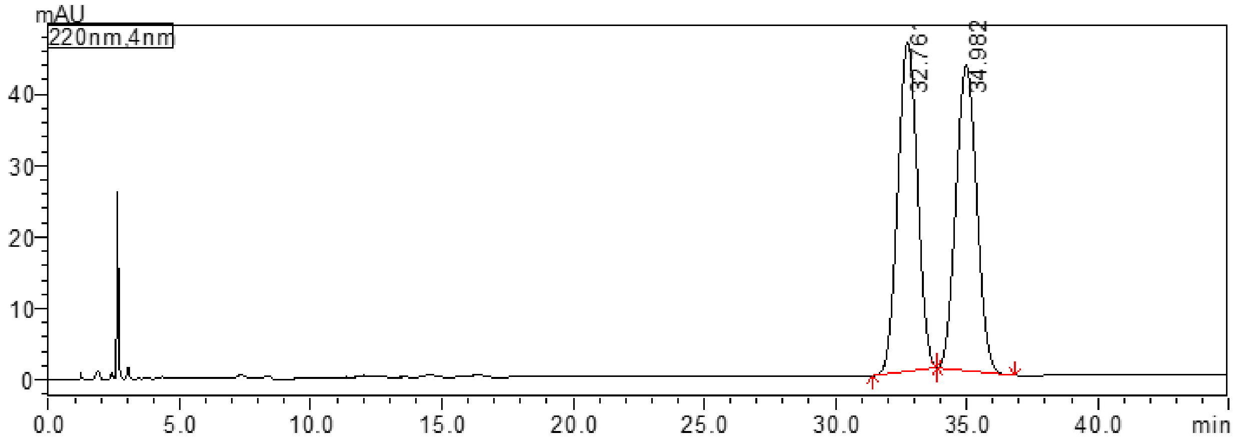

150 mm Chiralcel IC-3R, 4.6 mm i.D., 3µm, MeCN/water = 50/50 1 ml/min, 298K, 220nm

Peak	Retention time (min)	Area (%)
1	32.7	50.6
2	34.9	49.4
Total		100.0

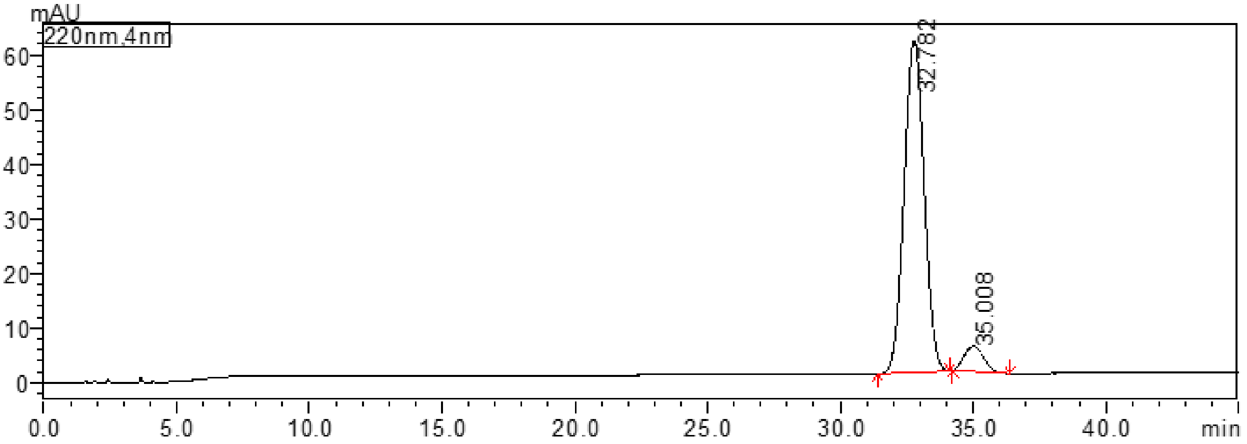

Peak	Retention time (min)	Area (%)
1	32.7	93.1
2	35.0	6.9
Total		100.0

HPLC traces of *rac*-**3l** and chiral **3l.**

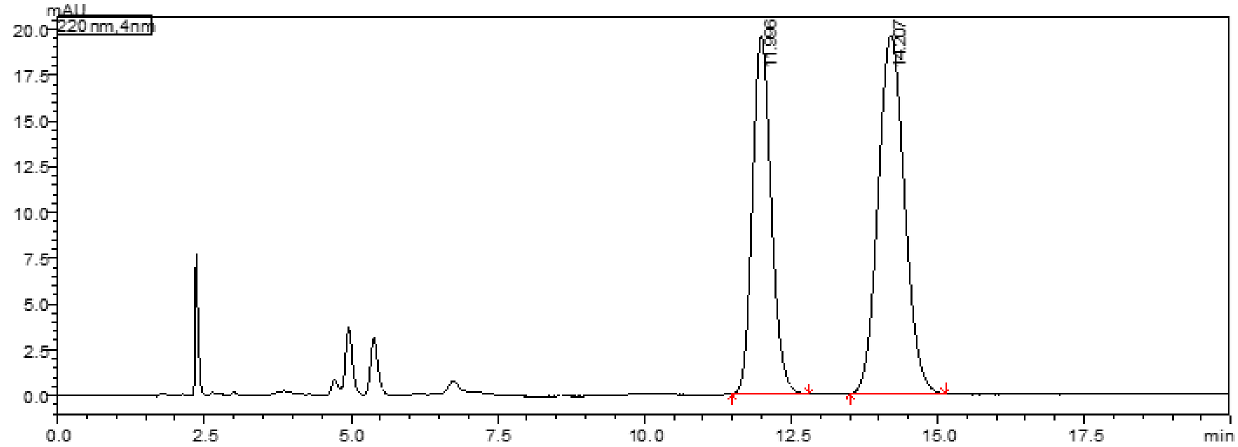

150 mm Chiralcel OZ-3R, 4.6 mm i.D., 3µm, MeCN/water = 65/35 1 ml/min, 298K, 220nm

Peak	Retention time (min)	Area (%)
1	12.0	41.5
2	14.2	58.5
Total		100.0

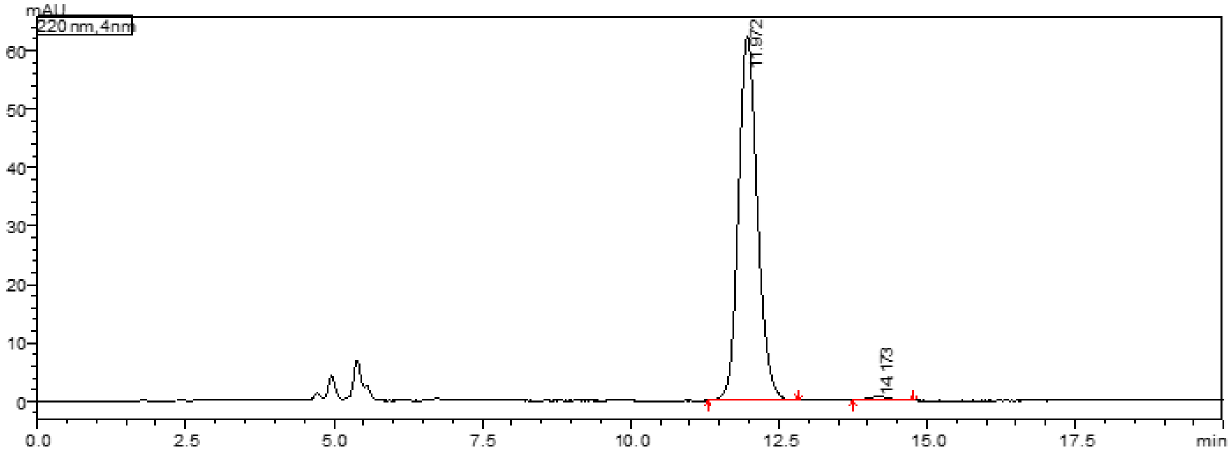

Peak	Retention time (min)	Area (%)
1	12.0	98.7
2	14.2	1.3
Total		100.0

HPLC traces of *rac*-**3m** and chiral **3m.**

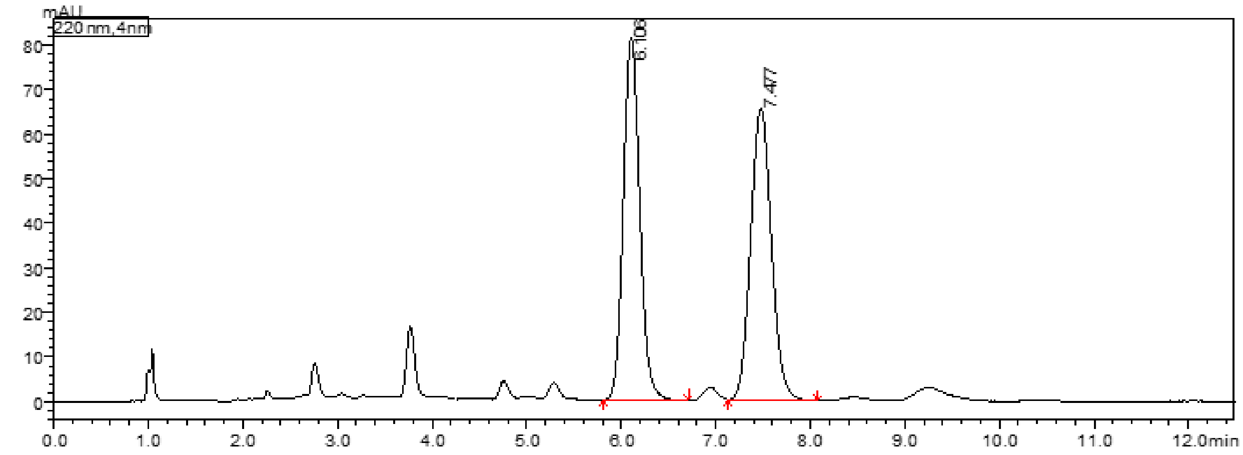

150 mm Chiralpak IC-3R, 4.6 mm i.D., 3μm, MeCN/water = 80/20, 1 ml/min, 298K, 220nm

Peak	Retention time (min)	Area (%)
1	6.1	49.6
2	7.5	50.4
Total		100.0

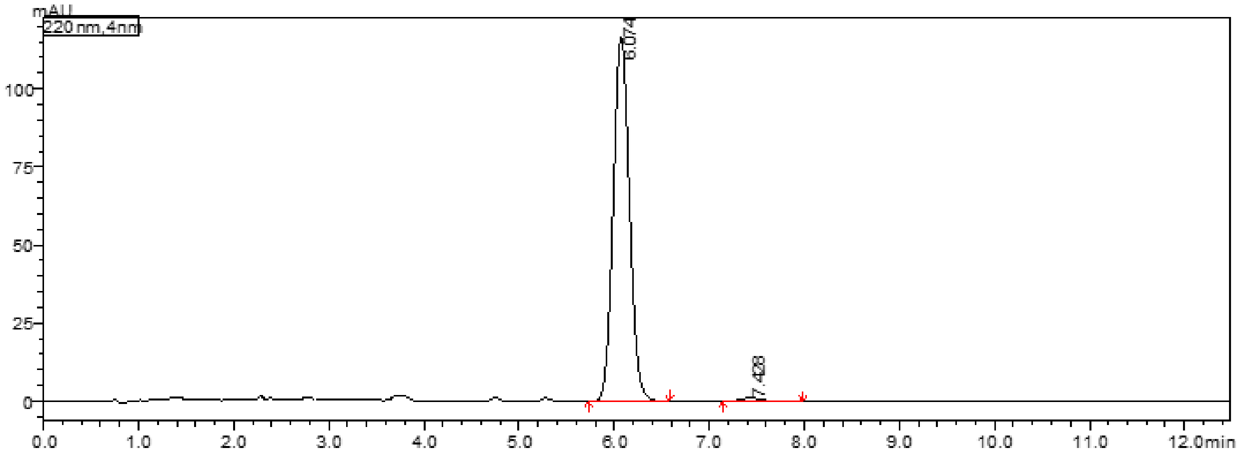

Peak	Retention time (min)	Area (%)
1	6.1	98.7
2	7.4	1.3
Total		100.0

HPLC traces of *rac*-**3n** and chiral **3n.**

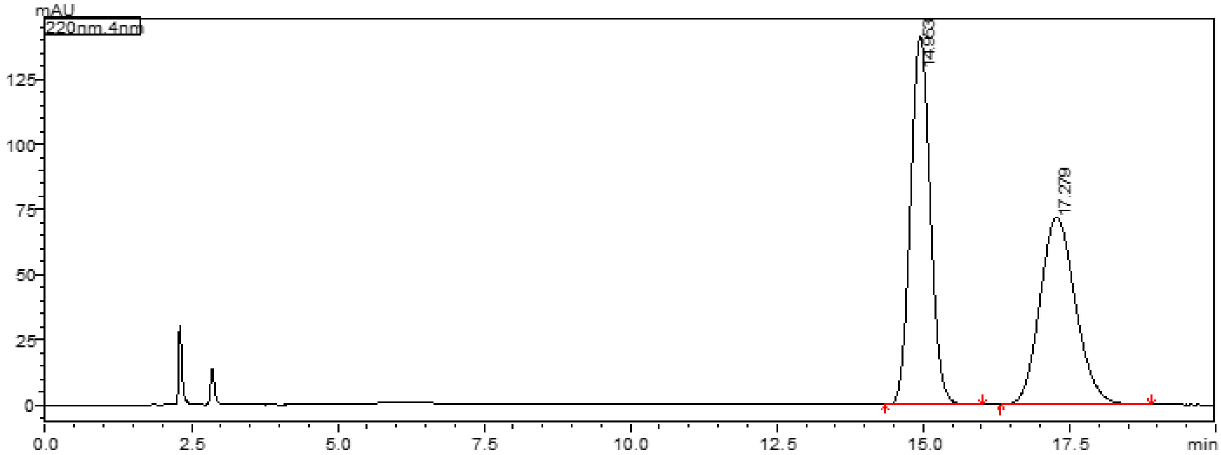

150 mm Chiralcel OJ-3R, 4.6 mm i.D., 3µm, MeCN/water = 60/40, 1 ml/min, 298K, 220nm

Peak	Retention time (min)	Area (%)
1	15.0	52.2
2	17.3	47.8
Total		100.0

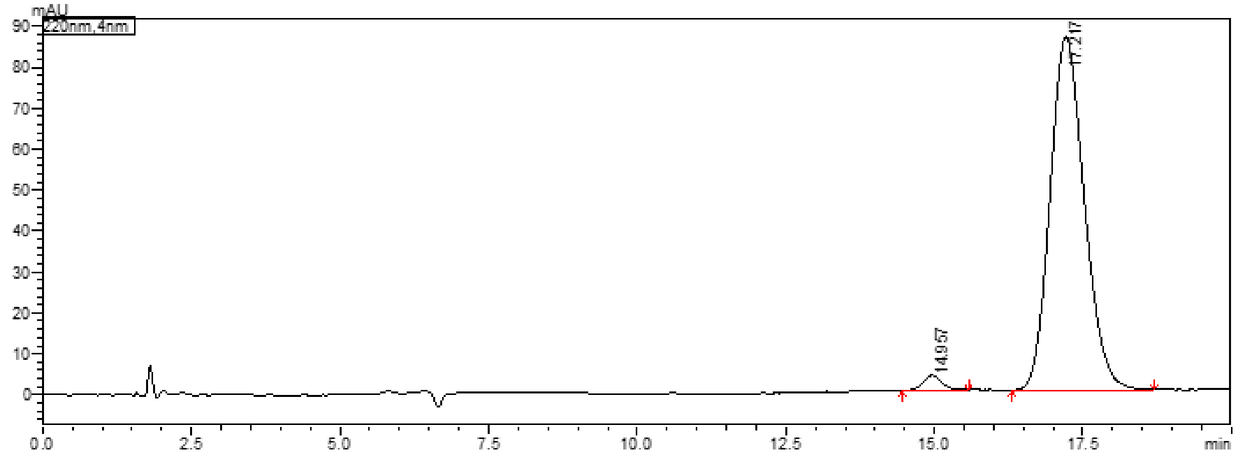

Peak	Retention time (min)	Area (%)
1	15.0	2.4
2	17.2	97.6
Total		100.0

HPLC traces of *rac*-**3o** and chiral **3o.**

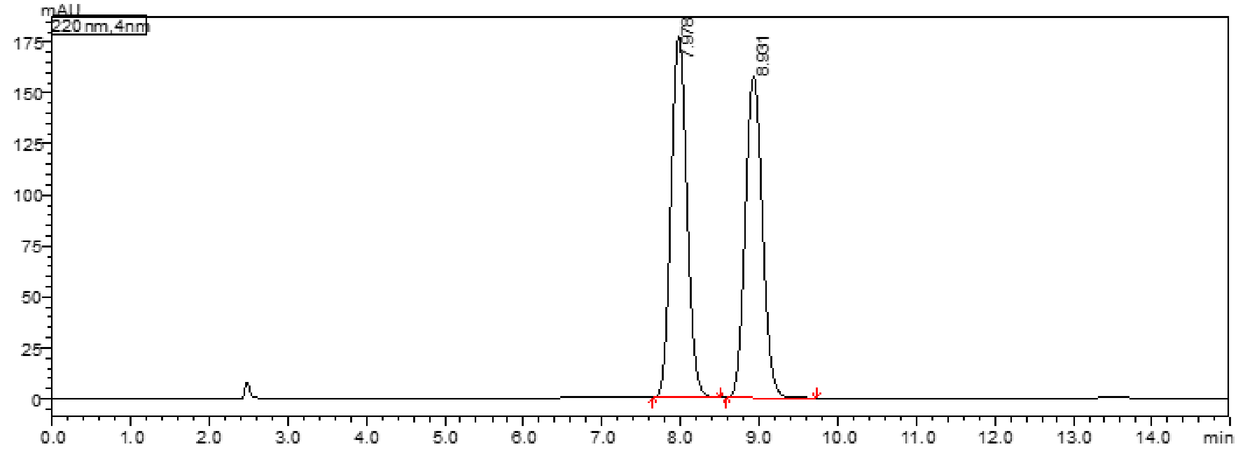

150 mm Chiralcel OJ-3R, 4.6 mm i.D., 3µm, MeCN/water = 50/50 1 ml/min, 298K, 220nm

Peak	Retention time (min)	Area (%)
1	8.0	50.9
2	8.9	49.1
Total		100.0

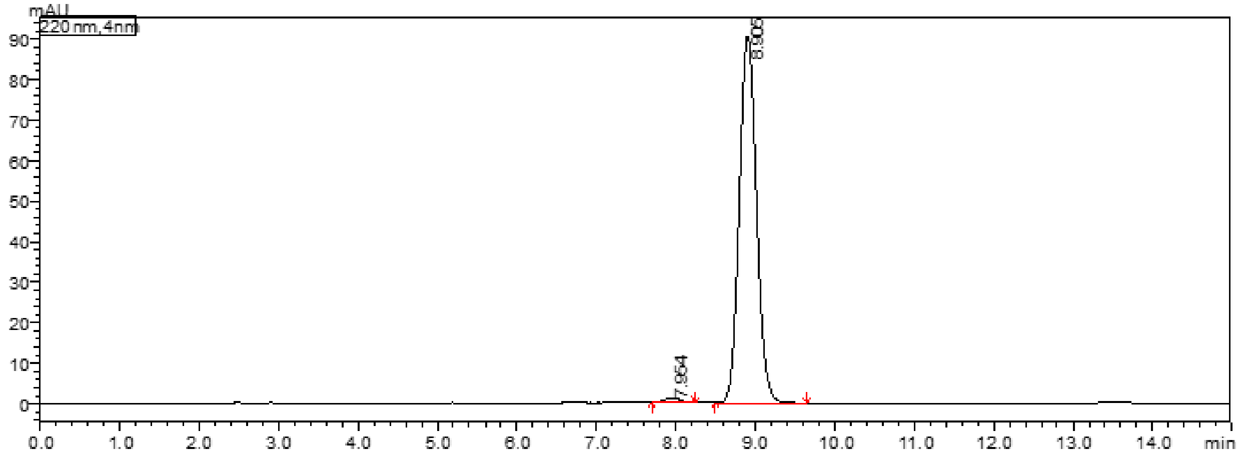

Peak	Retention time (min)	Area (%)
1	8.0	1.2
2	8.9	98.8
Total		100.0

HPLC traces of *rac*-**3p** and chiral **3p.**

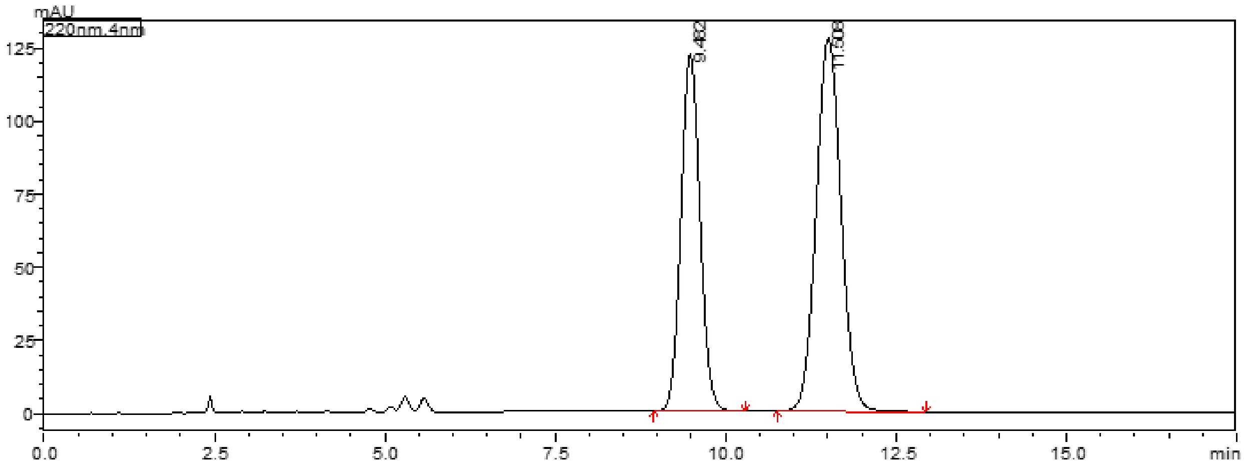

150 mm Chiralcel IC-3R, 4.6 mm i.D., 3µm, MeCN/water = 60/40 1 ml/min, 298K, 220nm

Peak	Retention time (min)	Area (%)
1	9.5	42.1
2	11.5	57.9
Total		100.0

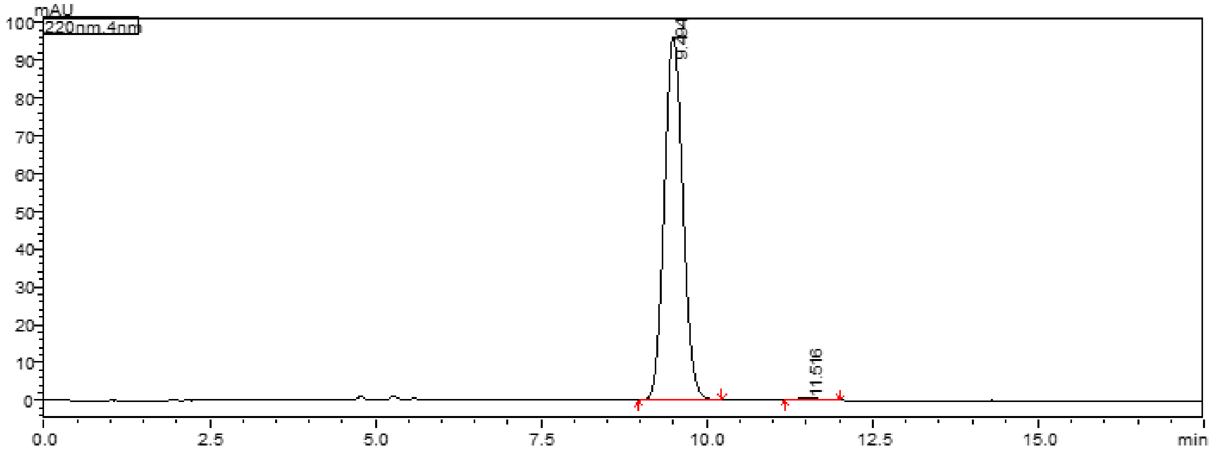

Peak	Retention time (min)	Area (%)
1	9.5	99.3
2	11.5	0.7
Total		100.0

HPLC traces of *rac*-**3q** and chiral **3q.**

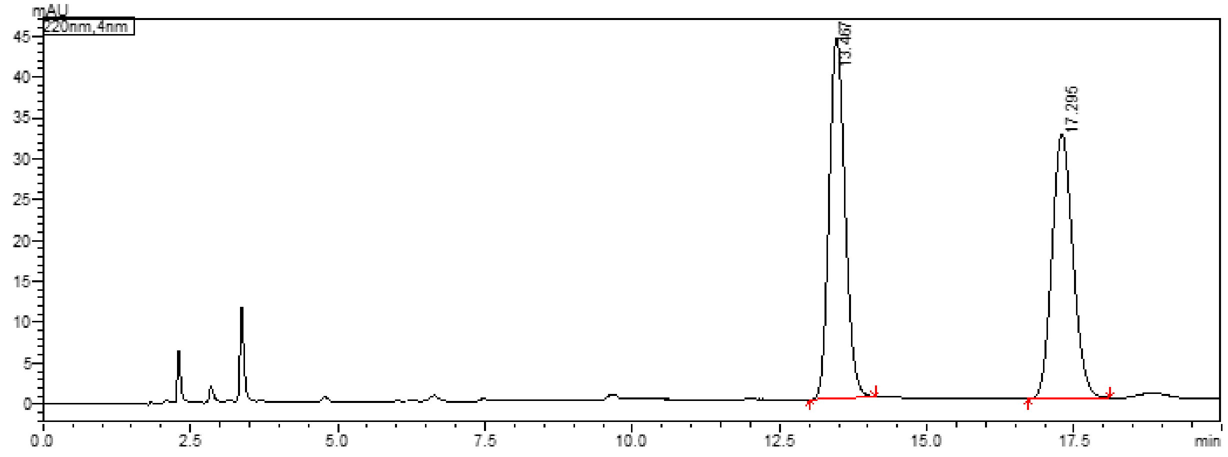

150 mm Chiralcel OJ-3R, 4.6 mm i.D., 3μm, MeCN/water = 60/40, 1 ml/min, 298K, 220nm

Peak	Retention time (min)	Area (%)
1	13.5	50.7
2	17.3	49.3
Total		100.0

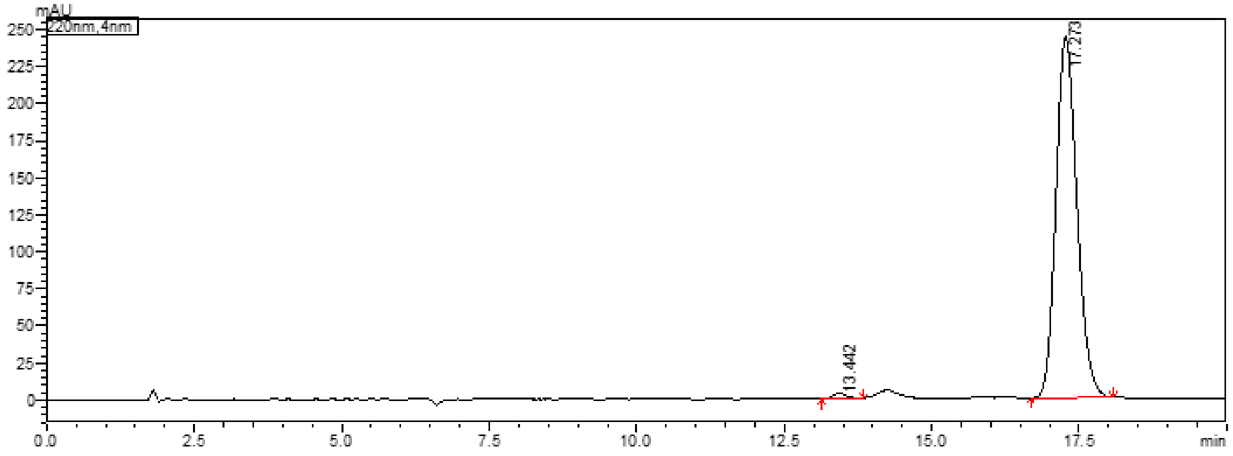

Peak	Retention time (min)	Area (%)
1	13.4	1.2
2	17.3	98.8
Total		100.0

HPLC traces of chiral **3q** from the 1,2-addition reaction of **3a**

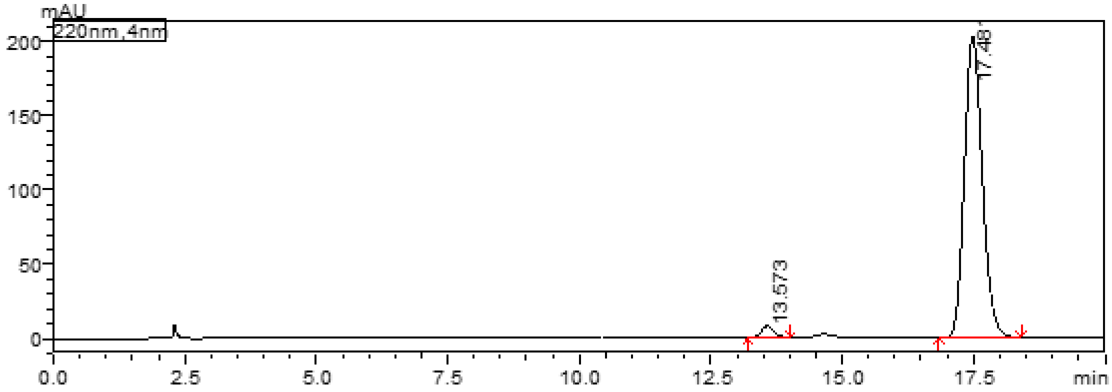

Peak	Retention time (min)	Area (%)
1	13.5	2.8
2	17.4	97.2
Total		100.0

HPLC traces of *rac*-**3r** and chiral **3r.**

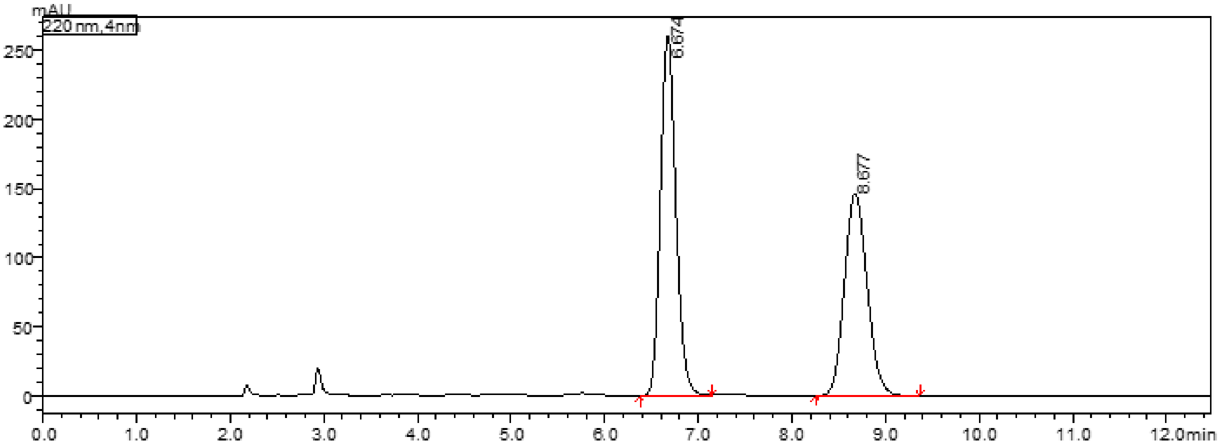

150 mm Chiralcel OJ-3R, 4.6 mm i.D., 3µm, MeCN/water = 70/30 1 ml/min, 298K, 220nm

Peak	Retention time (min)	Area (%)
1	6.7	54.7
2	8.7	44.3
Total		100.0

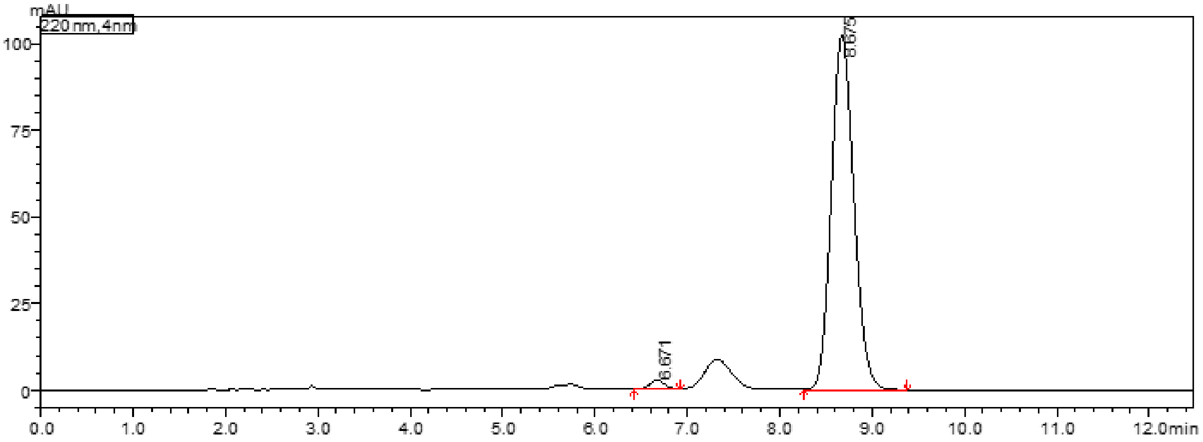

Peak	Retention time (min)	Area (%)
1	6.7	1.9
2	8.7	98.1
Total		100.0

HPLC traces of *rac*-**3s** and chiral **3s.**

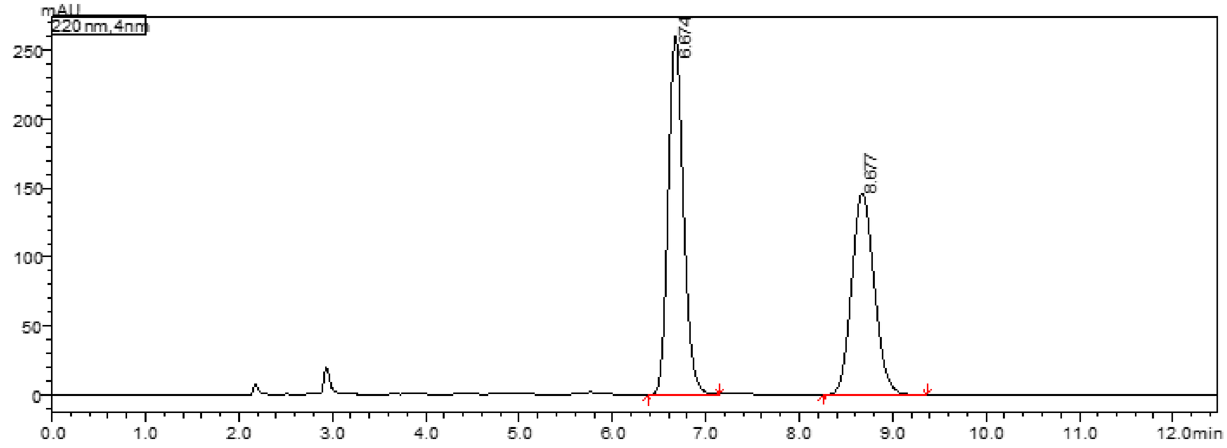

150 mm Chiralcel OJ-3R, 4.6 mm i.D., 3μm, MeCN/water = 70/30 1 ml/min, 298K, 220nm

Peak	Retention time (min)	Area (%)
1	6.7	54.7
2	8.7	44.3
Total		100.0

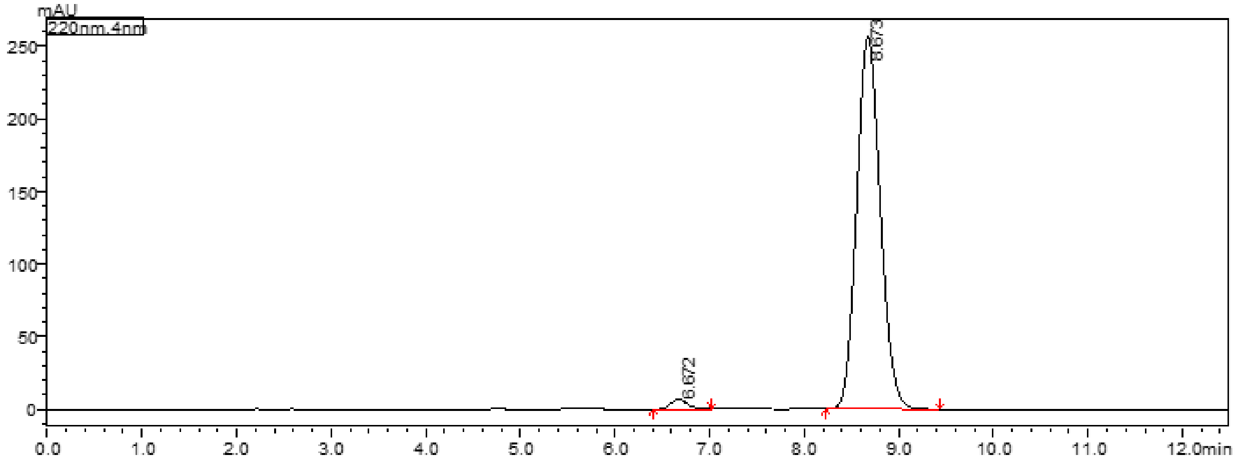

Peak	Retention time (min)	Area (%)
1	6.7	1.8
2	8.7	98.2
Total		100.0

HPLC traces of *rac*-**3t** and chiral **3t.**

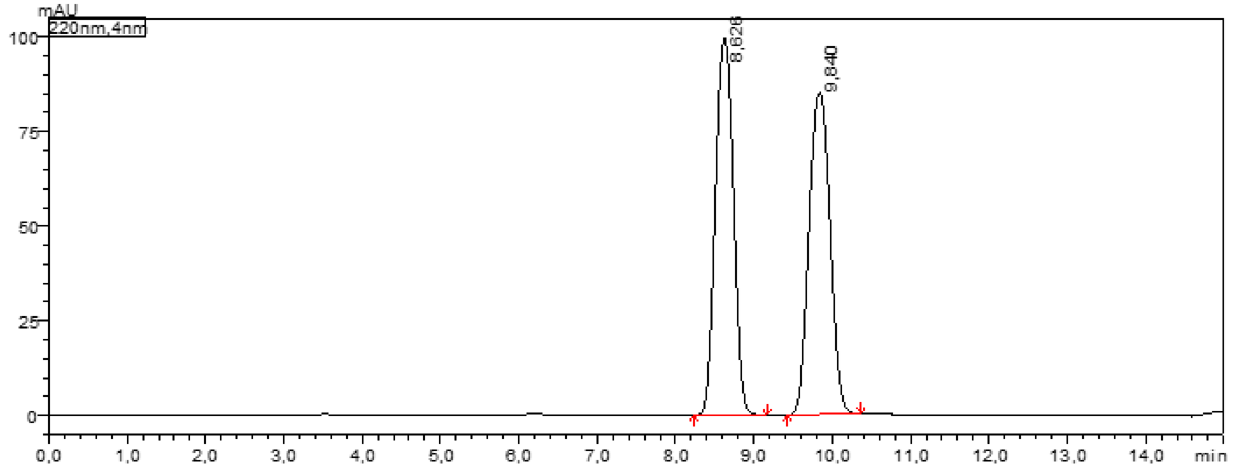

150 mm Chiralcel OJ-3R, 4.6 mm i.D., 3µm, MeCN/water = 60/40, 1 ml/min, 298K, 220nm

Peak	Retention time (min)	Area (%)
1	8.6	50.1
2	9.8	49.9
Total		100.0

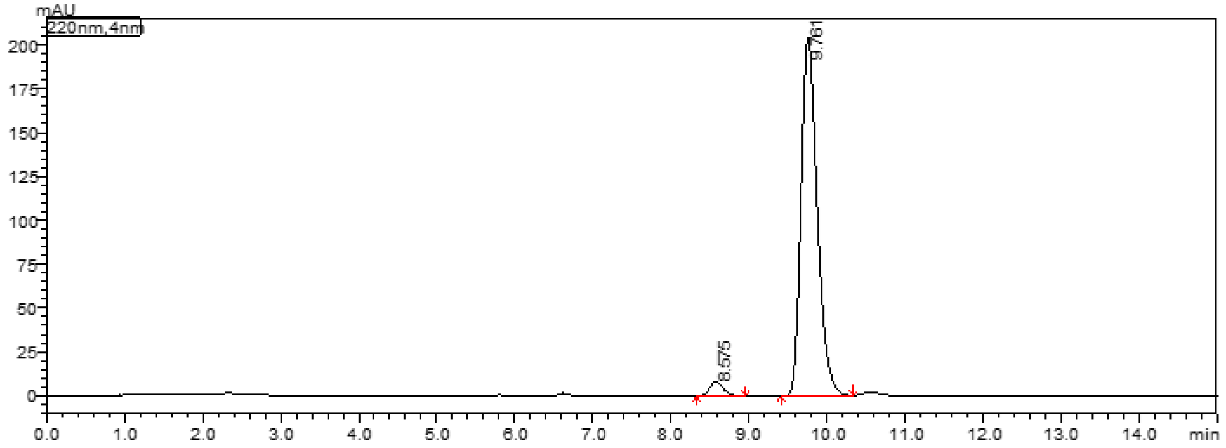

Peak	Retention time (min)	Area (%)
1	8.6	3.2
2	9.7	96.8
Total		100.0

10 References

1. Zhang, F.; Dutta, S.; Petti, A.; Rana, D.; Daniliuc, C. G.; Glorius, F., Solvent-Dependent Divergent Cyclization of Bicyclo[1.1.0]butanes. *Angew. Chem., Int. Ed.* **2025,** *64* , e202418239.
2. Ren, H.; Li, T.; Xing, J.; Li, Z.; Zhang, Y.; Yu, X.; Zheng, J., Ti-Catalyzed Formal [2π + 2σ] Cycloadditions of Bicyclo[1.1.0]butanes with 2-Azadienes to Access Aminobicyclo[2.1.1]hexanes. *Org. Lett.* **2024,** *26*, 1745-1750.
3. de Robichon, M.; Kratz, T.; Beyer, F.; Zuber, J.; Merten, C.; Bach, T., Enantioselective, Intermolecular [π2+σ2] Photocycloaddition Reactions of 2(1H)-Quinolones and Bicyclo[1.1.0]butanes. *J. Am. Chem. Soc.* **2023,** *145* , 24466-24470.
4. Dutta, S.; Lee, D.; Ozols, K.; Daniliuc, C. G.; Shintani, R.; Glorius, F., Photoredox-Enabled Dearomative [2π + 2σ] Cycloaddition of Phenols. *J. Am. Chem. Soc.* **2024,** *146*, 2789-2797.
5. Dhake, K.; Woelk, K. J.; Becica, J.; Un, A.; Jenny, S. E.; Leitch, D. C., Beyond Bioisosteres: Divergent Synthesis of Azabicyclohexanes and Cyclobutenyl Amines from Bicyclobutanes. *Angew. Chem., Int. Ed.* **2022,** *61* (27), e202204719.
6. Scharf, M. J.; List, B., A Catalytic Asymmetric Pictet–Spengler Platform as a Biomimetic Diversification Strategy toward Naturally Occurring Alkaloids. *J. Am. Chem. Soc.* **2022,** *144*, 15451-15456.
